# U.S. Selected Practice Recommendations for Contraceptive Use, 2024

**DOI:** 10.15585/mmwr.rr7303a1

**Published:** 2024-08-08

**Authors:** Kathryn M. Curtis, Antoinette T. Nguyen, Naomi K. Tepper, Lauren B. Zapata, Emily M. Snyder, Kendra Hatfield-Timajchy, Katherine Kortsmit, Megan A. Cohen, Maura K. Whiteman, Contributors, Courtney Baker, Divya Dethier, Sophia Garbarino, Heather Gold, Emma Halper, Nathalie Kapp, Gopika Krishna, Marielle Meurice, Stephanie Ramer, Jessica Rodenhizer, Nisha Verma, Steffanie Wright

**Affiliations:** 1 Division of Reproductive Health, National Center for Chronic Disease Prevention and Health Promotion, CDC; University of Texas Southwestern Medical Center, Dallas, Texas; University of Hawaii, Honolulu, Hawaii; Emory University, Atlanta, Georgia; Emory University, Atlanta, Georgia; Emory University, Atlanta, Georgia; International Planned Parenthood Federation, London, England; Columbia University, New York, New York; University of California-San Diego, San Diego, California; CDC, Atlanta, Georgia; CDC, Atlanta, Georgia; Emory University, Atlanta, Georgia; Harvard University, Boston, Massachusetts

## Abstract

*The 2024* U.S. Selected Practice Recommendations for Contraceptive Use *(U.S. SPR) addresses a selected group of common, yet sometimes complex, issues regarding initiation and use of specific contraceptive methods. These recommendations for health care providers were updated by CDC after review of the scientific evidence and a meeting with national experts in Atlanta, Georgia, during January 25–27, 2023. The information in this report replaces the 2016 U.S. SPR* (CDC. U.S. Selected Practice Recommendations for Contraceptive Use, 2016. MMWR 2016;65[No. RR-4]:1–66). *Notable updates include 1) updated recommendations for provision of medications for intrauterine device placement, 2) updated recommendations for bleeding irregularities during implant use, 3) new recommendations for testosterone use and risk for pregnancy, and 4) new recommendations for self-administration of injectable contraception. The recommendations in this report are intended to serve as a source of evidence-based clinical practice guidance for health care providers. The goals of these recommendations are to remove unnecessary medical barriers to accessing and using contraception and to support the provision of person-centered contraceptive counseling and services in a noncoercive manner. Health care providers should always consider the individual clinical circumstances of each person seeking contraceptive services. This report is not intended to be a substitute for professional medical advice for individual patients; when needed, patients should seek advice from their health care providers about contraceptive use.*

## Introduction

*U.S. Selected Practice Recommendations for Contraceptive Use, 2024* (U.S. SPR) provides recommendations for health care providers that address provision of contraceptive methods and management of side effects and issues related to contraceptive method use within the framework of removing unnecessary medical barriers to accessing and using contraception. U.S. SPR is a companion document to *U.S. Medical Eligibility Criteria for Contraceptive Use, 2024* (U.S. MEC) (*1*), which provides recommendations for safe use of contraceptive methods for persons with various medical conditions and other characteristics. Both U.S. MEC and U.S. SPR were adapted from global guidance developed by the World Health Organization (WHO) (*2*,*3*). WHO intended for the global guidance to be used by local or national policymakers, family planning program managers, and the scientific community as a reference when they develop family planning guidance at the country or program level (*3*). During 2012–2013, CDC went through a formal process to adapt the global guidance for use in the United States, which included rigorous identification and critical appraisal of the scientific evidence through systematic reviews and input from national experts on how to translate that evidence into recommendations for U.S. health care providers (*4*); a subsequent update was published in 2016 (*5*).

U.S. MEC and U.S. SPR recommendations are components of quality contraceptive services and can be used in conjunction with other guidance documents such as *Providing Quality Family Planning Services: Recommendations of CDC and the U.S. Office of Population Affairs*, which provides recommendations for the content and delivery of services related to preventing or for achieving pregnancy (*6*–*8*). Evidence-based guidance can support health care providers when providing person-centered counseling and contraceptive services, including assisting persons in selecting and using contraceptive methods safely and effectively.

Equitable access to the full range of contraceptive methods for all those seeking care is an essential component of high-quality sexual and reproductive health care. Contraceptive services should be offered in a noncoercive manner that supports a person’s values, goals, and reproductive autonomy through a shared decision-making process with health care providers (*9*–*13*). Because of the history of and ongoing forced sterilization and reproductive coercion in the United States among persons of racial and ethnic minority groups, persons with disabilities, and other groups that have been marginalized, it is important that persons can select the method that best meets their needs to promote reproductive autonomy (*9*–*13*).

This report replaces the 2016 version of U.S. SPR (*5*) with new and revised recommendations, on the basis of new evidence and input from experts. This updated document uses gender-inclusive language throughout. However, when summarizing published evidence that describes study populations by specific genders, the wording of the primary studies has been maintained for accuracy. Notable updates include 1) updated recommendations for provision of medications for intrauterine device (IUD) placement, 2) updated recommendations for bleeding irregularities during implant use, 3) new recommendations for testosterone use and risk for pregnancy, and 4) new recommendations for self-administration of injectable contraception. CDC reviewed and affirmed the recommendations for bleeding irregularities with levonorgestrel (LNG) IUD (LNG-IUD) use and for use of regular contraception after ulipristal acetate (UPA) for emergency contraception on the basis of updated systematic reviews of the evidence. These recommendations are meant to serve as a source of evidence-based clinical guidance for health care providers and can support the provision of person-centered contraceptive counseling and services in a noncoercive manner. Health care providers should always consider the individual clinical circumstances of each person seeking contraceptive services. This report is not intended to be a substitute for professional medical advice for individual patients; as needed, patients should seek advice from their health care providers about contraceptive use.

## Summary of Changes from the 2016 U.S. SPR

### Updated Recommendations

Recommendations for provision of medications for IUD placement and management of bleeding irregularities (including amenorrhea) during implant use have been updated from the 2016 U.S. SPR. Substantive modifications from the 2016 U.S. SPR are noted with an asterisk.

#### Provision of Medications for IUD Placement

- Misoprostol is not recommended for routine use for IUD placement. Misoprostol might be useful in selected circumstances (e.g., in patients with a recent failed placement).
- Lidocaine (paracervical block or topical) for IUD placement might be useful for reducing patient pain.*

#### Bleeding Irregularities (Including Amenorrhea) During Implant Use

- Before implant placement, provide counseling about potential changes in bleeding patterns during implant use. Spotting or light bleeding is common with implant use, and certain implant users experience amenorrhea. These bleeding changes are generally not harmful but might be bothersome to the patient. Bleeding changes might or might not decrease with continued implant use. Heavy bleeding is uncommon during implant use.

##### Bleeding Irregularities (Spotting, Light Bleeding, or Heavy or Prolonged Bleeding)

- If clinically indicated, consider an underlying health condition, such as interactions with other medications, sexually transmitted infections (STIs), pregnancy, thyroid disorders, or new pathologic uterine conditions (e.g., polyps or fibroids). If an underlying health condition is found, treat the condition or refer for care.
- Explore patient goals, including continued implant use (with or without treatment for bleeding irregularities) or implant removal. If the patient wants to continue implant use, provide reassurance, discuss options for management of bleeding irregularities if it is desired, and advise the patient that they may contact their provider at any time to discuss bleeding irregularities or other side effects.
- If the patient desires implant removal at any time, remove the implant, offer counseling on alternative contraceptive methods, and initiate another method if it is desired.
- If the patient wants treatment, the following treatment options may be considered, depending on the patient’s preferences, treatment goals, and medical history:*
  º Treatments that might improve bleeding irregularities during treatment use; bleeding is likely to recur after treatment cessation. Treatment may be repeated as needed.*
    - Hormonal treatment (e.g., 20–30 *µ*g ethinyl estradiol [EE] combined oral contraceptives [COCs] or estrogen)*
    - Antifibrinolytic agents (e.g., tranexamic acid), 5 days*
  º Treatments that might improve bleeding irregularities during treatment use and whose effects might persist for some time after treatment cessation. Treatment may be repeated as needed.*
    - Nonsteroidal anti-inflammatory drugs (NSAIDs) (e.g., celecoxib, ibuprofen, or mefenamic acid), 5–7 days*
    - Selective estrogen receptor modulators (SERMs) (e.g., tamoxifen), 7–10 days*

##### Amenorrhea

- Amenorrhea does not require any medical treatment. Provide reassurance.
  º If a patient’s regular bleeding pattern changes abruptly to amenorrhea, consider ruling out pregnancy if clinically indicated.
  º If the patient desires implant removal, remove the implant, offer counseling on alternative contraceptive methods, and initiate another method if it is desired.

### New Recommendations

Recommendations for testosterone use and risk for pregnancy and self-administration of injectable contraception have been added to the U.S. SPR.

#### Testosterone Use and Risk for Pregnancy

- Counsel that testosterone use might not prevent pregnancy among transgender, gender diverse, and nonbinary persons with a uterus who are using testosterone. Offer contraceptive counseling and services to those who are at risk for and do not desire pregnancy.*

#### Self-Administration of Subcutaneous Injectable Contraception

- Self-administered subcutaneous depot medroxyprogesterone acetate (DMPA-SC) should be made available as an additional approach to deliver injectable contraception.* (This recommendation was developed and published in 2021) (*14*).

## Methods

Since publication of the 2016 U.S. SPR, CDC has monitored the literature for new evidence relevant to the recommendations through the WHO/CDC Continuous Identification of Research Evidence (CIRE) system (*15*). This system identifies new evidence as it is published and allows WHO and CDC to update systematic reviews and facilitate updates to recommendations as new evidence warrants. Automated searches are run in PubMed weekly, and the results are reviewed. Abstracts that meet specific criteria are added to the web-based CIRE system, which facilitates coordination and peer review of systematic reviews for both WHO and CDC. For this update, CDC reviewed all existing recommendations in the 2016 U.S. SPR for new evidence identified by CIRE that had the potential to lead to a changed recommendation. To obtain comments from the public about revisions to CDC’s contraception recommendations (U.S. MEC and U.S. SPR), CDC published a notice in the Federal Register (86 FR 46703) on August 19, 2021, requesting public comment on content to consider for revision or addition to the recommendations and how to improve the implementation of the guidance documents (*16*). The comment period closed on October 18, 2021. CDC received 46 submissions from the general public, including private persons, professional organizations, academic institutions, and industry. CDC reviewed each of the submissions and carefully considered them when revising the recommendations.

During January 25–26, 2022, CDC held virtual scoping meetings with 18 participants who were invited to provide their individual input on the scope for updating the 2016 U.S. SPR. The 18 invited participants represented various types of health care providers and health care provider organizations. Lists of participants and potential conflicts of interests are provided at the end of this report. Meeting participants discussed topics to be addressed in the update of U.S. SPR based on the presentation of new evidence published since 2016 (identified through the CIRE system), submissions received through the Federal Register notice, and feedback CDC received from other sources (e.g., health care providers and others through email, public inquiry, and questions received at conferences). CDC identified multiple topics to consider when updating the guidance, including revision of existing U.S. SPR recommendations (provision of medications for IUD placement, bleeding irregularities during LNG-IUD use and implant use, and hormonal contraception after use of UPA for emergency contraception) and addition of a new U.S. SPR recommendation (testosterone use and risk for pregnancy). CDC determined that all other recommendations in the 2016 U.S. SPR were up to date and consistent with the existing body of evidence for that recommendation.

In preparation for a subsequent expert meeting held January 25–27, 2023, to review the scientific evidence for potential recommendations, CDC staff members and other invited authors conducted systematic reviews for each of the topics being considered. The purpose of these systematic reviews was to identify direct and indirect evidence for use in developing or updating recommendations on provision of contraceptive methods and issues related to contraceptive method use. Person-centered outcomes that might represent contraceptive users’ values and preferences (e.g., method continuation and patient satisfaction) were considered where relevant and available for each of the systematic reviews. Preferred Reporting Items for Systematic Reviews and Meta-Analyses (PRISMA) guidelines were followed for reporting systematic reviews (*17*). The Grading of Recommendations Assessment, Development and Evaluation (GRADE) approach was used to assess the certainty of the evidence (*18*,*19*). Certainty of evidence was rated as high, moderate, low, or very low depending on criteria including study design, risk for bias, indirectness, imprecision, and inconsistency. Outcomes evaluated in randomized clinical trials (RCTs) are considered to have high certainty of evidence and those in observational studies to have low certainty; these ratings are adjusted according to the previously mentioned criteria. When direct evidence was limited or not available, indirect evidence (e.g., evidence on proxy outcomes) and theoretical issues were considered. Reviews are referenced and cited throughout this report; the full reviews will be submitted to peer-reviewed journals and will contain the details of each review, including the systematic review question, literature search protocol (registered in https://www.crd.york.ac.uk/PROSPERO), inclusion and exclusion criteria, evidence tables, and quality assessments. Brief summaries of the evidence and GRADE tables are included (Supplementary Appendix, https://stacks.cdc.gov/view/cdc/156517). CDC staff continued to monitor new evidence identified through the CIRE system during the preparation for the January 2023 meeting.

In addition to the preparation of the systematic reviews, CDC included patient perspectives in the guideline update process to better consider how the resulting updated recommendations could meet patient preferences and needs. Consideration of patient perspectives can center discussions on the evidence in a person-centered care model, can support inclusion of patient perspectives along with provider perspectives on the evidence, and has the potential to shape recommendations. In November and December 2022, listening sessions were held with a different group of 18 participants, representing themselves or patient advocacy organizations, who provided perspectives from patient populations such as youths; lesbian, gay, bisexual, transgender, queer, and intersex (LGBTQI+) persons; persons with disabilities; and persons with chronic medical conditions. The goal of the listening sessions was to gather insights about participants’ experiences, values, preferences, and information needs related to contraceptive method use and decision-making.

During January 25–27, 2023, in Atlanta, Georgia, CDC held a meeting with 40 participants who were invited to provide their individual perspectives on the scientific evidence presented and the implications for practice for U.S. SPR. Thirty-eight participants represented a wide range of expertise in contraception provision, research, and reproductive justice and included obstetricians and gynecologists, pediatricians, family physicians, internal medicine physicians, nurse practitioners, epidemiologists, and others with research and clinical practice expertise in contraceptive safety, effectiveness, and management. Two participants were patient representatives who provided their individual perspectives on the topics discussed throughout the meeting. During the meeting, a summary of the information from the patient listening sessions was presented, and the two patient representatives presented information on their individual experiences and perspectives related to receipt of contraceptive services. The evidence from the systematic review for each topic was presented, including direct evidence and any indirect evidence or theoretical concerns. Meeting participants provided their individual perspectives on topics discussed throughout the meeting and on using the evidence to develop recommendations that would meet the needs of U.S. health care providers and the patients they serve. Participants also provided feedback on the certainty of evidence, the balance of benefits and harms, and values and preferences. Areas of research that need additional investigation also were considered during the meeting. Lists of participants and potential conflicts of interest are provided at the end of this report.

After the meeting in January 2023, CDC determined the recommendations in this report, taking into consideration the individual perspectives provided by the meeting participants. Feedback also was received from a group of four external reviewers, composed of health care providers and researchers who had not participated in the scoping or update meetings. These external reviewers were asked to provide comments on the accuracy, feasibility, and clarity of the recommendations.

## Keeping Guidance Up to Date

As with any evidence-based guidance document, a key challenge is keeping the recommendations up to date as new scientific evidence becomes available. Working with WHO, CDC uses the CIRE system to ensure that WHO and CDC guidance is based on the best available evidence and that a mechanism is in place to update guidance when new evidence becomes available (*15*). CDC will continue to work with WHO to identify and assess all new relevant evidence and determine whether changes in the recommendations are warranted. CDC will completely review U.S. SPR periodically. Updates to the guidance will be published in CDC’s *Morbidity and Mortality Weekly Report* (*MMWR*) and posted on the CDC website (https://www.cdc.gov/contraception/hcp/contraceptive-guidance).

As part of the process to update these recommendations, CDC identifies gaps in the evidence for the recommendations considered. Evidence might be limited on interventions for addressing issues with contraceptive method use. Generalizability of the published evidence to all persons seeking contraceptive services presents a challenge because of biases about who might be included in studies on contraceptive safety. New, high-quality research on contraception that addresses priority research gaps inclusive of diverse populations can further strengthen these recommendations and improve clinical practice.

## How To Use This Document

The recommendations in this report are intended to help health care providers address provision of contraceptive methods and management of side effects and issues related to contraceptive method use, such as how to help patients initiate use of a contraceptive method; which examinations and tests are needed before initiating use of a contraceptive method; what regular follow-up is needed; and how to address problems that often arise during use, including missed pills and side effects such as bleeding irregularities. Use of evidence-based recommendations by health care providers can remove unnecessary medical barriers and help patients access and successfully use contraceptive methods. Multiple medical barriers to initiating and continuing contraceptive methods might exist, such as unnecessary screening examinations and tests before starting the method (e.g., a pelvic examination before initiation of COCs), inability to receive the contraceptive on the same day as the visit (e.g., waiting for test results that might not be needed or waiting until the patient’s next menstrual cycle to start use), and difficulty obtaining continued contraceptive supplies (e.g., restrictions on number of pill packs prescribed or dispensed at one time or requiring unnecessary follow-up procedures) (*20*–*24*). Removing unnecessary steps can help patients access and successfully use contraception.

Each U.S. SPR recommendation addresses what a patient or health care provider can do in specific situations. Health care providers can also use the U.S. MEC to determine medical eligibility for use of specific contraceptive methods on the basis of a patient’s characteristics and medical conditions (*1*). The full U.S. MEC recommendations and the evidence supporting those recommendations were updated in 2024 (*1*) and are summarized (Appendix A).

The recommendations in this report are not intended to provide guidance on every aspect of provision and management of contraceptive method use. Instead, they incorporate the best available evidence to address specific issues regarding common, yet sometimes complex, issues regarding initiation and use of specific contraceptive methods. Each contraceptive method section generally includes information about initiation of the method, regular follow-up, and management of problems with use (e.g., usage errors and side effects). Each section first provides the recommendation and then includes comments and a brief summary of the scientific evidence on which the recommendation is based. The level or certainty of evidence from the systematic reviews for each evidence summary is provided. For recommendations developed before 2024, the level of evidence was determined using the U.S. Preventive Services Task Force system, which includes ratings for study design (I: randomized controlled trials; II-1: controlled trials without randomization; II-2: observational studies; and II-3: multiple time series or descriptive studies), ratings for internal validity (good, fair, or poor), and categorization of the evidence as direct or indirect for the specific review question (*25*). For recommendations developed or revised in this updated publication, the certainty of evidence for each outcome was assessed as high, moderate, low, or very low using the GRADE approach (*18*,*19*).

The information in this report is organized by contraceptive method. Recommendations are provided for permanent methods of contraception (tubal surgery and vasectomy) and for reversible methods of contraception, including the copper (380 mm^2^) IUD (Cu-IUD) and LNG (13.5 mg, 19.5 mg, or 52 mg) IUD; the etonogestrel (ENG) implant; progestin-only injectables (depot medroxyprogesterone acetate [DMPA]); progestin-only pills (POPs; norethindrone, norgestrel, and drospirenone); combined hormonal contraceptives (CHCs) that contain both estrogen and a progestin, including COCs, combined transdermal patches, and combined vaginal rings; and the standard days method (SDM). Recommendations also are provided for emergency use of the Cu-IUD and emergency contraceptive pills (ECPs).

For each contraceptive method, recommendations are provided on the timing for initiation of the method and indications for when and for how long additional contraception, or a back-up method, is needed. Many of these recommendations include guidance that a patient may start a contraceptive method at any time during their menstrual cycle, if it is reasonably certain that they are not pregnant. Guidance for health care providers also is provided on how to be reasonably certain that a patient is not pregnant, testosterone use and risk for pregnancy, and when contraceptive protection is no longer needed.

For each contraceptive method, recommendations include the examinations and tests needed before initiation of the method. These recommendations apply to patients who are presumed to be healthy. Most patients need no or very few examinations or tests before initiating a contraceptive method although examinations or tests might be needed to address other noncontraceptive health needs (*6*). Patients with known medical problems or other special conditions might need additional examinations or tests before being determined to be appropriate candidates for a particular method of contraception. U.S. MEC might be useful in such circumstances (*1*). Any additional screening needed for preventive health care can be performed at the time of contraception initiation, and initiation should not be delayed for test results. The following classification system was developed by WHO and adopted by CDC to categorize the applicability of the various examinations or tests before initiation of contraceptive methods (*26*):

**Class A:** These tests and examinations are essential and mandatory in all circumstances for safe and effective use of the contraceptive method.
**Class B:** These tests and examinations contribute substantially to safe and effective use, although implementation may be considered within the public health context, service context, or both. The risk of not performing an examination or test should be balanced against the benefits of making the contraceptive method available.
**Class C:** These tests and examinations do not contribute substantially to safe and effective use of the contraceptive method.

These classifications focus on the relation of the examinations or tests to safe initiation of a contraceptive method. They are not intended to address the appropriateness of these examinations or tests in other circumstances. For example, certain examinations or tests that are not deemed necessary for safe and effective contraceptive use might be appropriate for quality preventive health care or for diagnosing or assessing suspected medical conditions. Systematic reviews were conducted for multiple different types of examinations and tests to assess whether a screening test was associated with safe use of contraceptive methods. Because no single convention exists for screening panels for certain diseases (e.g., diabetes, lipid disorders, and liver diseases), the search strategies included broad terms for the tests and diseases of interest.

Summary charts and clinical algorithms that summarize the guidance for the various contraceptive methods have been developed for many of the recommendations, including when to start using specific contraceptive methods (Appendix B), examinations and tests needed before initiating the various contraceptive methods (Appendix C), routine follow-up after initiating contraception (Appendix D), management of bleeding irregularities among users of specific contraceptive methods (Appendix E), and management of IUDs when users are found to have pelvic inflammatory disease (PID) (Appendix F). Additional tools are available on the CDC website (https://www.cdc.gov/contraception/hcp/contraceptive-guidance).

## Contraceptive Decision-Making

CDC acknowledges the paramount importance of personal autonomy in contraceptive decision-making. This is critically important because of the context of historical and ongoing contraceptive coercion and reproductive mistreatment in the United States, especially among communities that have been marginalized, including human rights violations such as forced sterilization and enrollment in contraceptive trials without informed consent (*10*,*11*,*13*). Coercive practices in the health care system can include provider bias for certain contraceptive methods over a patient’s reproductive goals and preferences, lack of person-centered counseling and support, and policies or incentives for uptake of certain contraceptive methods (*11*). For health care providers and the settings in which they work, it is important to acknowledge the structural systems that drive inequities (e.g., discrimination because of race, ethnicity, disability, sex, gender, and sexual orientation), work to mitigate harmful impacts, and recognize that provider bias (unconscious or explicit) might affect contraceptive counseling and provision of services (*13*). All persons seeking contraceptive care need access to appropriate counseling and services that support the person’s values, goals, and reproductive autonomy (*9*–*13*). Health care providers can support the contraceptive needs of all persons by using a person-centered framework and recognizing the many factors that influence individual decision-making about contraception (*10*,*12*,*13*).

U.S. MEC and U.S. SPR recommendations can be used to support a person’s contraceptive decision-making (Box 1). Persons should have equitable access to the full range of contraceptive methods and be given the information they need for contraceptive decision-making in a noncoercive manner. Patient-centeredness has been defined by the Institute of Medicine as “providing care that is respectful of and responsive to individual patient preferences, needs, and values and ensuring that patient values guide all clinical decisions” (*27*). Shared decision-making and person-centered approaches to providing health care recognize the expertise of both the medical provider and the patient (*10*,*13*,*27*).

BOX 1 **Using the *U.S. Medical Eligibility Criteria for Contraceptive Use* and *U.S. Selected Practice Recommendations for Contraceptive Use* recommendations to support contraceptive decision-making**

- CDC acknowledges the paramount importance of personal autonomy in contraceptive decision-making.
- Persons should have equitable access to the full range of contraceptive methods.
- Contraceptive services should be offered in a noncoercive manner that supports a person’s values, goals, and reproductive autonomy.
- Shared decision-making and person-centered approaches recognize the expertise of both the health care provider and the person.
- A person-centered approach to contraceptive decision-making
  º prioritizes a person’s preferences and reproductive autonomy rather than a singular focus on pregnancy prevention,
  º respects the person as the main decision-maker in contraceptive decisions, and
  º includes respecting the decision not to use contraception or to discontinue contraceptive method use.
- U.S. MEC and U.S. SPR recommendations can be used by health care providers to support persons in contraceptive decision-making.
- U.S. MEC and U.S. SPR recommendations can be used by health care providers to remove unnecessary medical barriers to accessing and using contraception.

**Abbreviations:** U.S. MEC = *U.S. Medical Eligibility Criteria for Contraceptive Use*; U.S. SPR = *U.S. Selected Practice Recommendations for Contraceptive Use.*

Health care providers should always consider the individual clinical and social factors of each person seeking contraceptive services and discuss reproductive desires, expectations, preferences, and priorities regarding contraception. A person might consider and prioritize many elements when choosing an acceptable contraceptive method, such as safety, effectiveness (*28*), availability (including accessibility and affordability), side effects, user control, reversibility, and ease of removal or discontinuation. Although most contraceptive methods are safe for use by most persons, U.S. MEC provides recommendations for the safety of specific contraceptive methods for persons with certain characteristics and medical conditions (*1*); a U.S. MEC summary (Appendix A) and the categories of medical eligibility criteria (U.S. MEC 1–4) for contraceptive use (Box 2) are provided. In addition, a person’s health risks associated with pregnancy and access to comprehensive health care services should be considered in these discussions. A person-centered approach to contraceptive decision-making prioritizes a person’s preferences and reproductive autonomy rather than a singular focus on pregnancy prevention and respects the person as the main decision-maker in contraceptive decisions, including the decision not to use contraception or to discontinue contraceptive method use (*13*,*29*). Voluntary informed choice of contraceptive methods is an essential guiding principle, and contraceptive counseling, where applicable, might be an important contributor to the successful use of contraceptive methods. Key resources provide additional information on person-centered contraceptive counseling and care (*6*,*10*,*13*,*30*).

BOX 2 **Categories of medical eligibility criteria for contraceptive use**

U.S. MEC 1 = A condition for which there is no restriction for the use of the contraceptive method

U.S. MEC 2 = A condition for which the advantages of using the method generally outweigh the theoretical or proven risks

U.S. MEC 3 = A condition for which the theoretical or proven risks usually outweigh the advantages of using the method

U.S. MEC 4 = A condition that represents an unacceptable health risk if the contraceptive method is used

**Source:** Nguyen AT, Curtis KM, Tepper NK, et al. U.S. medical eligibility criteria for contraceptive use, 2024. MMWR Recomm Rep 2024;73(No. RR-4):1–126.

**Abbreviation:** U.S. MEC = *U.S. Medical Eligibility Criteria for Contraceptive Use*.

## Prevention of Sexually Transmitted Infections

All patients, regardless of contraceptive choice, should be counseled about the use of condoms and the risk for STIs, including HIV infection (*31*). Most contraceptive methods, such as hormonal methods, IUDs, and permanent contraception, do not protect against STIs, including HIV infection. Consistent and correct use of external (male) latex condoms reduces the risk for STIs, including HIV infection (*31*). Although evidence is limited, use of internal (female) condoms can provide protection from acquisition and transmission of STIs (*31*). Patients also should be counseled that pre-exposure prophylaxis (PrEP), when taken as prescribed, is highly effective for preventing HIV infection (*32*). Additional information about prevention and treatment of STIs is available from CDC’s *Sexually Transmitted Infections Treatment Guidelines* (https://www.cdc.gov/std/treatment-guidelines/default.htm) (*31*), and information on PrEP for prevention of HIV infection is available from the U.S. Public Health Service’s *Preexposure Prophylaxis for the Prevention of HIV Infection in the United States — 2021 Update: A Clinical Practice Guideline* (https://www.cdc.gov/hiv/pdf/risk/prep/cdc-hiv-prep-guidelines-2021.pdf) (*32*).

## How To Be Reasonably Certain that a Patient Is Not Pregnant

In most cases, a detailed history provides the most accurate assessment of pregnancy risk in a patient who is about to start using a contraceptive method. Multiple criteria for assessing pregnancy risk are listed in the recommendation that follows (Box 3). These criteria are highly accurate (i.e., a negative predictive value of 99%–100%) in ruling out pregnancy among patients who are not pregnant (*33*–*36*). Therefore, CDC recommends that health care providers use these criteria to assess pregnancy status in a patient who is about to start using contraceptives. If a patient meets one of these criteria (and therefore the health care provider can be reasonably certain that the patient is not pregnant), a urine pregnancy test might be considered in addition to these criteria (based on clinical judgment), bearing in mind the limitations of the accuracy of pregnancy testing. If a patient does not meet any of these criteria, then the health care provider cannot be reasonably certain that the patient is not pregnant, even with a negative pregnancy test. Routine pregnancy testing for every patient is not necessary.

BOX 3 **How to be reasonably certain that a patient is not pregnant**

A health care provider can be reasonably certain that a patient is not pregnant if the patient has no symptoms or signs of pregnancy and meets any one of the following criteria:

- is ≤7 days after the start of normal menses
- has not had sexual intercourse since the start of last normal menses
- has been correctly and consistently using a reliable method of contraception
- is ≤7 days after spontaneous or induced abortion
- is within 4 weeks postpartum
- is fully or nearly fully breastfeeding (exclusively breastfeeding or the vast majority [≥85%] of feeds are breastfeeds), amenorrheic, and <6 months postpartum

On the basis of clinical judgment, health care providers might consider the addition of a urine pregnancy test; however, providers should be aware of the limitations, including accuracy of the test relative to the time of last sexual intercourse, recent delivery, or spontaneous or induced abortion. If a patient has had recent (i.e., within the past 5 days) unprotected sexual intercourse, consider offering emergency contraception (either a Cu-IUD or ECPs) if pregnancy is not desired (*1*).

**Comments and Evidence Summary.** The criteria for determining whether a patient is pregnant depend on the assurance that the patient has not ovulated within a certain amount of time after their last menses, spontaneous or induced abortion, or delivery. Among menstruating patients, the timing of ovulation can vary widely. During an average 28-day cycle, ovulation generally occurs during days 9–20 (*37*). In addition, the likelihood of ovulation is low from days 1–7 of the menstrual cycle (*38*). After a spontaneous or an induced abortion, ovulation can occur within 2–3 weeks and has been found to occur as early as 8–13 days after the end of the pregnancy. Therefore, the likelihood of ovulation is low ≤7 days after an abortion (*39*–*41*). A systematic review reported that the mean day of first ovulation among postpartum nonlactating women occurred 45–94 days after delivery (*42*). In one study, the earliest ovulation was reported at 25 days after delivery. Among women who are within 6 months postpartum, are fully or nearly fully breastfeeding (exclusively breastfeeding or the vast majority [≥85%] of feeds are breastfeeds), and are amenorrheic, the risk for pregnancy is <2% (*43*,*44*).

Although pregnancy tests often are performed before initiating contraception, the accuracy of qualitative urine pregnancy tests varies depending on the timing of the test relative to missed menses, recent sexual intercourse, or recent pregnancy. The sensitivity of a pregnancy test is defined as the concentration of human chorionic gonadotropin (hCG) at which 95% of tests are positive. Most qualitative pregnancy tests approved by the Food and Drug Administration (FDA) report a sensitivity of 20–25 mIU/mL in urine (*45*–*48*). However, pregnancy detection rates can vary widely because of differences in test sensitivity and the timing of testing relative to missed menses (*47*,*49*). Certain studies have demonstrated that an additional 11 days past the day of expected menses are needed to detect 100% of pregnancies using qualitative tests (*46*). In addition, pregnancy tests cannot detect a pregnancy resulting from recent sexual intercourse. Qualitative tests also might have positive results for several weeks after termination of pregnancy because hCG can be present for several weeks after delivery or abortion (spontaneous or induced) (*50*–*52*).

For contraceptive methods other than IUDs, the benefits of starting to use a contraceptive method likely exceed any risk, even in situations in which the health care provider is uncertain whether the patient is pregnant. Therefore, the health care provider can consider having patients start using contraceptive methods other than IUDs at any time, with a follow-up pregnancy test in 2–4 weeks. The risks for not starting to use contraception should be weighed against the risks for initiating contraception use in a patient who might be already pregnant. Most studies have demonstrated no increased risk for adverse outcomes, including congenital anomalies or neonatal or infant death, among infants exposed in utero to COCs (*53*–*55*). Studies also have demonstrated no increased risk for neonatal or infant death or developmental abnormalities among infants exposed in utero to DMPA (*54*,*56*,*57*).

In contrast, for patients who want to begin using an IUD (Cu-IUD or LNG-IUD), in situations in which the health care provider is uncertain whether the patient is pregnant, the patient should be provided with another contraceptive method to use until the health care provider is reasonably certain that they are not pregnant and can place the IUD. Pregnancies among women with IUDs are at higher risk for complications such as spontaneous abortion, septic abortion, preterm delivery, and chorioamnionitis (*58*).

A systematic review identified four analyses of data from three diagnostic accuracy studies that evaluated the performance of the listed criteria (Box 3) through use of a pregnancy checklist compared with a urine pregnancy test conducted concurrently (*59*). The performance of the checklist to diagnose or exclude pregnancy varied, with sensitivity of 55%–100% and specificity of 39%–89%. The negative predictive value was consistent across studies at 99%–100%, indicating the pregnancy checklist correctly ruled out women who were not pregnant. One of the studies assessed the added usefulness of signs and symptoms of pregnancy and found that these criteria did not substantially improve the performance of the pregnancy checklist, although the number of women with signs and symptoms was small (*33*) (Level of evidence: diagnostic accuracy studies, fair, direct).

## Testosterone Use and Risk for Pregnancy

- Counsel that testosterone use might not prevent pregnancy among transgender, gender diverse, and nonbinary persons with a uterus who are using testosterone. Offer contraceptive counseling and services to those who are at risk for and do not desire pregnancy.

**Comments and Evidence Summary**. Transgender, gender diverse, and nonbinary persons assigned female sex at birth often have a uterus, ovaries, and fallopian tubes (*60*). In a national survey of transgender, gender diverse, and nonbinary persons assigned female or intersex at birth, 54% of pregnancies were reported to be unintended, 61% of respondents did not want to be pregnant in the future, and 11% of respondents considered themselves to be at risk for pregnancy when they did not want to be pregnant (*61*). Some transgender, gender diverse, and nonbinary persons use testosterone for gender-affirming hormone therapy. Although certain regimens of testosterone might suppress fertility, testosterone therapy has not been studied as contraception. Testosterone is teratogenic and might have androgenic effects on fetal genitalia, reproductive systems, or endocrine systems (*62*). Addressing contraceptive needs of transgender, gender diverse, and nonbinary persons with a uterus, who are using testosterone, is critical to support reproductive autonomy among those not desiring pregnancy. Evidence on the safety and effectiveness of hormonal contraceptive use among transgender, gender diverse, and nonbinary persons with a uterus who are using testosterone is limited (*63*). Professional organizations provide information on contraceptive and reproductive health care for transgender, gender diverse, and nonbinary persons (*63*–*67*).

A systematic review identified one study that assessed risk for pregnancy among transgender, gender diverse, and nonbinary persons assigned female sex at birth using testosterone (*68*) (Supplementary Appendix, https://stacks.cdc.gov/view/cdc/156517). This noncomparative study followed 16 continuing testosterone users and six new testosterone users (who started testosterone at the beginning of the study) for 12 weeks and assessed the occurrence of ovulation as a proxy measure of risk for pregnancy through daily urine samples; ovulation was defined as urinary pregnanediol-3-glucuronide (PdG) >5 *µ*g/mL for 3 days. One (5%) participant ovulated, who was a new testosterone user. When using a lower threshold of PdG >3 *µ*g/mL for 2 days, 36% of participants ovulated (100% of new users and 13% of continuing users) (Certainty of evidence: very low).

## Intrauterine Contraception

Four IUDs are available in the United States: one copper (380 mm^2^) IUD and three LNG (13.5 mg, 19.5 mg, or 52 mg) IUDs. Fewer than one IUD user out of 100 becomes pregnant in the first year with typical use (*28*). IUDs are long-acting, are reversible, and can be used by patients of all ages, including adolescents, and by parous and nulliparous patients. IUDs do not protect against STIs, including HIV infection, and patients using IUDs should be counseled that consistent and correct use of external (male) latex condoms reduces the risk for STIs, including HIV infection (*31*). Use of internal (female) condoms can provide protection from STIs, including HIV infection, although data are limited (*31*). Patients also should be counseled that PrEP, when taken as prescribed, is highly effective for preventing HIV infection (*32*).

### Initiation of Cu-IUDs

#### Timing

- The Cu-IUD may be placed at any time if it is reasonably certain that the patient is not pregnant (Box 3).
- The Cu-IUD also may be placed within 5 days of the first act of unprotected sexual intercourse as an emergency contraceptive. If the day of ovulation can be estimated, the Cu-IUD also may be placed >5 days after sexual intercourse as long as placement does not occur >5 days after ovulation.

#### Need for Back-Up Contraception

- No additional contraceptive protection is needed after Cu-IUD placement.

#### Special Considerations

##### Amenorrhea (Not Postpartum)

- **Timing:** The Cu-IUD may be placed at any time if it is reasonably certain that the patient is not pregnant (Box 3).
- **Need for back-up contraception:** No additional contraceptive protection is needed.

##### Postpartum (Including Cesarean Delivery, Breastfeeding, or Nonbreastfeeding)

- **Timing:** The Cu-IUD may be placed at any time postpartum, including immediately postpartum (U.S. MEC 1 or 2) (*1*), if it is reasonably certain that the patient is not pregnant (Box 3). Postpartum placement of IUDs is safe (*1*). Higher rates of expulsion during the postpartum period should be considered as they relate to effectiveness, along with patient access to interval placement (i.e., not related to pregnancy) when expulsion rates are lower (*1*). The Cu-IUD should not be placed in a patient with postpartum sepsis (e.g., chorioamnionitis or endometritis) (U.S. MEC 4) (*1*).
- **Need for back-up contraception**: No additional contraceptive protection is needed.

##### Postabortion (Spontaneous or Induced)

- **Timing:** The Cu-IUD may be placed at any time postabortion, including immediately after abortion completion (U.S. MEC 1 or 2) (*1*), if it is reasonably certain that the patient is not pregnant (Box 3). The Cu-IUD should not be placed immediately after a septic abortion (U.S. MEC 4) (*1*).
- **Need for back-up contraception:** No additional contraceptive protection is needed.

##### Switching from Another Contraceptive Method

- **Timing:** The Cu-IUD may be placed immediately if it is reasonably certain that the patient is not pregnant (Box 3). Waiting for the patient’s next menstrual cycle is unnecessary.
- **Need for back-up contraception:** No additional contraceptive protection is needed.

**Comments and Evidence Summary.** In situations in which the health care provider is not reasonably certain that the patient is not pregnant, the patient should be offered a contraceptive method other than an IUD to use until the health care provider can be reasonably certain that the patient is not pregnant and can place the Cu-IUD. (As appropriate, see recommendations for Emergency Contraception.)

A systematic review identified eight studies that suggested that timing of Cu-IUD placement in relation to the menstrual cycle in nonpostpartum women had little effect on long-term outcomes (i.e., rates of continuation, removal, expulsion, or pregnancy) or on short-term outcomes (i.e., pain at placement, bleeding at placement, or immediate expulsion) (*69*) (Level of evidence: II-2, fair, direct).

### Initiation of LNG-IUDs

#### Timing of LNG-IUD Placement

- The LNG-IUD may be placed at any time if it is reasonably certain that the patient is not pregnant (Box 3).

#### Need for Back-Up Contraception

- If the LNG-IUD is placed within the first 7 days since menstrual bleeding started, no additional contraceptive protection is needed.
- If the LNG-IUD is placed >7 days since menstrual bleeding started, the patient needs to abstain from sexual intercourse or use barrier methods (e.g., condoms) for the next 7 days.

#### Special Considerations

##### Amenorrhea (Not Postpartum)

- **Timing:** The LNG-IUD may be placed at any time if it is reasonably certain that the patient is not pregnant (Box 3).
- **Need for back-up contraception:** The patient needs to abstain from sexual intercourse or use barrier methods (e.g., condoms) for the next 7 days.

##### Postpartum (Including Cesarean Delivery, Breastfeeding, or Nonbreastfeeding)

- **Timing:** The LNG-IUD may be placed at any time postpartum, including immediately postpartum (U.S. MEC 1 or 2) (*1*), if it is reasonably certain that the patient is not pregnant (Box 3). Postpartum placement of IUDs is safe (*1*). Higher rates of expulsion during the postpartum period should be considered as they relate to effectiveness, along with patient access to interval placement (i.e., not related to pregnancy) when expulsion rates are lower (*1*). The LNG-IUD should not be placed in a patient with postpartum sepsis (e.g., chorioamnionitis or endometritis) (U.S. MEC 4) (*1*).
- **Need for back-up contraception:** If the patient is <6 months postpartum, amenorrheic, and fully or nearly fully breastfeeding (exclusively breastfeeding or the vast majority [≥85%] of feeds are breastfeeds) (*44*), no additional contraceptive protection is needed. Otherwise, a patient who is ≥21 days postpartum and whose menstrual cycle has not returned needs to abstain from sexual intercourse or use barrier methods (e.g., condoms) for the next 7 days. If the patient’s menstrual cycle has returned and it has been >7 days since menstrual bleeding began, the patient needs to abstain from sexual intercourse or use barrier methods (e.g., condoms) for the next 7 days.

##### Postabortion (Spontaneous or Induced)

- **Timing:** The LNG-IUD may be placed at any time postabortion, including immediately after abortion completion (U.S. MEC 1 or 2) (*1*), if it is reasonably certain that the patient is not pregnant (Box 3). The LNG-IUD should not be placed immediately after a septic abortion (U.S. MEC 4) (*1*).
- **Need for back-up contraception:** The patient needs to abstain from sexual intercourse or use barrier methods (e.g., condoms) for the next 7 days unless the IUD is placed immediately after abortion completion.

##### Switching from Another Contraceptive Method

- **Timing:** The LNG-IUD may be placed immediately if it is reasonably certain that the patient is not pregnant (Box 3). Waiting for the patient’s next menstrual cycle is unnecessary.
- **Need for back-up contraception:** If it has been >7 days since menstrual bleeding began, the patient needs to abstain from sexual intercourse or use barrier methods (e.g., condoms) for the next 7 days.
- **Switching from a Cu-IUD:** In addition to the need for back-up contraception when starting the LNG-IUD, there might be additional concerns when switching from a Cu-IUD. If the patient has had sexual intercourse since the start of their current menstrual cycle and it has been >5 days since menstrual bleeding started, theoretically, residual sperm might be in the genital tract, which could lead to fertilization if ovulation occurs. A health care provider may consider providing any type of ECP at the time of LNG-IUD placement to address the potential for residual sperm.

**Comments and Evidence Summary.** In situations in which the health care provider is uncertain whether the patient might be pregnant, the patient should be offered a contraceptive method other than an IUD to use until the health care provider can be reasonably certain that they are not pregnant and can place the LNG-IUD. If a patient needs to use additional contraceptive protection when switching to an LNG-IUD from another contraceptive method, consider continuing their previous method for 7 days after LNG-IUD placement. (As appropriate, see recommendations for Emergency Contraception.)

No direct evidence was found regarding the effects of placing LNG-IUDs on different days of the cycle on short- or long-term outcomes (*69*).

### Examinations and Tests Needed Before Initiation of a Cu-IUD or an LNG-IUD

Among healthy patients, few examinations or tests are needed before initiation of an IUD (Table 1). Bimanual examination and cervical inspection are necessary before IUD placement. A baseline weight and body mass index (BMI) measurement might be useful for addressing any concerns about changes in weight over time. If a patient has not been screened for STIs according to STI screening guidelines, screening may be performed at the time of placement. Patients with known medical problems or other special conditions might need additional examinations or tests before being determined to be appropriate candidates for a particular method of contraception. U.S. MEC might be useful in such circumstances (*1*).

**TABLE 1:** Classification of examinations and tests needed before intrauterine device initiation

Examination or test	Class*	
	Cu-IUD	LNG-IUD
**Examination**		
Blood pressure	C	C
Weight (BMI) (weight [kg]/height [m]^2^)	—^†^	—^†^
Clinical breast examination	C	C
Bimanual examination and cervical inspection	A	A
**Laboratory test**		
Glucose	C	C
Lipids	C	C
Liver enzymes	C	C
Hemoglobin	C	C
Thrombophilia	C	C
Cervical cytology (Papanicolaou smear)	C	C
STI screening with laboratory tests	—^§^	—^§^
HIV screening with laboratory tests	C	C

**Abbreviations:** BMI = body mass index; Cu-IUD = copper intrauterine device; IUD = intrauterine device; LNG-IUD = levonorgestrel intrauterine device; STI = sexually transmitted infection; U.S. MEC = *U.S. Medical Eligibility Criteria for Contraceptive Use.*

* **Class A:** Essential and mandatory in all circumstances for safe and effective use of the contraceptive method. **Class B:** Contributes substantially to safe and effective use, but implementation may be considered within the public health context, service context, or both; the risk of not performing an examination or test should be balanced against the benefits of making the contraceptive method available. **Class C:** Does not contribute substantially to safe and effective use of the contraceptive method. (**Source:** World Health Organization. Selected practice recommendations for contraceptive use, 2nd ed. Geneva, Switzerland: WHO Press; 2004.)

^†^ Weight (BMI) measurement is not needed to determine medical eligibility for any methods of contraception because all methods can be used (U.S. MEC 1) or generally can be used (U.S. MEC 2) among patients with obesity (BMI ≥30 kg/m^2^). However, measuring weight and calculating BMI at baseline might be helpful for discussing concerns about any changes in weight and whether changes might be related to use of the contraceptive method.

^§^ Most patients do not require additional STI screening at the time of IUD placement. If a patient with risk factors for STIs has not been screened for gonorrhea and chlamydia according to CDC’s *STI Treatment Guidelines* (https://www.cdc.gov/std/treatment-guidelines/default.htm), screening may be performed at the time of IUD placement, and placement should not be delayed. Patients with current purulent cervicitis or chlamydial infection or gonococcal infection should not undergo IUD placement (U.S. MEC 4).

**Comments and Evidence Summary. Weight (BMI):** Patients with obesity (BMI ≥30 kg/m^2^) can use IUDs (U.S. MEC 1) (*1*); therefore, screening for obesity is not necessary for the safe initiation of IUDs. However, measuring weight and calculating BMI (weight [kg]/height [m]^2^) at baseline might be helpful for discussing concerns about any changes in weight and whether changes might be related to use of the contraceptive method.

**Bimanual examination and cervical inspection:** Bimanual examination and cervical inspection are necessary before IUD placement to assess uterine size and position and to detect any cervical or uterine abnormalities that might indicate infection or otherwise prevent IUD placement (*70*–*73*).

**STIs:** Patients should be routinely screened for chlamydial and gonococcal infections according to national screening guidelines. The CDC *Sexually Transmitted Infections Treatment Guidelines* provide information on screening eligibility, timing, and frequency of screening and on screening for persons with risk factors (https://www.cdc.gov/std/treatment-guidelines/default.htm) (*31*). If STI screening guidelines have been followed, most patients do not need additional STI screening at the time of IUD placement, and placement should not be delayed. If a patient with risk factors for STIs has not been screened for gonorrhea and chlamydia according to CDC STI treatment guidelines, screening may be performed at the time of IUD placement, and placement should not be delayed. Patients with current purulent cervicitis or chlamydial infection or gonococcal infection should not undergo IUD placement (U.S. MEC 4) (*1*). A systematic review identified two studies that demonstrated no differences in PID rates among women who screened positive for gonorrhea or chlamydia and underwent concurrent IUD placement compared with women who screened positive and initiated other contraceptive methods (*74*). Indirect evidence demonstrates women who undergo same-day STI screening and IUD placement have similar PID rates compared with women who have delayed IUD placement. Women who undergo same-day STI screening and IUD placement have low incidence rates of PID. Algorithms for predicting PID among women with risk factors for STIs have poor predictive value. Risk for PID among women with risk factors for STIs is low (*24*,*31*,*75*–*84*). Although women with STIs at the time of IUD placement have a higher risk for PID, the overall rate of PID among all IUD users is low (*79*,*82*).

**Hemoglobin:** Patients with iron-deficiency anemia can use the LNG-IUD (U.S. MEC 1) (*1*); therefore, screening for anemia is not necessary for safe initiation of the LNG-IUD. Patients with iron-deficiency anemia generally can use Cu-IUDs (U.S. MEC 2) (*1*). Measurement of hemoglobin before initiation of Cu-IUDs is not necessary because of the minimal change in hemoglobin among patients with and without anemia using Cu-IUDs. A systematic review identified four studies that provided direct evidence for changes in hemoglobin among women with anemia who received Cu-IUDs (*85*). Evidence from one randomized trial (*86*) and one prospective cohort study (*87*) indicated no significant changes in hemoglobin among Cu-IUD users with anemia, whereas two prospective cohort studies (*88*,*89*) indicated a statistically significant decrease in hemoglobin levels during 12 months of follow-up; however, the magnitude of the decrease was small and most likely not clinically significant. The systematic review also identified 21 studies that provided indirect evidence by examining changes in hemoglobin among healthy women receiving Cu-IUDs (*90*–*110*), which generally demonstrated no clinically significant changes in hemoglobin levels with up to 5 years of follow-up (Level of evidence: I to II-2, fair, direct).

**Lipids:** Screening for dyslipidemias is not necessary for the safe initiation of Cu-IUDs or LNG-IUDs because of the low likelihood of clinically significant changes with use of hormonal contraceptives. A systematic review did not identify any evidence regarding outcomes among women who were screened versus not screened with lipid measurement before initiation of hormonal contraceptives (*24*). During 2015–2016, among women aged 20–39 years in the United States, 6.7% had high cholesterol, defined as total serum cholesterol >240 mg/dL (*111*). Studies have demonstrated mixed results about the effects of hormonal methods on lipid levels among both healthy women and women with baseline lipid abnormalities, and the clinical significance of these changes is unclear (*112*–*115*).

**Liver enzymes:** Patients with liver disease can use the Cu-IUD (U.S. MEC 1) (*1*); therefore, screening for liver disease is not necessary for the safe initiation of the Cu-IUD. Although patients with hepatocellular carcinoma generally should not use the LNG-IUD (U.S. MEC 3), patients with benign liver tumors, viral hepatitis, or cirrhosis can use (U.S. MEC 1) or generally can use (U.S. MEC 2) the LNG-IUD (*1*); screening for liver disease before initiation of the LNG-IUD is not necessary because of the low prevalence of these conditions and the high likelihood that patients with liver disease already would have had the condition diagnosed. A systematic review did not identify any evidence regarding outcomes among women who were screened versus not screened with liver enzyme tests before initiation of hormonal contraceptive use (*24*). During 2012, among U.S. women, the percentage with liver disease (not further specified) was 1.3% (*116*). During 2013, the incidence of acute hepatitis A, B, or C was ≤1 per 100,000 U.S. population (*117*). During 2002–2011, the incidence of liver cancer among U.S. women was approximately 3.7 per 100,000 population (*118*).

**Clinical breast examination:** Patients with breast disease can use the Cu-IUD (U.S. MEC 1) (*1*); therefore, screening for breast disease is not necessary for the safe initiation of the Cu-IUD. Although patients with current breast cancer should not use the LNG-IUD (U.S. MEC 4) (*1*), screening asymptomatic patients with a clinical breast examination before placing an IUD is not necessary because of the low prevalence of breast cancer among women of reproductive age. A systematic review did not identify any evidence regarding outcomes among women who were screened versus not screened with a breast examination before initiation of hormonal contraceptives (*23*). The incidence of breast cancer among women of reproductive age in the United States is low. During 2020, the incidence of breast cancer among women aged <50 years was approximately 45.9 per 100,000 women (*119*).

**Cervical cytology:** Although patients with cervical cancer should not undergo IUD placement (U.S. MEC 4) (*1*), screening asymptomatic patients with cervical cytology before IUD placement is not necessary because of the high rates of cervical screening, low incidence of cervical cancer in the United States, and high likelihood that a patient with cervical cancer already would have had the condition diagnosed. A systematic review did not identify any evidence regarding outcomes among women who were screened versus not screened with cervical cytology before initiation of IUDs (*24*). Cervical cancer is rare in the United States, with an incidence rate of 9.8 per 100,000 women during 2012 (*119*). The incidence and mortality rates from cervical cancer have declined dramatically in the United States, largely because of cervical cytology screening (*120*). Overall screening rates for cervical cancer in the United States are high; during 2013 among women aged 18–44 years, approximately 77% reported having cervical cytology screening within the past 3 years (*121*).

**HIV screening:** Patients with HIV infection can use (U.S. MEC 1) or generally can use (U.S. MEC 2) IUDs, depending on whether they are clinically well and receiving antiretroviral therapy (*1*). Therefore, HIV screening is not necessary before IUD placement. A systematic review did not identify any evidence regarding outcomes among women who were screened versus not screened for HIV infection before IUD placement (*24*). Limited evidence suggests that IUDs are not associated with disease progression, increased infection, or other adverse health effects among women with HIV infection (*122*–*137*).

**Other screening:** Patients with hypertension, diabetes, or thrombophilia can use (U.S. MEC 1) or generally can use (U.S. MEC 2) IUDs (*1*). Therefore, screening for these conditions is not necessary for the safe initiation of IUDs.

### Provision of Medications for IUD Placement

- Misoprostol is not recommended for routine use for IUD placement. Misoprostol might be useful in selected circumstances (e.g., in patients with a recent failed placement).
- Lidocaine (paracervical block or topical) for IUD placement might be useful for reducing patient pain.

**Comments and Evidence Summary.** Before IUD placement, all patients should be counseled on potential pain during placement as well as the risks, benefits, and alternatives of different options for pain management. A person-centered plan for IUD placement and pain management should be made based on patient preference. Barriers to IUD use include patient concerns about anticipated pain with placement and provider concerns about ease of placement, especially among nulliparous patients (*138*–*140*). When considering patient pain, it is important to recognize that the experience of pain is individualized and might be influenced by previous experiences including trauma and mental health conditions, such as depression or anxiety (*141*–*143*). Although these recommendations for provision of medications for IUD placement are based on the best available evidence, not all populations or patient experiences are represented in the literature. The following evidence summary represents results from a systematic review and meta-analysis (Supplementary Appendix, https://stacks.cdc.gov/view/cdc/156517) and focuses on findings that were statistically significant and clinically relevant.

**Misoprostol:** Evidence includes 14 RCTs (Supplementary Appendix, https://stacks.cdc.gov/view/cdc/156517). Eleven trials examined 400 *µ*g doses and three trials examined <400 *µ*g doses. The route of administration varied across trials and included vaginal, buccal, sublingual, and oral administration. For patients without a recent failed IUD placement attempt, the timing of administration ranged from 1 to 8 hours before IUD placement.

- Evidence suggests that misoprostol does not reduce patient pain, adverse events, or need for adjunctive placement measures (e.g., cervical dilation), nor improve provider ease of placement, placement success, or patient satisfaction with the procedure (Supplementary Appendix, https://stacks.cdc.gov/view/cdc/156517).
- Evidence suggests that misoprostol might increase patient pain and preplacement abdominal pain or cramping and diarrhea but is not associated with other side effects (i.e., nausea or vomiting) (Supplementary Appendix, https://stacks.cdc.gov/view/cdc/156517).
- Evidence from one trial among women with a recent failed IUD placement suggests that pretreatment with 400 *µ*g vaginal misoprostol (200 *µ*g administered 10 hours before and 200 *µ*g administered 4 hours before returning to the clinic for a subsequent IUD placement attempt) might result in higher placement success with second placement attempt compared with placebo (Supplementary Appendix, https://stacks.cdc.gov/view/cdc/156517).
- Certainty of evidence: moderate for patient pain, need for adjunctive placement measures, placement success for patients with and without recent prior failed placement attempt, side effects, and patient satisfaction with the procedure; low for provider ease of placement and adverse events.

**Lidocaine as a paracervical block:** Evidence for lidocaine as a paracervical block includes six RCTs (Supplementary Appendix, https://stacks.cdc.gov/view/cdc/156517). Four trials examined 1% lidocaine paracervical block (10–20 mL), and two examined 2% lidocaine paracervical block (10–12 mL). The timing of block administration ranged from just before to at least 5 minutes before IUD placement. All six trials administered 2-point injections, and four also administered a tenaculum site injection.

- Evidence suggests that lidocaine as a paracervical block might reduce patient pain (Supplementary Appendix, https://stacks.cdc.gov/view/cdc/156517).
  º Three RCTs found reductions in pain at either tenaculum placement, during IUD placement, or after IUD placement before clinic discharge among patients receiving either paracervical block with 1% lidocaine just before to 3 minutes before IUD placement or paracervical block with 2% lidocaine at least 5 minutes before IUD placement compared with patients receiving no treatment or placebo/sham block. However, evidence from three additional RCTs, examined individually or in meta-analysis, did not suggest a reduction in patient pain or did not include statistical testing between groups of interest (Supplementary Appendix, https://stacks.cdc.gov/view/cdc/156517).
- Evidence suggests that lidocaine as a paracervical block does not reduce adverse events or need for adjunctive placement measures (e.g., cervical dilation), increase side effects (specifically tinnitus, vomiting, or dizziness), nor improve placement success or patient satisfaction with the procedure (Supplementary Appendix, https://stacks.cdc.gov/view/cdc/156517).
- No evidence on provider ease of placement was found.
- Certainty of evidence: moderate for side effects; low for patient pain, need for adjunctive placement measures, placement success, and patient satisfaction with the procedure; very low for adverse events.

**Lidocaine as a topical gel, cream, or spray:** Evidence for lidocaine as a topical gel, cream, or spray includes 13 RCTs (Supplementary Appendix, https://stacks.cdc.gov/view/cdc/156517). Five trials examined 2% lidocaine topical gel (two intracervical, one cervical, and two vaginal), one examined 10% lidocaine topical spray (intracervical) and lidocaine topical cream (intracervical), three examined 10% lidocaine topical spray (cervical), three examined lidocaine-prilocaine cream (cervical), and one examined 2% lidocaine topical gel (cervical) plus oral diclofenac. The topical lidocaine was administered by a provider (1–7 minutes before IUD placement) in 11 trials and self-administered by patients (at least 15 minutes before IUD placement) in two trials.

- Evidence suggests that topical lidocaine might reduce patient pain (Supplementary Appendix, https://stacks.cdc.gov/view/cdc/156517).
  º One meta-analysis of four RCTs found that topical lidocaine was associated with reduced pain during tenaculum placement. In addition, two RCTs found reduced pain at either tenaculum placement, during IUD placement, or after IUD placement before clinic discharge among patients self-administering 2% lidocaine topical gel (vaginal) 5–15 minutes before IUD placement or those receiving provider-administered lidocaine-prilocaine topical cream (cervical) 7 minutes before IUD placement. However, evidence from seven additional trials, examined individually or in meta-analysis, did not suggest a reduction in patient pain.
- Evidence suggests that topical lidocaine does not reduce adverse events or the need for adjunctive placement measures (e.g., cervical dilation), nor improve placement success, patient satisfaction with the procedure, nor improve provider ease of placement (Supplementary Appendix, https://stacks.cdc.gov/view/cdc/156517).
- No evidence on side effects was found.
- Certainty of evidence: high for placement success; moderate for provider ease of placement, patient pain, need for adjunctive placement measures, and patient satisfaction with the procedure; low for adverse events.

**Additional interventions for which evidence suggested no positive effect or evidence was too limited to make a recommendation:** Evidence on multiple other interventions was identified, including lidocaine as an intracervical block (one trial), intrauterine instillation (four trials), analgesics (17 trials on seven different interventions), smooth muscle relaxants (six trials on five different interventions), and dinoprostone (five trials) (*144*). For these interventions, the evidence either suggested no positive effect on the outcomes assessed or the evidence was too limited to make a recommendation. A detailed summary of the evidence is provided (Supplementary Appendix, https://stacks.cdc.gov/view/cdc/156517).

### Provision of Prophylactic Antibiotics at the Time of IUD Placement

- Prophylactic antibiotics are generally not recommended for Cu-IUD or LNG-IUD placement.

**Comments and Evidence Summary.** Theoretically, IUD placement could induce bacterial spread and lead to complications such as PID or infective endocarditis. A meta-analysis was conducted of RCTs examining antibiotic prophylaxis versus placebo or no treatment for IUD placement (*145*). Use of prophylaxis reduced the frequency of unscheduled return visits but did not significantly reduce the incidence of PID or IUD discontinuation. Although the risk for PID was higher within the first 20 days after placement, the incidence of PID was low among all women who had IUDs placed (*79*). According to the American Heart Association, administration of prophylactic antibiotics solely to prevent endocarditis is not recommended for patients who undergo genitourinary tract procedures, including insertion or removal of IUDs (*146*). Studies have not demonstrated a conclusive link between genitourinary procedures and infective endocarditis or a preventive benefit of prophylactic antibiotics during such procedures (*146*).

### Routine Follow-Up After IUD Placement

These recommendations address when routine follow-up is needed for safe and effective continued use of contraception for healthy patients. The recommendations refer to general situations and might vary for different users and different situations. Specific populations who might benefit from more frequent follow-up visits include adolescents, persons with certain medical conditions or characteristics, and persons with multiple medical conditions.

- Advise the patient that they may contact their provider at any time to discuss side effects or other problems, if they want to change the method being used, and when it is time to remove or replace the contraceptive method. No routine follow-up visit is required.
- At other routine visits, health care providers who see IUD users should do the following:
  º Assess the patient’s satisfaction with their contraceptive method and whether they have any concerns about method use.
  º Assess any changes in health status, including medications, that would change the appropriateness of the IUD for safe and effective continued use on the basis of U.S. MEC (e.g., category 3 and 4 conditions and characteristics) (*1*).
  º Consider performing an examination to check for the presence of the IUD strings.
  º Consider assessing weight changes and discussing concerns about any changes in weight and whether changes might be related to use of the contraceptive method.

**Comments and Evidence Summary.** Evidence from a systematic review about the effect of a specific follow-up visit schedule on IUD continuation is very limited and of poor quality. The evidence did not suggest that greater frequency of visits or earlier timing of the first follow-up visit after placement improves continuation of use (*22*) (Level of evidence: II-2, poor, direct). Evidence from four studies from a systematic review on the incidence of PID among IUD initiators, or IUD removal as a result of PID, suggested that the incidence of PID did not differ between women using Cu-IUDs and those using DMPA, COCs, or LNG-IUDs (*21*) (Level of evidence: I to II-2, good, indirect). Evidence on the timing of PID after IUD placement is mixed. Although the rate of PID generally was low, the largest study suggested that the rate of PID was significantly higher in the first 20 days after placement (*79*) (Level of evidence: I to II-3, good to poor, indirect).

### Bleeding Irregularities with Cu-IUD Use

- Before Cu-IUD placement, provide counseling about potential changes in bleeding patterns during Cu-IUD use. Spotting or light bleeding and heavy or prolonged bleeding is common during the first 3–6 months of Cu-IUD use, is generally not harmful but might be bothersome to the patient, and generally decreases with continued Cu-IUD use.
- If clinically indicated, consider an underlying health condition, such as Cu-IUD displacement, STIs, pregnancy, thyroid disorders, or new pathologic uterine conditions (e.g., polyps or fibroids), especially in patients who have already been using the Cu-IUD for a few months or longer and who have developed a new onset of heavy or prolonged bleeding. If an underlying health condition is found, treat the condition or refer for care.
- Explore patient goals, including continued Cu-IUD use (with or without treatment for bleeding irregularities) or Cu-IUD removal. If the patient wants to continue Cu-IUD use, provide reassurance, discuss options for management of bleeding irregularities if it is desired, and advise the patient that they may contact their provider at any time to discuss bleeding irregularities or other side effects.
- If the patient desires Cu-IUD removal at any time, remove the Cu-IUD, offer counseling on alternative contraceptive methods, and initiate another method if it is desired.
- If the patient wants treatment, the following treatment option may be considered during days of bleeding, depending on the patient’s preferences, treatment goals, and medical history:
  º NSAIDs for short-term treatment, 5–7 days

**Comments and Evidence Summary.** During contraceptive counseling and before placement of the Cu-IUD, information about common side effects such as spotting or light bleeding or heavy or prolonged menstrual bleeding, especially during the first 3–6 months of use, should be discussed (*91*). These bleeding irregularities are generally not harmful but might be bothersome to the patient. Enhanced counseling about expected bleeding patterns and reassurance that bleeding irregularities are generally not harmful has been reported to reduce method discontinuation in clinical trials with other contraceptives (i.e., DMPA) (*147*,*148*).

Evidence is limited on specific drugs, doses, and durations of use for effective treatments for bleeding irregularities with Cu-IUD use. Therefore, this report includes general recommendations for treatments to consider rather than specific regimens.

A systematic review identified 11 studies that examined various therapeutic treatments for heavy menstrual bleeding, prolonged menstrual bleeding, or both among women using Cu-IUDs (*149*). Nine studies examined the use of various oral NSAIDs for the treatment of heavy or prolonged menstrual bleeding among Cu-IUD users and compared them with either a placebo or a baseline cycle. Three of these trials examined the use of indomethacin (*150*–*152*), three examined mefenamic acid (*153*–*155*), and three examined flufenamic acid (*150*,*151*,*156*). Other NSAIDs used in the reported trials included alclofenac (*150*,*151*), suprofen (*157*), and diclofenac sodium (*158*). All but one NSAID study (*154*) demonstrated statistically significant or notable reductions in mean total menstrual blood loss with NSAID use. One study among 19 Cu-IUD users with heavy bleeding suggested that treatment with oral tranexamic acid can significantly reduce mean blood loss during treatment compared with placebo (*158*). Data regarding the overall safety of tranexamic acid are limited; an FDA warning states that tranexamic acid is contraindicated in women with active thromboembolic disease or with a history or intrinsic risk for thrombosis or thromboembolism (*159*,*160*). Treatment with aspirin demonstrated no statistically significant change in mean blood loss among women whose pretreatment menstrual blood loss was >80 mL or 60–80 mL; treatment resulted in a significant increase among women whose pretreatment menstrual blood loss was <60 mL (*161*). One study examined the use of a synthetic form of vasopressin, intranasal desmopressin (300 *µ*g/day) for the first 5 days of menses for three treatment cycles and found a significant reduction in mean blood loss compared with baseline (*153*) (Level of evidence: I to II-3, poor to fair, direct). Only one small study examined treatment of spotting with three separate NSAIDs and did not observe improvements in spotting in any of the groups (*150*) (Level of evidence: I, poor, direct).

### Bleeding Irregularities (Including Amenorrhea) with LNG-IUD Use

- Before LNG-IUD placement, provide counseling about potential changes in bleeding patterns during LNG-IUD use. Spotting or light bleeding is expected during the first 3–6 months of LNG-IUD use and is generally not harmful but might be bothersome to the patient. Over time, bleeding generally decreases with LNG-IUD use, and many LNG-IUD users experience only light menstrual bleeding or amenorrhea. Heavy or prolonged bleeding is uncommon during LNG-IUD use.

#### Bleeding Irregularities (Spotting, Light Bleeding, or Heavy or Prolonged Bleeding)

- If clinically indicated, consider an underlying health condition, such as LNG-IUD displacement, STIs, pregnancy, thyroid disorders, or new pathologic uterine conditions (e.g., polyps or fibroids). If an underlying health condition is found, treat the condition or refer for care.
- Explore patient goals, including continued LNG-IUD use or LNG-IUD removal. If the patient wants to continue LNG-IUD use, provide reassurance and advise the patient that they may contact their provider at any time to discuss bleeding irregularities or other side effects.
- If the patient desires LNG-IUD removal at any time, remove the LNG-IUD, offer counseling on alternative contraceptive methods, and initiate another method if it is desired.

#### Amenorrhea

- Amenorrhea does not require any medical treatment. Provide reassurance.
  º If a patient’s regular bleeding pattern changes abruptly to amenorrhea, consider ruling out pregnancy if clinically indicated.
- If the patient desires LNG-IUD removal, remove the LNG-IUD, offer counseling on alternative contraceptive methods, and initiate another method if it is desired.

**Comments and Evidence Summary.** During contraceptive counseling and before placement of the LNG-IUD, information about common side effects such as spotting or light bleeding, especially during the first 3–6 months of use, should be discussed. Approximately half of LNG-IUD users are likely to experience amenorrhea or oligomenorrhea by 2 years of use (*162*). These bleeding irregularities are generally not harmful but might be bothersome to the patient. Enhanced counseling about expected bleeding patterns and reassurance that bleeding irregularities are generally not harmful has been reported to reduce method discontinuation in clinical trials with other hormonal contraceptives (i.e., DMPA) (*147*,*148*).

A systematic review summarized the current body of evidence for treating bleeding irregularities with 52 mg LNG-IUD use (*163*) (Supplementary Appendix, https://stacks.cdc.gov/view/cdc/156517). RCTs of tranexamic acid (*164*), mefenamic acid (*164*), and UPA (*165*) for the treatment of bleeding irregularities with 52 mg LNG-IUDs observed no differences between the treatment and placebo groups in bleeding or spotting over 90 days. A prospective cohort study examining oral estradiol demonstrated a significant reduction in bleeding days after 3 months of treatment compared with baseline; however, 68% of patients experienced side effects (e.g., painful or swollen breasts, headache, weight gain, and vaginal complaints) (*166*) (Certainty of evidence: moderate to high for RCTs and very low for the observational study).

### Management of the IUD when a Cu-IUD or an LNG-IUD User Is Found To Have PID

- Treat the PID according to the CDC *Sexually Transmitted Infections Treatment Guidelines* (https://www.cdc.gov/std/treatment-guidelines/default.htm) (*31**)*.
- Provide comprehensive management for STIs, including counseling about condom use.
- The IUD does not need to be removed immediately if the patient needs ongoing contraception.
- Reassess the patient in 48–72 hours. If no clinical improvement occurs, continue antibiotics and consider removal of the IUD.
- If the patient wants to discontinue use, remove the IUD sometime after antibiotics have been started to avoid the potential risk for bacterial spread resulting from the removal procedure.
- If the IUD is removed, consider ECPs if appropriate. Counsel the patient on alternative contraceptive methods and offer another method if it is desired.

**Comments and Evidence Summary.** Treatment outcomes do not generally differ between patients with PID who retain the IUD and those who have the IUD removed; however, appropriate antibiotic treatment and close clinical follow-up are necessary. A summary of IUD management in patients with PID is provided (Appendix F).

A systematic review identified four studies that included women using Cu-IUDs or other nonhormonal IUDs who developed PID and compared outcomes between women who had the IUD removed and those who did not (*167*). One RCT demonstrated that women with IUDs removed had longer hospitalizations than those who did not, although no differences in PID recurrences or subsequent pregnancies were observed (*168*). Another RCT demonstrated no differences in laboratory findings among women who removed the IUD compared with those who did not (*169*). One prospective cohort study reported no differences in clinical or laboratory findings during hospitalization; however, the IUD removal group had longer hospitalizations (*170*). One RCT illustrated that the rate of recovery for most clinical signs and symptoms was higher among women who had the IUD removed than among women who did not (*171*). No evidence was found regarding women using LNG-IUDs (Level of evidence: I to II-2, fair, direct).

### Management of the IUD when a Cu-IUD or an LNG-IUD User Is Found To Be Pregnant

- Evaluate for possible ectopic pregnancy.
- Advise the patient that they have an increased risk for spontaneous abortion (including septic abortion that might be life threatening) and for preterm delivery if the IUD is left in place. The removal of the IUD reduces these risks but might not decrease the risk to the baseline level of a pregnancy without an IUD.
  º If the patient does not want to continue the pregnancy, counsel them about options.
  º If the patient wants to continue the pregnancy, advise them to seek care promptly if they have heavy bleeding, cramping, pain, abnormal vaginal discharge, or fever.

#### IUD Strings Are Visible or Can Be Retrieved Safely from the Cervical Canal

- Advise the patient that the IUD should be removed as soon as possible.
  º If the IUD is to be removed, remove it by pulling on the strings gently.
  º Advise the patient that they should return promptly if they have heavy bleeding, cramping, pain, abnormal vaginal discharge, or fever.
- If the patient chooses to keep the IUD, advise them to seek care promptly if they have heavy bleeding, cramping, pain, abnormal vaginal discharge, or fever.

#### IUD Strings Are Not Visible and Cannot Be Safely Retrieved

- If ultrasonography is available, consider performing or referring for ultrasound examination to determine the location of the IUD. If the IUD cannot be located, it might have been expelled or have perforated the uterine wall.
- If ultrasonography is not possible or the IUD is determined by ultrasound to be inside the uterus, advise the patient to seek care promptly if they have heavy bleeding, cramping, pain, abnormal vaginal discharge, or fever.

**Comments and Evidence Summary.** Removing the IUD improves the pregnancy outcome if the IUD strings are visible or the device can be retrieved safely from the cervical canal. Risks for spontaneous abortion, preterm delivery, and infection are substantial if the IUD is left in place.

Theoretically, the fetus might be affected by hormonal exposure from an LNG-IUD. However, whether this exposure increases the risk for fetal abnormalities is unknown.

A systematic review identified nine studies suggesting that women who did not remove their IUDs during pregnancy were at greater risk for adverse pregnancy outcomes (e.g., spontaneous abortion, septic abortion, preterm delivery, and chorioamnionitis) compared with women who had their IUDs removed or who did not have an IUD (*58*). Cu-IUD removal decreased risks but not to the baseline risk for pregnancies without an IUD. One case series examined LNG-IUDs. When the IUDs were not removed, eight out of 10 pregnancies ended in spontaneous abortions (Level of evidence: II-2, fair, direct).

## Implants

The ENG implant, a single rod with 68 mg of ENG, is available in the United States. Fewer than one implant user out of 100 become pregnant in the first year with typical use (*28*). The implant is long acting, is reversible, and can be used by patients of all ages, including adolescents. The implant does not protect against STIs, including HIV infection, and patients using the implant should be counseled that consistent and correct use of external (male) latex condoms reduces the risk for STIs, including HIV infection (*31*). Use of internal (female) condoms can provide protection from STIs, including HIV infection, although data are limited (*31*). Patients also should be counseled that PrEP, when taken as prescribed, is highly effective for preventing HIV infection (*32*).

### Initiation of Implants

#### Timing

- The implant may be placed at any time if it is reasonably certain that the patient is not pregnant (Box 3).

#### Need for Back-Up Contraception

- If the implant is placed within the first 5 days since menstrual bleeding started, no additional contraceptive protection is needed.
- If the implant is placed >5 days since menstrual bleeding started, the patient needs to abstain from sexual intercourse or use barrier methods (e.g., condoms) for the next 7 days.

#### Special Considerations

##### Amenorrhea (Not Postpartum)

- **Timing:** The implant may be placed at any time if it is reasonably certain that the patient is not pregnant (Box 3).
- **Need for back-up contraception:** The patient needs to abstain from sexual intercourse or use barrier methods (e.g., condoms) for the next 7 days.

##### Postpartum (Breastfeeding)

- **Timing:** The implant may be placed at any time (U.S. MEC 2 if <30 days postpartum and U.S. MEC 1 if ≥30 days postpartum) (*1*), if it is reasonably certain that the patient is not pregnant (Box 3).
- **Need for back-up contraception:** If the patient is <6 months postpartum, amenorrheic, and fully or nearly fully breastfeeding (exclusively breastfeeding or the vast majority [≥85%] of feeds are breastfeeds) (*44*), no additional contraceptive protection is needed. Otherwise, a patient who is ≥21 days postpartum and whose menstrual cycle has not returned needs to abstain from sexual intercourse or use barrier methods (e.g., condoms) for the next 7 days. If the patient’s menstrual cycle has returned and it has been >5 days since menstrual bleeding started, the patient needs to abstain from sexual intercourse or use barrier methods (e.g., condoms) for the next 7 days.

##### Postpartum (Nonbreastfeeding)

- **Timing:** The implant may be placed at any time, including immediately postpartum (U.S. MEC 1) (*1*), if it is reasonably certain that the patient is not pregnant (Box 3).
- **Need for back-up contraception:** If the patient is <21 days postpartum, no additional contraceptive protection is needed. A patient who is ≥21 days postpartum and whose menstrual cycle has not returned needs to abstain from sexual intercourse or use barrier methods (e.g., condoms) for the next 7 days. If the patient’s menstrual cycle has returned and it has been >5 days since menstrual bleeding started, the patient needs to abstain from sexual intercourse or use barrier methods (e.g., condoms) for the next 7 days.

##### Postabortion (Spontaneous or Induced)

- **Timing:** The implant may be placed at any time postabortion, including immediately after abortion completion, if it is reasonably certain that the patient is not pregnant (Box 3), or at time of medication abortion initiation (U.S. MEC 1) (*1*).
- **Need for back-up contraception:** The patient needs to abstain from sexual intercourse or use barrier methods (e.g., condoms) for the next 7 days unless the implant is placed at the time of an abortion.

##### Switching from Another Contraceptive Method

- **Timing:** The implant may be placed immediately if it is reasonably certain that the patient is not pregnant (Box 3). Waiting for the patient’s next menstrual cycle is unnecessary.
- **Need for back-up contraception:** If it has been >5 days since menstrual bleeding started, the patient needs to abstain from sexual intercourse or use barrier methods (e.g., condoms) for the next 7 days.
- **Switching from an IUD:** In addition to the need for back-up contraception when starting the implant, there might be additional concerns when switching from an IUD. If the patient has had sexual intercourse since the start of their current menstrual cycle and it has been >5 days since menstrual bleeding started, theoretically, residual sperm might be in the genital tract, which could lead to fertilization if ovulation occurs. A health care provider may consider any of the following options to address the potential for residual sperm:
  º Advise the patient to retain the IUD for at least 7 days after the implant is placed and return for IUD removal.
  º Advise the patient to abstain from sexual intercourse or use barrier methods (e.g., condoms) for 7 days before removing the IUD and switching to the new method. If it has been >5 days since menstrual bleeding started, the patient needs to abstain from sexual intercourse or use barrier methods (e.g., condoms) for the next 7 days.
  º If the patient cannot return for IUD removal and has not abstained from sexual intercourse or used barrier methods (e.g., condoms) for 7 days, advise the patient to use ECPs (with the exception of UPA) at the time of IUD removal. If it has been >5 days since menstrual bleeding started, the patient needs to abstain from sexual intercourse or use barrier methods (e.g., condoms) for the next 7 days.

**Comments and Evidence Summary.** In situations in which the health care provider is uncertain whether the patient might be pregnant, the benefits of starting the implant likely exceed any risk. Therefore, starting the implant should be considered at any time, with a follow-up pregnancy test in 2–4 weeks. If a patient needs to use additional contraceptive protection when switching to an implant from another contraceptive method, consider continuing their previous method for 7 days after implant placement. (As appropriate, see recommendations for Emergency Contraception.)

No direct evidence was found regarding the effects of starting the ENG implant at different times of the cycle.

### Examinations and Tests Needed Before Implant Initiation

Among healthy patients, no examinations or tests are needed before initiation of an implant, although a baseline weight and BMI measurement might be useful for addressing any concerns about changes in weight over time (Table 2). Patients with known medical problems or other special conditions might need additional examinations or tests before being determined to be appropriate candidates for a particular method of contraception. U.S. MEC might be useful in such circumstances (*1*).

**TABLE 2:** Classification of examinations and tests needed before implant initiation

Examination or test	Class*
**Examination**	
Blood pressure	C
Weight (BMI) (weight [kg]/height [m]^2^)	—^†^
Clinical breast examination	C
Bimanual examination and cervical inspection	C
**Laboratory test**	
Glucose	C
Lipids	C
Liver enzymes	C
Hemoglobin	C
Thrombophilia	C
Cervical cytology (Papanicolaou smear)	C
STI screening with laboratory tests	C
HIV screening with laboratory tests	C

**Abbreviations:** BMI = body mass index; STI = sexually transmitted infection; U.S. MEC = *U.S. Medical Eligibility Criteria for Contraceptive Use.*

* Class A: Essential and mandatory in all circumstances for safe and effective use of the contraceptive method. **Class B:** Contributes substantially to safe and effective use, but implementation may be considered within the public health context, service context, or both; the risk of not performing an examination or test should be balanced against the benefits of making the contraceptive method available. **Class C:** Does not contribute substantially to safe and effective use of the contraceptive method. (**Source:** World Health Organization. Selected practice recommendations for contraceptive use, 2nd ed. Geneva, Switzerland: WHO Press; 2004.)

^†^ Weight (BMI) measurement is not needed to determine medical eligibility for any methods of contraception because all methods can be used (U.S. MEC 1) or generally can be used (U.S. MEC 2) among patients with obesity (BMI ≥30 kg/m^2^). However, measuring weight and calculating BMI at baseline might be helpful for discussing concerns about any changes in weight and whether changes might be related to use of the contraceptive method.

**Comments and Evidence Summary. Weight (BMI):** Patients with obesity (BMI ≥30 kg/m^2^) can use implants (U.S. MEC 1) (*1*); therefore, screening for obesity is not necessary for the safe initiation of implants. However, measuring weight and calculating BMI at baseline might be helpful for discussing concerns about any changes in weight and whether changes might be related to use of the contraceptive method.

**Bimanual examination and cervical inspection:** A pelvic examination is not necessary before initiation of implants because it would not facilitate detection of conditions for which implant use would be unsafe. Although patients with certain conditions or characteristics should not use (U.S. MEC 4) or generally should not use (U.S. MEC 3) implants (*1*), none of these conditions are likely to be detected by pelvic examination (*172*). A systematic review identified two case-control studies that compared delayed and immediate pelvic examination before initiation of hormonal contraceptives, specifically oral contraceptives or DMPA (*23*). No differences in risk factors for cervical neoplasia, incidence of STIs, incidence of abnormal Papanicolaou smears, or incidence of abnormal wet mounts were observed. No evidence was found regarding implants (Level of evidence: II-2 fair, direct).

**Lipids:** Screening for dyslipidemias is not necessary for the safe initiation of implants because of the low likelihood of clinically significant changes with use of hormonal contraceptives. A systematic review did not identify any evidence regarding outcomes among women who were screened versus not screened with lipid measurement before initiation of hormonal contraceptives (*24*). During 2015–2016, among women aged 20–39 years in the United States, 6.7% had high cholesterol, defined as total serum cholesterol >240 mg/dL (*111*). Studies have reported mixed results regarding the effects of hormonal methods on lipid levels among both healthy women and women with baseline lipid abnormalities, and the clinical significance of these changes is unclear (*112*–*115*).

**Liver enzymes:** Although patients with hepatocellular carcinoma generally should not use implants (U.S. MEC 3) (*1*), patients with benign liver tumors, viral hepatitis, or cirrhosis can use (U.S. MEC 1) or generally can use (U.S. MEC 2) implants (*1*); screening for liver disease before initiation of implants is not necessary because of the low prevalence of these conditions and the high likelihood that patients with liver disease already would have had the condition diagnosed. A systematic review did not identify any evidence regarding outcomes among women who were screened versus not screened with liver enzyme tests before initiation of hormonal contraceptives (*24*). During 2012, the percentage of U.S. women with liver disease (not further specified) was 1.3% (*116*). During 2013, the incidence of acute hepatitis A, B, or C was ≤1 per 100,000 U.S. population (*117*). During 2002–2011, the incidence of liver cancer among U.S. women was approximately 3.7 per 100,000 population (*118*).

**Clinical breast examination:** Although patients with current breast cancer should not use implants (U.S. MEC 4) (*1*), screening asymptomatic patients with a clinical breast examination before initiation of implants is not necessary because of the low prevalence of breast cancer among women of reproductive age (15–49 years). A systematic review did not identify any evidence regarding outcomes among women who were screened versus not screened with a breast examination before initiation of hormonal contraceptives (*23*). The incidence of breast cancer among women of reproductive age in the United States is low. During 2020, the incidence of breast cancer among women aged <50 years was approximately 45.9 per 100,000 women (*119*).

**Other screening:** Patients with hypertension, diabetes, iron-deficiency anemia, thrombophilia, cervical intraepithelial neoplasia, cervical cancer, STIs, or HIV infection can use (U.S. MEC 1) or generally can use (U.S. MEC 2) implants (*1*). Therefore, screening for these conditions is not necessary for the safe initiation of implants.

### Routine Follow-Up After Implant Placement

These recommendations address when routine follow-up is needed for safe and effective continued use of contraception for healthy patients. The recommendations refer to general situations and might vary for different users and different situations. Specific populations who might benefit from more frequent follow-up visits include adolescents, those with certain medical conditions or characteristics, and those with multiple medical conditions.

- Advise the patient that they may contact their provider at any time to discuss side effects or other problems, if they want to change the method being used, and when it is time to remove or replace the contraceptive method. No routine follow-up visit is required.
- At other routine visits, health care providers seeing implant users should do the following:
  º Assess the patient’s satisfaction with their contraceptive method and whether they have any concerns about method use.
  º Assess any changes in health status, including medications, that would change the appropriateness of the implant for safe and effective continued use on the basis of U.S. MEC (e.g., category 3 and 4 conditions and characteristics) (*1*).
  º Consider assessing weight changes and discussing concerns about any changes in weight and whether changes might be related to use of the contraceptive method.

**Comments and Evidence Summary.** A systematic review did not identify any evidence regarding whether a routine follow-up visit after initiating an implant improves correct or continued use (*22*).

### Bleeding Irregularities (Including Amenorrhea) During Implant Use

- Before implant placement, provide counseling about potential changes in bleeding patterns during implant use. Spotting or light bleeding is common with implant use, and certain implant users experience amenorrhea. These bleeding changes are generally not harmful but might be bothersome to the patient. Bleeding changes might or might not decrease with continued implant use. Heavy bleeding is uncommon during implant use.

#### Bleeding Irregularities (Spotting, Light Bleeding, or Heavy or Prolonged Bleeding)

- If clinically indicated, consider an underlying health condition, such as interactions with other medications, STIs, pregnancy, thyroid disorders, or new pathologic uterine conditions (e.g., polyps or fibroids). If an underlying health condition is found, treat the condition or refer for care.
- Explore patient goals, including continued implant use (with or without treatment for bleeding irregularities) or implant removal. If the patient wants to continue implant use, provide reassurance, discuss options for management of bleeding irregularities if it is desired, and advise the patient that they may contact their provider at any time to discuss bleeding irregularities or other side effects.
- If the patient desires implant removal at any time, remove the implant, offer counseling on alternative contraceptive methods, and initiate another method if it is desired.
- If the patient wants treatment, the following treatment options may be considered, depending on the patient’s preferences, treatment goals, and medical history:
  º Treatments that might improve bleeding irregularities during treatment use; bleeding is likely to recur after treatment cessation. Treatment may be repeated as needed.
    - Hormonal treatment (e.g., 20–30 *µ*g EE COCs or estrogen)
    - Antifibrinolytic agents (e.g., tranexamic acid), 5 days
  º Treatments that might improve bleeding irregularities during treatment use and whose effects might persist for some time after treatment cessation. Treatment may be repeated as needed.
    - NSAIDs (e.g., celecoxib, ibuprofen, or mefenamic acid), 5–7 days
    - SERMs (e.g., tamoxifen), 7–10 days

#### Amenorrhea

- Amenorrhea does not require any medical treatment. Provide reassurance.
  º If a patient’s regular bleeding pattern changes abruptly to amenorrhea, consider ruling out pregnancy if clinically indicated.
- If the patient desires implant removal, remove the implant, offer counseling on alternative contraceptive methods, and initiate another method if it is desired.

**Comments and Evidence Summary.** During contraceptive counseling and before placement of the implant, information about common side effects, such as spotting or light bleeding and amenorrhea, especially during the first year of use, should be discussed. A pooled analysis of data from 11 clinical trials indicates that a significant proportion of ENG implant users had relatively little bleeding: 22% of women experienced amenorrhea and 34% experienced infrequent spotting, although 7% reported frequent bleeding and 18% reported prolonged bleeding (*173*). Bleeding or amenorrhea is generally not harmful but might be bothersome to the patient. Enhanced counseling about expected bleeding patterns and reassurance that bleeding irregularities are generally not harmful has been demonstrated to reduce method discontinuation in clinical trials with other hormonal contraceptives (i.e., DMPA) (*147*,*148*).

For patients seeking care for bleeding irregularities while using an implant, it is important to explore patient goals, including removal of the implant, treatment for bleeding irregularities, or continued use of the implant without treatment. Irregular bleeding during contraceptive implant use might be caused by several mechanisms, including an atrophic endometrium, dysregulated angiogenesis, increased matrix metalloproteinase activity, or increased expression of prostaglandin metabolites (*174**–**178*). Multiple treatments have been evaluated to manage irregular bleeding with implant use, which have different proposed mechanisms of action and likely different effects.

- NSAIDs decrease prostaglandin levels and might reduce menstrual blood loss (*179*).
- Estrogen alone or estrogen-containing contraception has been used to help stabilize the endometrium and was initially proposed as bleeding episodes in LNG implant users were associated with low serum estradiol levels (*180*,*181*).
- SERMs and selective progesterone receptor modulators (SPRMs) (e.g., tamoxifen, mifepristone, and UPA) might modulate endometrial angiogenesis and endometrial proliferation (*175*,*182*–*186*).
- Tranexamic acid is an antifibrinolytic agent (*187*).
- Doxycycline has been investigated because of its ability to inhibit matrix metalloproteinases (*188*,*189*).
- Certain treatments, such as estrogen alone or estrogen-containing contraception, likely work to decrease bleeding primarily during treatment use, whereas other drugs, such as NSAIDs, SERMs, and SPRMs, might have effects that continue after treatment is completed.
- Evidence is limited on specific drugs, doses, and durations of use for effective treatments for bleeding irregularities with ENG implant use. Therefore, this report includes general recommendations for treatments to consider rather than specific regimens.

Although the ENG implant is the only implant available in the United States, evidence from studies of both ENG and LNG implants was considered for this recommendation because the mechanisms for bleeding irregularities with both implants are similar (*190*). Evidence includes nine RCTs that examined treatments for bleeding irregularities with ENG implants and 11 RCTs that investigated treatments for bleeding irregularities with LNG implants; in addition, one placebo-controlled trial with a nonrandom method of allocation (i.e., assigned systematically, in sequence of enrollment) is described because of its historical inclusion in the evidence for this recommendation (Supplementary Appendix, https://stacks.cdc.gov/view/cdc/156517). Trials primarily reported on outcomes related to improvements in bleeding irregularities; few trials reported any side effects or adverse events. Few trials reported on patient satisfaction or implant discontinuation.

**NSAIDs. *Celecoxib:*** One small study among LNG implant users found higher proportions of participants experienced cessation of bleeding within 7 days of start of treatment as well as fewer bleeding and spotting days after treatment cessation and a longer bleed-free interval in 28 days of follow-up with oral celecoxib (200 mg) daily for 5 days compared with placebo (*191*). No trials investigated celecoxib use among ENG implant users (Certainty of evidence: high).

***Mefenamic acid:*** Two trials examined mefenamic acid; one was conducted among LNG implant users who took oral mefenamic acid (500 mg) two times daily (*192*) and one among ENG implant users who took mefenamic acid (500 mg) three times daily (*193*). Both trials found higher proportions of participants experienced cessation of bleeding within 7 days of start of treatment and improved bleeding patterns after treatment cessation in 28 days of follow-up among implant users taking mefenamic acid for 5 days compared with placebo (*192*,*193*). However, a head-to-head trial demonstrated greater cessation of bleeding within 7 days of start of treatment for daily use of a 20 *µ*g EE/150 *µ*g desogestrel COC compared with a course of mefenamic acid (500 mg) 3 times daily for 5 days among ENG implant users (*194*) (Certainty of evidence for mefenamic acid: high; certainty of evidence for mefenamic acid versus COC: very low).

***Ibuprofen:*** Ibuprofen use among LNG implant users demonstrated inconsistent effects. One trial did not demonstrate any significant differences in the number of bleeding and spotting days after a course of ibuprofen (800 mg) twice daily for 5 days compared with placebo (*195*). Another trial with a nonrandom method of allocation (i.e., assigned systematically, in sequence of enrollment) reported a reduction in number of bleeding and spotting days after initiating ibuprofen (800 mg) 3 times daily for 5 days compared with placebo (*196*). No trials investigated ibuprofen use among ENG implant users (Certainty of evidence: very low to low).

**Antifibrinolytic agents. *Tranexamic acid:*** Tranexamic acid (500 mg) twice daily for 5 days among LNG implant users increased the percentage of those who stopped bleeding within 7 days of treatment initiation compared with placebo. However, there was no difference in bleeding and spotting days after treatment cessation in the 28-day follow-up period between those using tranexamic acid and those using placebo (*197*). No trials investigated tranexamic acid among ENG implant users (Certainty of evidence: high).

**Hormonal treatment. *COCs:*** COC courses ranging from 14 to 42 days decreased bleeding on treatment compared with placebo in both LNG and ENG implant users but did not improve bleeding after treatment cessation (*198*–*201*). Three trials compared a 30 *µ*g EE/150 *µ*g LNG pill with placebo [two among ENG implant users (*199*,*200*) and one among LNG implant users (*201*)], whereas a study among LNG users compared a 50 *µ*g EE/250 *µ*g LNG pill with placebo. In addition, a 20 *µ*g EE/150 *µ*g desogestrel COC improved time to bleeding episode cessation compared with mefenamic acid among ENG implant users (*194*) (Certainty of evidence for COCs: very low to high; certainty of evidence for COC versus mefenamic acid: low).

***Estrogen:*** EE use among LNG implant users decreased bleeding on treatment compared with placebo but had inconsistent effects on bleeding patterns after treatment completion. In two RCTs and one trial with a nonrandom method of allocation (i.e., assigned systematically, in sequence of enrollment), EE (50 *µ*g) daily for approximately 3 weeks decreased bleeding and spotting while on treatment, but off-treatment effects were inconsistent (*198*,*201*); only the nonrandomized trial reported decreased bleeding and spotting days after treatment cessation for EE (50 *µ*g) users compared with placebo (*196*). EE (20 *µ*g) for 10 days (*195*) and use of an estradiol patch (100 *µ*g/day releasing) for 6 weeks did not improve bleeding irregularities compared with placebo (*202*). No trials investigated use of EE among ENG implant users (Certainty of evidence for oral EE [50 *µ*g]: very low to moderate; certainty of evidence for oral EE [20 *µ*g] and estradiol patch: very low).

**SERMs. *Tamoxifen:*** One trial of tamoxifen (10 mg) twice daily for 10 days observed decreased bleeding during and after treatment compared with placebo for LNG implant users (*203*). Two trials using tamoxifen (10 mg) twice daily for 7 days among ENG implant users observed decreased bleeding and spotting days and increased bleed-free interval after treatment cessation compared with placebo (*204*,*205*) (Certainty of evidence: high).

**Additional interventions for which evidence suggested no positive effect or evidence was too limited to make a recommendation:** Evidence on multiple other interventions was identified, including aspirin (one trial) (*206*), LNG pills (one trial) (*196*), mifepristone (three trials) (*207*–*209*), UPA (one trial) (*210*), doxycycline alone (two trials) (*208*,*209*), doxycycline combined with EE (one trial) (*209*), doxycycline combined with mifepristone (one trial) (*209*), and vitamin E (two trials) (*206*,*211*). For these interventions, the evidence either suggested no positive effect on the outcomes assessed or the evidence was too limited to make a recommendation. A detailed summary of the evidence is provided (Supplementary Appendix, https://stacks.cdc.gov/view/cdc/156517).

## Injectables

Progestin-only injectable contraceptives (DMPA, 150 mg intramuscularly [DMPA-IM] or 104 mg subcutaneously [DMPA-SC]) are available in the United States; the only difference between these two formulations is the route of administration. Approximately four out of 100 DMPA users will become pregnant in the first year with typical use (*28*). DMPA is reversible and can be used by patients of all ages, including adolescents. DMPA does not protect against STIs, including HIV infection, and patients using DMPA should be counseled that consistent and correct use of external (male) latex condoms reduces the risk for STIs, including HIV infection (*31*). Use of internal (female) condoms can provide protection from STIs, including HIV infection, although data are limited (*31*). Patients also should be counseled that PrEP, when taken as prescribed, is highly effective for preventing HIV infection (*32*).

### Initiation of Injectables

#### Timing

- The first DMPA injection may be administered at any time if it is reasonably certain that the patient is not pregnant (Box 3).

#### Need for Back-Up Contraception

- If DMPA is started within the first 7 days since menstrual bleeding started, no additional contraceptive protection is needed.
- If DMPA is started >7 days since menstrual bleeding started, the patient needs to abstain from sexual intercourse or use barrier methods (e.g., condoms) for the next 7 days.

#### Special Considerations

##### Amenorrhea (Not Postpartum)

- **Timing:** The first DMPA injection may be administered at any time if it is reasonably certain that the patient is not pregnant (Box 3).
- **Need for back-up contraception:** The patient needs to abstain from sexual intercourse or use barrier methods (e.g., condoms) for the next 7 days.

##### Postpartum (Breastfeeding)

- **Timing:** The first DMPA injection may be administered at any time, including immediately postpartum (U.S. MEC 2 if <30 days postpartum; U.S. MEC 2 if 30–42 days postpartum with other risk factors for venous thromboembolism; U.S. MEC 1 if 30–42 days postpartum without other risk factors for venous thromboembolism; U.S. MEC 1 if >42 days postpartum) (*1*), if it is reasonably certain that the patient is not pregnant (Box 3).
- **Need for back-up contraception:** If the patient is <6 months postpartum, amenorrheic, and fully or nearly fully breastfeeding (exclusively breastfeeding or the vast majority [≥85%] of feeds are breastfeeds) (*44*), no additional contraceptive protection is needed. Otherwise, a patient who is ≥21 days postpartum and whose menstrual cycle has not returned needs to abstain from sexual intercourse or use barrier methods (e.g., condoms) for the next 7 days. If the patient’s menstrual cycle has returned and it has been >7 days since menstrual bleeding started, the patient needs to abstain from sexual intercourse or use barrier methods (e.g., condoms) for the next 7 days.

##### Postpartum (Nonbreastfeeding)

- **Timing:** The first DMPA injection may be administered at any time, including immediately postpartum (U.S. MEC 2 if <21 days postpartum; U.S. MEC 2 if 21–42 days postpartum with other risk factors for venous thromboembolism; U.S. MEC 1 if 21–42 days postpartum without other risk factors for venous thromboembolism; U.S. MEC 1 if >42 days postpartum) (*1*), if it is reasonably certain that the patient is not pregnant (Box 3).
- **Need for back-up contraception:** If the patient is <21 days postpartum, no additional contraceptive protection is needed. A patient who is ≥21 days postpartum and whose menstrual cycle has not returned needs to abstain from sexual intercourse or use barrier methods (e.g., condoms) for the next 7 days. If the patient’s menstrual cycle has returned and it has been >7 days since menstrual bleeding started, the patient needs to abstain from sexual intercourse or use barrier methods (e.g., condoms) for the next 7 days.

##### Postabortion (Spontaneous or Induced)

- **Timing:** The first DMPA injection may be administered at any time postabortion, including immediately after abortion completion, if it is reasonably certain that the patient is not pregnant (Box 3), or at the time of medication abortion initiation (U.S. MEC 1 or 2) (*1*).
- After a first trimester medication abortion that included mifepristone, concurrent administration of DMPA with mifepristone might slightly decrease medication abortion effectiveness and increase risk for ongoing pregnancy (U.S. MEC 2) (*1*). Risk for ongoing pregnancy with concurrent administration of DMPA with mifepristone versus DMPA administration after abortion completion should be considered along with personal preference and access to follow-up abortion and contraceptive care.
- **Need for back-up contraception:** The patient needs to abstain from sexual intercourse or use barrier methods (e.g., condoms) for the next 7 days unless the injection is administered at the time of an abortion.

##### Switching from Another Contraceptive Method

- **Timing:** The first DMPA injection may be administered immediately if it is reasonably certain that the patient is not pregnant (Box 3). Waiting for the patient’s next menstrual cycle is unnecessary.
- **Need for back-up contraception:** If it has been >7 days since menstrual bleeding started, the patient needs to abstain from sexual intercourse or use barrier methods (e.g., condoms) for the next 7 days.
- **Switching from an IUD:** In addition to the need for back-up contraception when starting DMPA, there might be additional concerns when switching from an IUD. If the patient has had sexual intercourse since the start of their current menstrual cycle and it has been >5 days since menstrual bleeding started, theoretically, residual sperm might be in the genital tract, which could lead to fertilization if ovulation occurs. A health care provider may consider any of the following options to address the potential for residual sperm:
  º Advise the patient to retain the IUD for at least 7 days after the injection and return for IUD removal.
  º Advise the patient to abstain from sexual intercourse or use barrier methods (e.g., condoms) for 7 days before removing the IUD and switching to the new method. If it has been >5 days since menstrual bleeding started, the patient needs to abstain from sexual intercourse or use barrier methods (e.g., condoms) for the next 7 days.
  º If the patient cannot return for IUD removal and has not abstained from sexual intercourse or used barrier methods (e.g., condoms) for 7 days, advise the patient to use ECPs (with the exception of UPA) at the time of IUD removal. If it has been >5 days since menstrual bleeding started, the patient needs to abstain from sexual intercourse or use barrier methods (e.g., condoms) for the next 7 days.

**Comments and Evidence Summary.** In situations in which the health care provider is uncertain whether the patient might be pregnant, the benefits of starting DMPA likely exceed any risk; therefore, starting DMPA should be considered at any time, with a follow-up pregnancy test in 2–4 weeks. If a patient needs to use additional contraceptive protection when switching to DMPA from another contraceptive method, consider continuing their previous method for 7 days after DMPA injection. (As appropriate, see recommendations for Emergency Contraception.)

A systematic review identified eight articles examining DMPA initiation on different days of the menstrual cycle (*212*). Evidence from two studies with small sample sizes indicated that DMPA injections administered up to day 7 of the menstrual cycle inhibited ovulation; when DMPA was administered after day 7, ovulation occurred in certain women. Cervical mucus was of poor quality (i.e., not favorable for sperm penetration) in 90% of women within 24 hours of the injection (*213*–*215*) (Level of evidence: II-2, fair). Studies found that use of another contraceptive method until DMPA could be initiated (bridging option) did not help women initiate DMPA and was associated with more unintended pregnancies than immediate receipt of DMPA (*216*–*220*) (Level of evidence: I to II-3, fair to poor, indirect).

### Examinations and Tests Needed Before Initiation of an Injectable

Among healthy patients, no examinations or tests are needed before initiation of DMPA, although a baseline weight and BMI measurement might be useful for addressing any concerns about changes in weight over time (Table 3). Patients with known medical problems or other special conditions might need additional examinations or tests before being determined to be appropriate candidates for a particular method of contraception. U.S. MEC might be useful in such circumstances (*1*).

**TABLE 3:** Classification of examinations and tests needed before depot medroxyprogesterone acetate initiation

Examination or test	Class*
**Examination**	
Blood pressure	C
Weight (BMI) (weight [kg]/height [m]^2^)	—^†^
Clinical breast examination	C
Bimanual examination and cervical inspection	C
**Laboratory test**	
Glucose	C
Lipids	C
Liver enzymes	C
Hemoglobin	C
Thrombophilia	C
Cervical cytology (Papanicolaou smear)	C
STI screening with laboratory tests	C
HIV screening with laboratory tests	C

**Abbreviations:** BMI = body mass index; STI = sexually transmitted infection; U.S. MEC = *U.S. Medical Eligibility Criteria for Contraceptive Use.*

* **Class A:** Essential and mandatory in all circumstances for safe and effective use of the contraceptive method. **Class B:** Contributes substantially to safe and effective use, but implementation may be considered within the public health context, service context, or both; the risk of not performing an examination or test should be balanced against the benefits of making the contraceptive method available. **Class C:** Does not contribute substantially to safe and effective use of the contraceptive method. (**Source:** World Health Organization. Selected practice recommendations for contraceptive use, 2nd ed. Geneva, Switzerland: WHO Press; 2004.)

^†^ Weight (BMI) measurement is not needed to determine medical eligibility for any methods of contraception because all methods can be used (U.S. MEC 1) or generally can be used (U.S. MEC 2) among patients with obesity (BMI ≥30 kg/m^2^). However, measuring weight and calculating BMI at baseline might be helpful for discussing concerns about any changes in weight and whether changes might be related to use of the contraceptive method.

**Comments and Evidence Summary. Weight (BMI):** Patients with obesity (BMI ≥30 kg/m^2^) can use (U.S. MEC 1) or generally can use (U.S. MEC 2) DMPA (*1*); therefore, screening for obesity is not necessary for the safe initiation of DMPA. However, measuring weight and calculating BMI at baseline might be helpful for discussing concerns about any changes in weight and whether changes might be related to use of the contraceptive method. (See guidance on follow-up for DMPA users for evidence on weight gain with DMPA use.)

**Bimanual examination and cervical inspection:** Pelvic examination is not necessary before initiation of DMPA because it does not facilitate detection of conditions for which DMPA would be unsafe. Although patients with certain conditions or characteristics should not use (U.S. MEC 4) or generally should not use (U.S. MEC 3) DMPA (*1*), none of these conditions are likely to be detected by pelvic examination (*172*). A systematic review identified two case-control studies that compared delayed versus immediate pelvic examination before initiation of hormonal contraceptives, specifically oral contraceptives or DMPA (*23*). No differences in risk factors for cervical neoplasia, incidence of STIs, incidence of abnormal Papanicolaou smears, or incidence of abnormal wet mounts were observed (Level of evidence: II-2, fair, direct).

**Blood pressure:** Patients with hypertension generally can use DMPA (U.S. MEC 2), with the exception of patients with severe hypertension (systolic pressure of ≥160 mmHg or diastolic pressure of ≥100 mm Hg) or vascular disease who generally should not use DMPA (U.S. MEC 3) (*1*). Screening for hypertension before initiation of DMPA is not necessary because of the low prevalence of undiagnosed severe hypertension and the high likelihood that patients with these conditions already would have had them diagnosed. A systematic review did not identify any evidence regarding outcomes among women who were screened versus not screened with a blood pressure measurement before initiation of progestin-only contraceptives (*221*). The prevalence of undiagnosed hypertension among women of reproductive age is low. During 2011–2016, among women aged 20–44 years in the United States, the prevalence of hypertension was 9.3% and the prevalence of undiagnosed hypertension was approximately 1.6% (*222*).

**Glucose:** Although patients with complicated diabetes generally should not use DMPA (U.S. MEC 3) (*1*), screening for diabetes before initiation of DMPA is not necessary because of the low prevalence of undiagnosed diabetes and the high likelihood that patients with complicated diabetes would already have had the condition diagnosed. A systematic review did not identify any evidence regarding outcomes among women who were screened versus not screened with glucose measurement before initiation of hormonal contraceptives (*24*). The prevalence of diabetes among women of reproductive age is low. During 2011–2016 among women aged 20–44 years in the United States, the prevalence of diabetes was 4.5% and the prevalence of undiagnosed diabetes was 1.3% (*222*). Although hormonal contraceptives can have certain adverse effects on glucose metabolism in healthy women and women with diabetes, the overall clinical effect is minimal (*223*–*229*).

**Lipids:** Screening for dyslipidemias is not necessary for the safe initiation of injectables because of the low likelihood of clinically significant changes with use of hormonal contraceptives. A systematic review did not identify any evidence regarding outcomes among women who were screened versus not screened with lipid measurement before initiation of hormonal contraceptives (*24*). During 2015–2016, among women aged 20–39 years in the United States, 6.7% had high cholesterol, defined as total serum cholesterol >240 mg/dL (*111*). Studies have reported mixed results about the effects of hormonal methods on lipid levels among both healthy women and women with baseline lipid abnormalities, and the clinical significance of these changes is unclear (*112*–*115*).

**Liver enzymes:** Although patients with certain liver diseases generally should not use DMPA (U.S. MEC 3) (*1*), screening for liver disease before initiation of DMPA is not necessary because of the low prevalence of these conditions and the high likelihood that patients with liver disease already would have had the condition diagnosed. A systematic review did not identify any evidence regarding outcomes among women who were screened versus not screened with liver enzyme tests before initiation of hormonal contraceptives (*24*). During 2012, among U.S. women, the percentage with liver disease (not further specified) was 1.3% (*116*). During 2013, the incidence of acute hepatitis A, B, or C was ≤1 per 100,000 U.S. population (*117*). During 2002–2011, the incidence of liver cancer among U.S. women was approximately 3.7 per 100,000 population (*118*).

**Thrombophilia:** Patients with thrombophilia generally should not use DMPA (U.S. MEC 3) (*1*). However, studies have demonstrated that routine thrombophilia screening in the general population before contraceptive initiation is not cost-effective because of the rarity of the condition and high cost of screening (*230*–*234*).

**Clinical breast examination:** Although patients with current breast cancer should not use DMPA (U.S. MEC 4) (*1*), screening asymptomatic patients with a clinical breast examination before initiating DMPA is not necessary because of the low prevalence of breast cancer among women of reproductive age. A systematic review did not identify any evidence regarding outcomes among women who were screened versus not screened with a clinical breast examination before initiation of hormonal contraceptives (*23*). The incidence of breast cancer among women of reproductive age in the United States is low. During 2020, the incidence of breast cancer among women aged <50 years was approximately 45.9 per 100,000 women (*119*).

**Other screening:** Patients with iron-deficiency anemia, cervical intraepithelial neoplasia, cervical cancer, HIV infection, or other STIs can use (U.S. MEC 1) or generally can use (U.S. MEC 2) DMPA (*1*); therefore, screening for these conditions is not necessary for the safe initiation of DMPA.

### Self-Administration of Subcutaneous Injectable Contraception

- Self-administered DMPA-SC should be made available as an additional approach to deliver injectable contraception.

**Comments and Evidence Summary.** Self-administered DMPA-SC is a user-controlled method that has the potential to improve contraceptive access and increase reproductive autonomy. Self-administered DMPA-SC should be made available as an additional approach; provider-administered DMPA should remain available. Self-administered DMPA-SC should be offered in the context of shared decision-making, with a focus on patient preferences and access to the full range of contraceptive methods. Recommendations in the U.S. MEC (*1*) and U.S. SPR for provider-administered DMPA also apply to self-administered DMPA-SC. As with provider-administered DMPA, no routine follow-up is required; however, the patient should be encouraged to contact a health care provider at any time 1) to discuss side effects or other problems, 2) if there is a desire to change the method being used (including requesting provider-administered DMPA), or 3) if there are questions or concerns about reinjection (*14*). FDA labeling states that DMPA-SC is only to be administered by a health care professional (https://www.accessdata.fda.gov/drugsatfda_docs/label/2020/021583s033s034lbl.pdf). Therefore, self-administration of DMPA-SC is considered “off-label” (*14*).

A systematic review and meta-analysis of three RCTs and three prospective cohort studies compared self-administration of DMPA-SC with provider-administered DMPA-SC or DMPA-IM (*235*,*236*). Higher rates of continuation were observed with self-administration compared with provider-administration (pooled relative risk [RR] = 1.27; 95% CI = 1.16–1.39 for three RCTs and pooled RR = 1.18; 95% CI = 1.10–1.26 for three cohort studies). Pregnancy rates were low and did not differ between self-administered and provider-administered groups (four studies). Two studies found higher rates of injection site reactions with self-administered DMPA-SC compared with provider-administered DMPA-IM, and two studies found no differences. No other side effects or adverse events were increased with self-administered DMPA-SC (Certainty of evidence: moderate for RCTs and very low for observational studies for continuation; moderate for RCTs and very low for observational studies for pregnancy rates; low for RCTs and very low for observational studies for side effects).

### Routine Follow-Up After Injectable Initiation

These recommendations address when routine follow-up is recommended for safe and effective continued use of contraception for healthy patients. The recommendations refer to general situations and might vary for different users and different situations. Specific populations who might benefit from more frequent follow-up visits include adolescents, those with certain medical conditions or characteristics, and those with multiple medical conditions.

- Advise the patient that they may contact their provider at any time to discuss side effects or other problems, if they want to change the method being used, and when it is time for reinjection. No routine follow-up visit is required.
- At other routine visits, health care providers seeing injectable users should do the following:
  º Assess the patient’s satisfaction with their contraceptive method and whether they have any concerns about method use.
  º Assess any changes in health status, including medications, that would change the appropriateness of the injectable for safe and effective continued use on the basis of U.S. MEC (e.g., category 3 and 4 conditions and characteristics) (*1*).
  º Consider assessing weight changes and discussing concerns about any changes in weight and whether changes might be related to use of the contraceptive method.

**Comments and Evidence Summary.** Although no evidence exists regarding whether a routine follow-up visit after initiating DMPA improves correct or continued use, monitoring weight or BMI change over time is important for DMPA users.

A systematic review identified a limited body of evidence that examined whether weight gain in the few months after DMPA initiation predicted future weight gain (*21*). Two studies found significant differences in weight gain or BMI at follow-up periods ranging from 12 to 36 months between early weight gainers (i.e., those who gained >5% of their baseline body weight within 6 months after initiation) and those who were not early weight gainers (*237*,*238*). The differences between groups were more pronounced at 18, 24, and 36 months than at 12 months. One study found that most adolescent DMPA users who had gained >5% of their baseline weight by 3 months gained even more weight by 12 months (*239*) (Level of evidence: II-2, fair, to II-3, fair, direct).

### Timing of Repeat Injections

#### Reinjection Interval

- Provide repeat DMPA injections every 3 months (13 weeks).

#### Special Considerations

##### Early Injection

- The repeat DMPA injection may be administered early when necessary.

##### Late Injection

- The repeat DMPA injection may be administered up to 2 weeks late (15 weeks from the last injection) without requiring additional contraceptive protection.
- If the patient is >2 weeks late (>15 weeks from the last injection) for a repeat DMPA injection, they may have the injection if it is reasonably certain that they are not pregnant (Box 3). The patient needs to abstain from sexual intercourse or use barrier methods (e.g., condoms) for the next 7 days. The patient may consider the use of emergency contraception (with the exception of UPA) if appropriate.

**Comments and Evidence Summary.** No time limits exist for early injections; injections can be administered when necessary (e.g., when a patient cannot return at the routine interval). WHO has extended the time that a patient can have a late reinjection (i.e., grace period) for DMPA use from 2 weeks to 4 weeks on the basis of data from one study demonstrating low pregnancy rates through 4 weeks; however, the CDC expert group did not consider the data to be generalizable to the United States because a large proportion of women in the study were breastfeeding. Therefore, U.S. SPR recommends a grace period of 2 weeks.

A systematic review identified 12 studies evaluating time to pregnancy or ovulation after the last injection of DMPA (*240*). Although pregnancy rates were low during the 2-week interval after the reinjection date and for 4 weeks after the reinjection date, data were sparse, and one study included a large proportion of breastfeeding women (*241*–*243*). Studies also indicated a wide variation in time to ovulation after the last DMPA injection, with the majority ranging from 15 to 49 weeks from the last injection (*244*–*252*) (Level of evidence: II-2, fair, direct).

### Bleeding Irregularities (Including Amenorrhea) During Injectable Use

- Before DMPA initiation, provide counseling about potential changes in bleeding patterns during DMPA use. Amenorrhea and spotting or light bleeding are common with DMPA use, and heavy or prolonged bleeding can occur with DMPA use. These bleeding irregularities are generally not harmful but might be bothersome to the patient. Spotting, light bleeding, and heavy or prolonged bleeding might decrease with continued DMPA use.

#### Spotting or Light Bleeding

- If clinically indicated, consider an underlying health condition, such as interactions with other medications, STIs, pregnancy, thyroid disorders, or new pathologic uterine conditions (e.g., polyps or fibroids). If an underlying health condition is found, treat the condition or refer for care.
- Explore patient goals, including continued DMPA use (with or without treatment for bleeding irregularities) or discontinuation of DMPA. If the patient wants to continue DMPA use, provide reassurance, discuss options for management of bleeding irregularities if desired, and advise the patient that they may contact their provider at any time to discuss bleeding irregularities or other side effects.
- If the patient wants to discontinue DMPA at any time, offer counseling on alternative contraceptive methods and initiate another method if it is desired.
- If the patient wants treatment, the following treatment option during days of bleeding may be considered, depending on the patient’s preferences, treatment goals, and medical history:
  º NSAIDs: short-term treatment, 5–7 days

#### Heavy or Prolonged Bleeding

- If clinically indicated, consider an underlying health condition, such as interactions with other medications, STIs, pregnancy, thyroid disorders, or new pathologic uterine conditions (such as fibroids or polyps). If an underlying health condition is identified, treat the condition or refer for care.
- Explore patient goals, including continued DMPA use (with or without treatment for bleeding irregularities) or discontinuation of DMPA. If the patient wants to continue DMPA use, provide reassurance, discuss options for management of bleeding irregularities if desired, and advise the patient that they may contact their provider at any time to discuss bleeding irregularities or other side effects.
- If the patient wants to discontinue DMPA at any time, offer counseling on alternative contraceptive methods and initiate another method if it is desired.
- If the patient wants treatment, the following treatment options during days of bleeding may be considered, depending on the patient’s preferences, treatment goals, and medical history:
  º NSAIDs: short-term treatment, 5–7 days
  º Hormonal treatment: low-dose COCs or estrogen for short-term treatment, 10–20 days

#### Amenorrhea

- Amenorrhea does not require any medical treatment. Provide reassurance.
  º If a patient’s regular bleeding pattern changes abruptly to amenorrhea, consider ruling out pregnancy if clinically indicated.
- If the patient wants to discontinue DMPA, offer counseling on alternative contraceptive methods, and initiate another method if it is desired.

**Comments and Evidence Summary.** During contraceptive counseling and before initiation of DMPA, information about common side effects such as irregular bleeding should be discussed. Bleeding or spotting is common with DMPA use (*253*). In addition, amenorrhea is common after ≥1 years of continuous use (*253*,*254*). These bleeding irregularities are generally not harmful but might be bothersome to the patient. Enhanced counseling among DMPA users detailing expected bleeding patterns and reassurance that these irregularities generally are not harmful has been demonstrated to reduce DMPA discontinuation in clinical trials (*147*,*148*).

Evidence is limited on specific drugs, doses, and durations of use for effective treatments for bleeding irregularities with DMPA use. Therefore, this report includes general recommendations for treatments to consider rather than specific regimens.

A systematic review, as well as two additional studies, examined the treatment of bleeding irregularities during DMPA use (*254*–*256*). Two small studies found significant cessation of bleeding within 7 days of starting treatment among women taking valdecoxib for 5 days or mefenamic acid for 5 days compared with placebo (*257*,*258*). Treatment with EE was found to stop bleeding better than placebo during the treatment period, although rates of discontinuation were high and safety outcomes were not examined (*259*). In one small study among DMPA users who had been experiencing amenorrhea for 2 months, treatment with COCs was found to alleviate amenorrhea better than placebo (*260*). No studies examined the effects of aspirin on bleeding irregularities among DMPA users.

## Combined Hormonal Contraceptives

CHCs contain both estrogen and a progestin and include COCs (various formulations), combined transdermal patches, and combined vaginal rings. Approximately seven out of 100 CHC users become pregnant in the first year with typical use (*28*). These methods are reversible and can be used by patients of all ages. Combined hormonal contraceptives are generally used for 21–24 consecutive days, followed by 4–7 hormone-free days (either no use or placebo pills). These methods are sometimes used for an extended period with infrequent or no hormone-free days. CHCs do not protect against STIs, including HIV infection, and patients using CHCs should be counseled that consistent and correct use of external (male) latex condoms reduces the risk for STIs, including HIV infection (*31*). Use of internal (female) condoms can provide protection from STIs, including HIV infection, although data are limited (*31*). Patients also should be counseled that PrEP, when taken as prescribed, is highly effective for preventing HIV infection (*32*).

### Initiation of CHCs

#### Timing

- CHCs may be initiated at any time if it is reasonably certain that the patient is not pregnant (Box 3).

#### Need for Back-Up Contraception

- If CHCs are started within the first 5 days since menstrual bleeding started, no additional contraceptive protection is needed.
- If CHCs are started >5 days since menstrual bleeding started, the patient needs to abstain from sexual intercourse or use barrier methods (e.g., condoms) for the next 7 days.

#### Special Considerations

##### Amenorrhea (Not Postpartum)

- **Timing:** CHCs may be started at any time if it is reasonably certain that the patient is not pregnant (Box 3).
- **Need for back-up contraception:** The patient needs to abstain from sexual intercourse or use barrier methods (e.g., condoms) for the next 7 days.

##### Postpartum (Breastfeeding)

- **Timing:** CHCs may be started when the patient is medically eligible to use the method (*1*) and if it is reasonably certain that they are not pregnant. (Box 3).
- Postpartum patients who are breastfeeding should not use CHCs <21 days postpartum (U.S. MEC 4) (*1*). Postpartum patients who are breastfeeding generally should not use CHCs during 21 to <30 days postpartum (U.S. MEC 3) (*1*). Postpartum breastfeeding patients with other risk factors for venous thromboembolism generally should not use CHCs 30–42 days postpartum (U.S. MEC 3) (*1*). However, postpartum breastfeeding patients without other risk factors for venous thromboembolism generally can use CHCs 30–42 days postpartum (U.S. MEC 2) (*1*), and all breastfeeding patients generally can use CHCs >42 days postpartum (U.S. MEC 2) (*1*).
- **Need for back-up contraception:** If the patient is <6 months postpartum, amenorrheic, and fully or nearly fully breastfeeding (exclusively breastfeeding or the vast majority [≥85%] of feeds are breastfeeds) (*44*), no additional contraceptive protection is needed. Otherwise, a patient who is ≥21 days postpartum and whose menstrual cycle has not returned needs to abstain from sexual intercourse or use barrier methods (e.g., condoms) for the next 7 days. If the patient’s menstrual cycle has returned and it has been >5 days since menstrual bleeding started, the patient needs to abstain from sexual intercourse or use barrier methods (e.g., condoms) for the next 7 days.

##### Postpartum (Nonbreastfeeding)

- **Timing:** CHCs may be started when the patient is medically eligible to use the method (*1*) and if it is reasonably certain that the patient is not pregnant (Box 3).
- Postpartum patients should not use CHCs <21 days postpartum (U.S. MEC 4) (*1*). Postpartum patients with other risk factors for venous thromboembolism generally should not use CHCs 21–42 days postpartum (U.S. MEC 3) (*1*). However, postpartum patients without other risk factors for venous thromboembolism generally can use CHCs 21–42 days postpartum (U.S. MEC 2) (*1*), and all postpartum patients can use CHCs >42 days postpartum (U.S. MEC 1) (*1*).
- **Need for back-up contraception:** If the patient is <21 days postpartum, no additional contraceptive protection is needed. A patient who is ≥21 days postpartum and whose menstrual cycle has not returned needs to abstain from sexual intercourse or use barrier methods (e.g., condoms) for the next 7 days. If the patient’s menstrual cycle has returned and it has been >5 days since menstrual bleeding started, the patient needs to abstain from sexual intercourse or use barrier methods (e.g., condoms) for the next 7 days.

##### Postabortion (Spontaneous or Induced)

- **Timing:** CHCs may be started at any time postabortion, including immediately after abortion completion, if it is reasonably certain that the patient is not pregnant (Box 3), or at the time of medication abortion initiation (U.S. MEC 1) (*1*).
- **Need for back-up contraception:** The patient needs to abstain from sexual intercourse or use barrier methods (e.g., condoms) for the next 7 days unless CHCs are started at the time of an abortion.

##### Switching from Another Contraceptive Method

- **Timing:** CHCs may be started immediately if it is reasonably certain that the patient is not pregnant (Box 3). Waiting for the patient’s next menstrual cycle is unnecessary.
- **Need for back-up contraception:** If it has been >5 days since menstrual bleeding started, the patient needs to abstain from sexual intercourse or use barrier methods (e.g., condoms) for the next 7 days.
- **Switching from an IUD:** In addition to the need for back-up contraception when starting CHCs, there might be additional concerns when switching from an IUD. If the patient has had sexual intercourse since the start of their current menstrual cycle and it has been >5 days since menstrual bleeding started, theoretically, residual sperm might be in the genital tract, which could lead to fertilization if ovulation occurs. A health care provider may consider any of the following options to address the potential for residual sperm:
  º Advise the patient to retain the IUD for at least 7 days after CHCs are initiated and return for IUD removal.
  º Advise the patient to abstain from sexual intercourse or use barrier methods (e.g., condoms) for 7 days before removing the IUD and switching to the new method. If it has been >5 days since menstrual bleeding started, the patient needs to abstain from sexual intercourse or use barrier methods (e.g., condoms) for the next 7 days.
  º If the patient cannot return for IUD removal and has not abstained from sexual intercourse or used barrier methods (e.g., condoms) for 7 days, advise the patient to use ECPs at the time of IUD removal. CHCs may be started immediately after use of ECPs (with the exception of UPA). CHCs may be started no sooner than 5 days after use of UPA. If it has been >5 days since menstrual bleeding started, the patient needs to abstain from sexual intercourse or use barrier methods (e.g., condoms) for the next 7 days.

**Comments and Evidence Summary.** In situations in which the health care provider is uncertain whether the patient might be pregnant, the benefits of starting CHCs likely exceed any risk; therefore, starting CHCs should be considered at any time, with a follow-up pregnancy test in 2–4 weeks. If a patient needs to use additional contraceptive protection when switching to CHCs from another contraceptive method, consider continuing their previous method for 7 days after starting CHCs. (As appropriate, see recommendations for Emergency Contraception.)

A systematic review of 18 studies examined the effects of starting CHCs on different days of the menstrual cycle (*261*). Overall, the evidence suggested that pregnancy rates did not differ by the timing of CHC initiation (*220*,*262*–*264*) (Level of evidence: I to II-3, fair, indirect). The more follicular activity that occurred before starting COCs, the more likely ovulation was to occur; however, no ovulations occurred when COCs were started at a follicle diameter of 10 mm (mean cycle day 7.6) or when the ring was started at 13 mm (median cycle day 11) (*265*–*274*) (Level of evidence: I to II-3, fair, indirect). Bleeding patterns and other side effects did not vary with the timing of CHC initiation (*263*,*264*,*275*–*279*) (Level of evidence: I to II-2, good to poor, direct). Although continuation rates of CHCs were initially improved by the “quick start” approach (i.e., starting on the day of the visit), the advantage disappeared over time (*262*,*263*,*275*–*280*) (Level of evidence: I to II-2, good to poor, direct).

### Examinations and Tests Needed Before Initiation of CHCs

Among healthy patients, few examinations or tests are needed before initiation of CHCs (Table 4). Blood pressure should be measured before initiation of combined hormonal contraceptives. Baseline weight and BMI measurements might be useful for addressing any concerns about changes in weight over time. Patients with known medical problems or other special conditions might need additional examinations or tests before being determined to be appropriate candidates for a particular method of contraception. U.S. MEC might be useful in such circumstances (*1*).

**TABLE 4:** Classification of examinations and tests needed before combined hormonal contraceptive initiation

Examination or test	Class*
**Examination**	
Blood pressure	A^†^
Weight (BMI) (weight [kg]/height [m]^2^)	—^§^
Clinical breast examination	C
Bimanual examination and cervical inspection	C
**Laboratory test**	
Glucose	C
Lipids	C
Liver enzymes	C
Hemoglobin	C
Thrombophilia	C
Cervical cytology (Papanicolaou smear)	C
STI screening with laboratory tests	C
HIV screening with laboratory tests	C

**Abbreviations:** BMI = body mass index; STI = sexually transmitted infection; U.S. MEC = *U.S. Medical Eligibility Criteria for Contraceptive Use.*

* **Class A:** Essential and mandatory in all circumstances for safe and effective use of the contraceptive method. **Class B:** Contributes substantially to safe and effective use, but implementation may be considered within the public health context, service context, or both; the risk of not performing an examination or test should be balanced against the benefits of making the contraceptive method available. **Class C:** Does not contribute substantially to safe and effective use of the contraceptive method. (**Source:** World Health Organization. Selected practice recommendations for contraceptive use, 2nd ed. Geneva, Switzerland: WHO Press; 2004.)

^†^ In instances in which blood pressure cannot be measured by a provider, blood pressure measured in other settings can be reported by the patient to their provider.

^§^ Weight (BMI) measurement is not needed to determine medical eligibility for any methods of contraception because all methods can be used (U.S. MEC 1) or generally can be used (U.S. MEC 2) among patients with obesity (BMI ≥30 kg/m^2^). However, measuring weight and calculating BMI at baseline might be helpful for discussing concerns about any changes in weight and whether changes might be related to use of the contraceptive method.

**Comments and Evidence Summary. Blood pressure:** Patients who have more severe hypertension (systolic pressure of ≥160 mm Hg or diastolic pressure of ≥100 mm Hg) or vascular disease should not use CHCs (U.S. MEC 4) (*1*), and patients who have less severe hypertension (systolic pressure of 140–159 mm Hg or diastolic pressure of 90–99 mm Hg) or adequately controlled hypertension generally should not use CHCs (U.S. MEC 3) (*1*). Therefore, blood pressure should be evaluated before initiating CHCs. In instances in which blood pressure cannot be measured by a provider, blood pressure measured in other settings can be reported by the patient to their provider. Evidence suggests that cardiovascular outcomes are worse among women who did not have their blood pressure measured before initiating COCs. A systematic review identified six articles from three studies that reported cardiovascular outcomes among women who had blood pressure measurements and women who did not have blood pressure measurements before initiating COCs (*221*). Three case-control studies demonstrated that women who did not have blood pressure measurements before initiating COCs had a higher risk for acute myocardial infarction than women who did have blood pressure measurements (*281*–*283*). Two case-control studies demonstrated that women who did not have blood pressure measurements before initiating COCs had a higher risk for ischemic stroke than women who did have blood pressure measurements (*284*,*285*). One case-control study reported no difference in the risk for hemorrhagic stroke among women who initiated COCs regardless of whether their blood pressure was measured (*286*). Studies that examined hormonal contraceptive methods other than COCs were not identified (Level of evidence: II-2, fair, direct).

**Weight (BMI):** Patients with obesity (BMI ≥30 kg/m^2^) generally can use CHCs (U.S. MEC 2) (*1*); therefore, screening for obesity is not necessary for the safe initiation of CHCs. However, measuring weight and calculating BMI at baseline might be helpful for discussing concerns about any changes in weight and whether changes might be related to use of the contraceptive method.

**Bimanual examination and cervical inspection:** Pelvic examination is not necessary before initiation of CHCs because it does not facilitate detection of conditions for which hormonal contraceptives would be unsafe. Although patients with certain conditions or characteristics should not use (U.S. MEC 4) or generally should not use (U.S. MEC 3) CHCs (*1*), none of these conditions are likely to be detected by pelvic examination (*172*). A systematic review identified two case-control studies that compared delayed and immediate pelvic examination before initiation of hormonal contraceptives, specifically oral contraceptives or DMPA (*23*). No differences in risk factors for cervical neoplasia, incidence of STIs, incidence of abnormal Papanicolaou smears, or incidence of abnormal wet mounts were found (Level of evidence: II-2 fair, direct).

**Glucose:** Although patients with complicated diabetes should not use (U.S. MEC 4) or generally should not use (U.S. MEC 3) CHCs, depending on the severity of the condition (*1*), screening for diabetes before initiation of hormonal contraceptives is not necessary because of the low prevalence of undiagnosed diabetes and the high likelihood that patients with complicated diabetes already would have had the condition diagnosed. A systematic review did not identify any evidence regarding outcomes among women who were screened versus not screened with glucose measurement before initiation of hormonal contraceptives (*24*). The prevalence of diabetes among women of reproductive age is low. During 2011–2016 among women aged 20–44 years in the United States, the prevalence of diabetes was 4.5% and the prevalence of undiagnosed diabetes was 1.3% (*222*). Although hormonal contraceptives can have certain adverse effects on glucose metabolism in healthy women and women with diabetes, the overall clinical effect is minimal (*223*–*229*).

**Lipids:** Screening for dyslipidemias is not necessary for the safe initiation of CHCs because of the low likelihood of clinically significant changes with use of hormonal contraceptives. A systematic review did not identify any evidence regarding outcomes among women who were screened versus not screened with lipid measurement before initiation of hormonal contraceptives (*24*). During 2015–2016 among women aged 20–39 years in the United States, 6.7% had high cholesterol, defined as total serum cholesterol >240 mg/dL (*111*). A systematic review identified few studies, all of poor quality, that suggest that women with known dyslipidemias using CHCs might be at increased risk for myocardial infarction, cerebrovascular accident, or venous thromboembolism compared with women without dyslipidemias; no studies were identified that examined risk for pancreatitis among women with known dyslipidemias using CHCs (*115*). Studies have reported mixed results regarding the effects of hormonal contraceptives on lipid levels among both healthy women and women with baseline lipid abnormalities, and the clinical significance of these changes is unclear (*112*–*115*).

**Liver enzymes:** Although patients with certain liver diseases should not use (U.S. MEC 4) or generally should not use (U.S. MEC 3) CHCs (*1*), screening for liver disease before initiation of CHCs is not necessary because of the low prevalence of these conditions and the high likelihood that patients with liver disease already would have had the condition diagnosed. A systematic review did not identify any evidence regarding outcomes among women who were screened versus not screened with liver enzyme tests before initiation of hormonal contraceptives (*24*). During 2012, among U.S. women, the percentage with liver disease (not further specified) was 1.3% (*116*). During 2013, the incidence of acute hepatitis A, B, or C was ≤1 per 100,000 U.S. population (*117*). During 2002–2011, the incidence of liver cancer among U.S. women was approximately 3.7 per 100,000 population (*118*).

**Thrombophilia:** Patients with thrombophilia should not use CHCs (U.S. MEC 4) (*1*). However, studies have demonstrated that routine thrombophilia screening in the general population before contraceptive initiation is not cost-effective because of the rarity of the conditions and high cost of screening (*230*–*234*).

**Clinical breast examination:** Although patients with current breast cancer should not use CHCs (U.S. MEC 4) (*1*), screening asymptomatic patients with a clinical breast examination before initiating CHCs is not necessary because of the low prevalence of breast cancer among women of reproductive age. A systematic review did not identify any evidence regarding outcomes among women who were screened versus not screened with a breast examination before initiation of hormonal contraceptives (*23*). The incidence of breast cancer among women of reproductive age in the United States is low. During 2020, the incidence of breast cancer among women aged <50 years was approximately 45.9 per 100,000 women (*119*).

**Other screening:** Patients with iron-deficiency anemia, cervical intraepithelial neoplasia, cervical cancer, HIV infection, or other STIs can use (U.S. MEC 1) or generally can use (U.S. MEC 2) CHCs (*1*). Therefore, screening for these conditions is not necessary for the safe initiation of combined hormonal contraceptives.

### Number of Pill Packs that Should Be Provided at Initial and Return Visits

- At the initial and return visits, provide or prescribe up to a 1-year supply of COCs (e.g., 13 28-day pill packs), depending on the patient’s preferences and anticipated use.
- A patient should be able to obtain COCs easily in the amount and at the time they need them.

**Comments and Evidence Summary.** The more pill packs provided up to 13 cycles, the higher the continuation rates. Restricting the number of pill packs distributed or prescribed can be a barrier for patients who want to continue COC use and might increase risk for pregnancy.

A systematic review of the evidence suggested that providing a greater number of pill packs was associated with increased continuation (*20*). Studies that compared provision of one versus 12 packs, one versus 12 or 13 packs, or three versus seven packs found increased continuation of pill use among women provided with more pill packs (*287*–*289*). However, one study found no difference in continuation when patients were provided one and then three packs versus four packs all at once (*290*). In addition to continuation, a greater number of pill packs provided was associated with fewer pregnancy tests, fewer pregnancies, and lower cost per client. However, a greater number of pill packs (i.e., 13 packs versus three packs) also was associated with increased pill wastage in one study (*288*) (Level of evidence: I to II-2, fair, direct).

### Routine Follow-Up After CHC Initiation

These recommendations address when routine follow-up is recommended for safe and effective continued use of contraception for healthy patients. The recommendations refer to general situations and might vary for different users and different situations. Specific populations who might benefit from more frequent follow-up visits include adolescents, those with certain medical conditions or characteristics, and those with multiple medical conditions.

- Advise the patient that they may contact their provider at any time to discuss side effects or other problems or if they want to change the method being used. No routine follow-up visit is required.
- At other routine visits, health care providers seeing CHC users should do the following:
  º Assess the patient’s satisfaction with their contraceptive method and whether they have any concerns about method use.
  º Assess any changes in health status, including medications, that would change the appropriateness of CHCs for safe and effective continued use on the basis of U.S. MEC (e.g., category 3 and 4 conditions and characteristics) (*1*).
  º Assess blood pressure.
  º Consider assessing weight changes and discussing concerns about any changes in weight and whether changes might be related to use of the contraceptive method.

**Comments and Evidence Summary.** No evidence exists regarding whether a routine follow-up visit after initiating CHCs improves correct or continued use. Monitoring blood pressure is important for CHC users. Health care providers might consider recommending patients obtain blood pressure measurements in other settings, including self-measured blood pressure.

A systematic review identified five studies that examined the incidence of hypertension among women who began using a COC versus those who started a nonhormonal method of contraception or a placebo (*21*). Few women developed hypertension after initiating COCs, and studies examining increases in blood pressure after COC initiation found mixed results. No studies were identified that examined changes in blood pressure among patch or vaginal ring users (Level of evidence: I, fair, to II-2, fair, indirect).

### Late or Missed Doses and Side Effects from CHC Use

**FIGURE 1:**
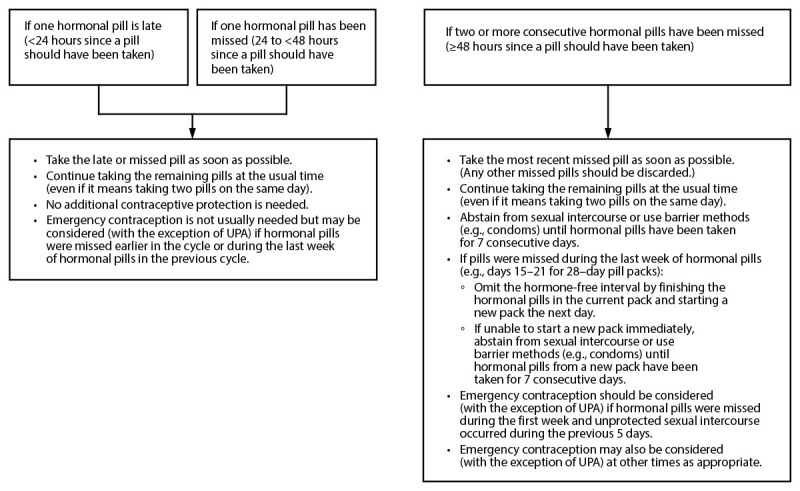
Recommended actions after late or missed combined oral contraceptives **Abbreviation:** UPA = ulipristal acetate.

**FIGURE 2:**
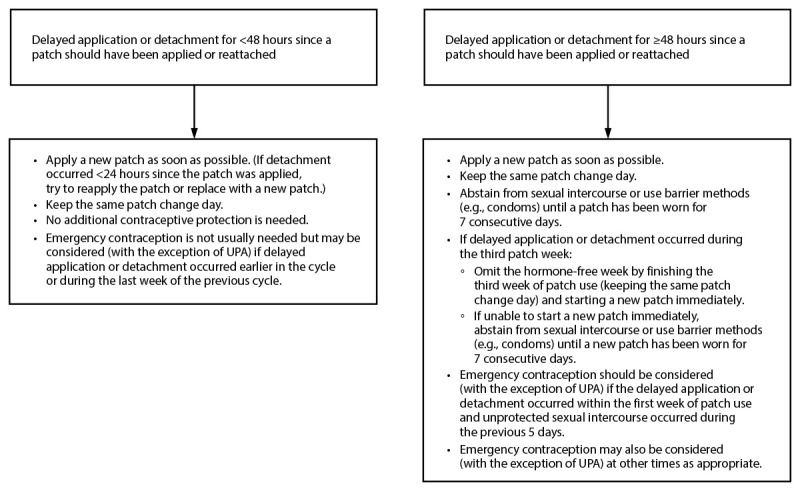
Recommended actions after delayed application or detachment* with combined hormonal patch **Abbreviation:** UPA = ulipristal acetate. * If detachment takes place but the patient is unsure when the detachment occurred, consider the patch to have been detached for ≥48 hours since a patch should have been applied or reattached.

**FIGURE 3:**
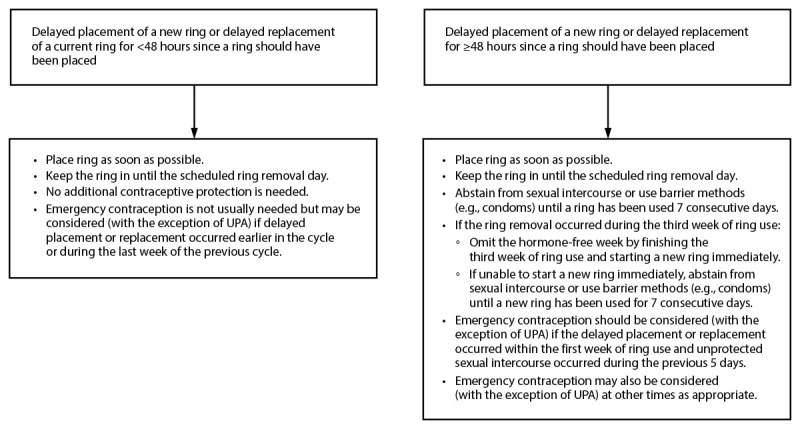
Recommended actions after delayed placement or replacement* with combined vaginal ring (etonogestrel/ethinyl estradiol)^†^ **Abbreviation:** UPA = ulipristal acetate. * If removal takes place but the patient is unsure when the ring was removed, consider the ring to have been removed for ≥48 hours since a ring should have been placed or replaced. ^†^ These recommendations are based on evidence for the etonogestrel/ethinyl estradiol combined vaginal ring. For dosing errors with the segesterone acetate/ethinyl estradiol vaginal ring, please see the package label.

For the following recommendations, a dose is considered late when <24 hours have elapsed since the dose should have been taken. A dose is considered missed if ≥24 hours have elapsed since the dose should have been taken. For example, if a COC pill was supposed to have been taken on Monday at 9:00 a.m. and is taken at 11:00 a.m., the pill is late; however, by Tuesday morning at 11:00 a.m., Monday’s 9:00 a.m. pill has been missed and Tuesday’s 9:00 a.m. pill is late. For COCs, the recommendations only apply to late or missed hormonally active pills and not to placebo pills. Recommendations are provided for late or missed pills (Figure 1), the patch (Figure 2), and the ring (Figure 3).

**Comments and Evidence Summary.** Inconsistent or incorrect use of CHCs is a major cause of CHC failure. Extending the hormone-free interval (e.g., missing hormonally active pills either directly before or after the placebo or pill-free interval) is considered to be a particularly risky time to miss CHCs. Seven days of continuous CHC use is deemed necessary to reliably prevent ovulation. The recommendations reflect a balance between the complexity of the evidence and determination of a simple and feasible recommendation. For patients who frequently miss COCs or experience other usage errors with combined transdermal patches or combined vaginal rings, explore patient goals, consider offering counseling on alternative contraceptive methods, and initiate another method if it is desired.

A systematic review identified 36 studies that examined measures of contraceptive effectiveness of CHCs during cycles with extended hormone-free intervals, shortened hormone-free intervals, or deliberate nonadherence on days not adjacent to the hormone-free interval (*291*). Most of the studies examined COCs (*274**,**292**–**319*), two examined the combined transdermal patch (*313*,*320*), and six examined the combined vaginal ring (etonogestrel/EE) (*270*,*321*–*325*). No direct evidence on the effect of missed pills on the risk for pregnancy was found. Studies of women deliberately extending the hormone-free interval up to 14 days found wide variability in the amount of follicular development and occurrence of ovulation (*295*,*298*,*300*,*301*,*303*,*304*,*306*–*309*); in general, the risk for ovulation was low, and among women who did ovulate, cycles were usually abnormal. In studies of women who deliberately missed pills on various days during the cycle not adjacent to the hormone-free interval, ovulation occurred infrequently (*293*,*299*–*301*,*309*,*310*,*312*,*313*). Studies comparing 7-day hormone-free intervals with shorter hormone-free intervals found lower rates of pregnancy (*292*,*296*,*305*,*311*) and significantly greater suppression of ovulation (*294*,*304*,*315*–*317*,*319*) among women with shorter intervals in all but one study (*314*), which found no difference. Two studies that compared 30-*µ*g EE pills with 20-*µ*g EE pills demonstrated more follicular activity when 20-*µ*g EE pills were missed (*295*,*298*). In studies examining the combined vaginal ring, three studies found that nondeliberate extension of the hormone-free interval for 24 to <48 hours from the scheduled period did not increase the risk for pregnancy (*321*,*322*,*324*); one study found that ring placement after a deliberately extended hormone-free interval that allowed a 13-mm follicle to develop interrupted ovarian function and further follicular growth (*270*); and one study found that inhibition of ovulation was maintained after deliberately forgetting to remove the ring for up to 2 weeks after normal ring use (*325*). In studies examining the combined transdermal patch, one study found that missing 1–3 consecutive days before patch replacement (either wearing one patch 3 days longer before replacement or going 3 days without a patch before replacing the next patch) on days not adjacent to the patch-free interval resulted in little follicular activity and low risk for ovulation (*313*), and one pharmacokinetic study found that serum levels of EE and progestin norelgestromin remained within reference ranges after extending patch wear for 3 days (*320*). No studies were found on extending the patch-free interval. In studies that provide indirect evidence on the effects of missed combined hormonal contraception on surrogate measures of pregnancy, how differences in surrogate measures correspond to pregnancy risk is unclear (Level of evidence: I, good, indirect to II-3, poor, direct).

### Vomiting or Severe Diarrhea While Using COCs

**FIGURE 4:**
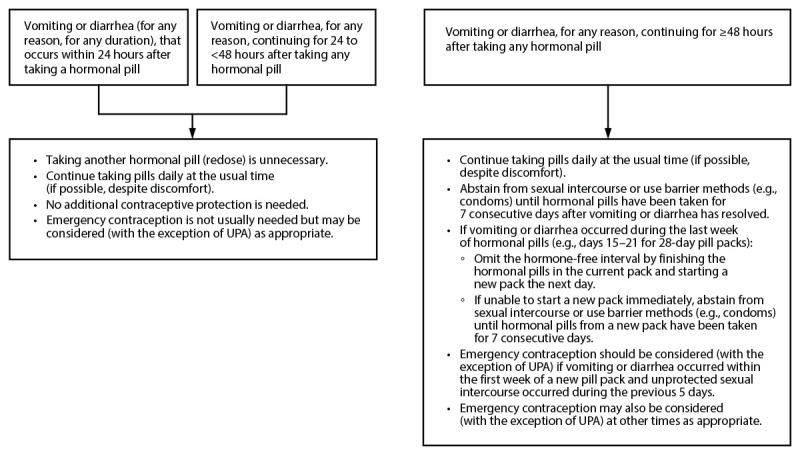
Recommended actions after vomiting or diarrhea while using combined oral contraceptives **Abbreviation:** UPA = ulipristal acetate.

Certain steps should be taken by patients who experience vomiting or severe diarrhea while using COCs (Figure 4).

**Comments and Evidence Summary.** Theoretically, the contraceptive effectiveness of COCs might be decreased because of vomiting or severe diarrhea. Because of the lack of evidence that addresses vomiting or severe diarrhea while using COCs, these recommendations are based on the recommendations for missed COCs. No evidence was found on the effects of vomiting or diarrhea on measures of contraceptive effectiveness including pregnancy, follicular development, hormone levels, or cervical mucus quality.

### Bleeding Irregularities with Extended or Continuous Use of CHCs

- Before initiation of CHCs, provide counseling about potential changes in bleeding patterns during extended or continuous CHC use. Extended contraceptive use has been defined as a planned hormone-free interval after more than 28 days of active hormones. Continuous contraceptive use has been defined as uninterrupted use of hormonal contraception without a hormone-free interval (*326*).
- Spotting or bleeding is common during the first 3–6 months of extended or continuous CHC use. Spotting or bleeding is generally not harmful but might be bothersome to the patient. Bleeding changes generally decrease with continued CHC use.
- If clinically indicated, consider an underlying health condition, such as inconsistent use, interactions with other medications, cigarette smoking, STIs, pregnancy, thyroid disorders, or new pathologic uterine conditions (e.g., polyps or fibroids). If an underlying health condition is found, treat the condition or refer for care.
- Explore patient goals, including continued CHCs (with or without treatment for bleeding irregularities) or discontinuation of CHCs. If the patient wants to continue CHCs, provide reassurance, discuss options for management of bleeding irregularities if it is desired, and advise the patient that they may contact their provider at any time to discuss bleeding irregularities or other side effects.
- If the patient wants to discontinue CHCs at any time, offer counseling on alternative contraceptive methods, and initiate another method if it is desired.
- If the patient wants treatment, the following treatment option may be considered:
  º Advise the patient to discontinue CHC use (i.e., a hormone-free interval) for 3–4 consecutive days; a hormone-free interval is not recommended during the first 21 days of using the continuous or extended CHC method. A hormone-free interval also is not recommended more than once per month because contraceptive effectiveness might be reduced.

**Comments and Evidence Summary.** During contraceptive counseling and before initiating extended or continuous CHCs, information about common side effects such as spotting or bleeding, especially during the first 3–6 months of use, should be discussed (*327*). These bleeding irregularities are generally not harmful but might be bothersome to the patient. Bleeding irregularities usually improve with persistent use of the hormonal method. To avoid spotting or bleeding, counseling should emphasize the importance of correct use and timing; for users of contraceptive pills, emphasize consistent pill use. Enhanced counseling about expected bleeding patterns and reassurance that bleeding irregularities are generally not harmful has been demonstrated to reduce method discontinuation in clinical trials with other hormonal contraceptives (i.e., DMPA) (*147*,*148*,*328*).

A systematic review identified three studies with small study populations that addressed treatments for breakthrough bleeding among women using extended or continuous CHCs (*329*). In two separate RCTs in which women were taking either contraceptive pills or using the contraceptive ring continuously for 168 days, women assigned to a hormone-free interval of 3 or 4 days reported improved bleeding. Although they noted an initial increase in flow, this was followed by an abrupt decrease 7–8 days later with eventual cessation of flow 11–12 days later. These findings were compared with those among women who continued to use their method without a hormone-free interval, in which a greater proportion reported either treatment failure or fewer days of amenorrhea (*330*,*331*). In another randomized trial of 66 women with breakthrough bleeding among women using 84 days of hormonally active contraceptive pills, oral doxycycline (100 mg twice daily) initiated the first day of bleeding and taken for 5 days did not result in any improvement in bleeding compared with placebo (*332*) (Level of evidence: I, fair, direct).

## Progestin-Only Pills

POPs contain only a progestin and no estrogen. Three formulations are currently available in the United States: norethindrone, norgestrel, and drospirenone (DRSP). Approximately seven out of 100 POP users become pregnant in the first year with typical use (*28*). POPs are reversible and can be used by patients of all ages. POPs do not protect against STIs, including HIV infection, and patients using POPs should be counseled that consistent and correct use of external (male) latex condoms reduces the risk for STIs, including HIV infection (*31*). Use of internal (female) condoms can provide protection from STIs, including HIV infection, although data are limited (*31*). Patients also should be counseled that PrEP, when taken as prescribed, is highly effective for preventing HIV infection (*32*).

### Initiation of POPs

#### Timing

- All POPs may be started at any time if it is reasonably certain that the patient is not pregnant (Box 3).

#### Need for Back-Up Contraception

- Norethindrone or norgestrel POPs:
  º If norethindrone or norgestrel POPs are started within the first 5 days since menstrual bleeding started, no additional contraceptive protection is needed.
  º If norethindrone or norgestrel POPs are started >5 days since menstrual bleeding started, the patient needs to abstain from sexual intercourse or use barrier methods (e.g., condoms) for the next 2 days.
- DRSP POPs:
  º If DRSP POPs are started on the first day of menstrual bleeding, no additional contraceptive protection is needed.
  º If DRSP POPs are started >1 day since menstrual bleeding started, the patient needs to abstain from sexual intercourse or use barrier methods (e.g., condoms) for the next 7 days.

#### Special Considerations

##### Amenorrhea (Not Postpartum)

- **Timing:** All POPs may be started at any time if it is reasonably certain that the patient is not pregnant (Box 3).
- **Need for back-up contraception:**
  º Norethindrone or norgestrel POPs: The patient needs to abstain from sexual intercourse or use barrier methods (e.g., condoms) for the next 2 days.
  º DRSP POPs: The patient needs to abstain from sexual intercourse or use barrier methods (e.g., condoms) for the next 7 days.

##### Postpartum (Breastfeeding)

- **Timing:** All POPs may be started at any time, including immediately postpartum (U.S. MEC 2 if <30 days postpartum; U.S. MEC 1 if ≥30 days postpartum) (*1*), if it is reasonably certain that the patient is not pregnant (Box 3).
- **Need for back-up contraception:** If the patient is <6 months postpartum, amenorrheic, and fully or nearly fully breastfeeding (exclusively breastfeeding or the vast majority [≥85%] of feeds are breastfeeds) (*44*), no additional contraceptive protection is needed.
  º Norethindrone or norgestrel POPs: A patient who is ≥21 days postpartum and whose menstrual cycle has not returned needs to abstain from sexual intercourse or use barrier methods (e.g., condoms) for the next 2 days. If the patient’s menstrual cycle has returned and it has been >5 days since menstrual bleeding started, the patient needs to abstain from sexual intercourse or use barrier methods (e.g., condoms) for the next 2 days.
  º DRSP POPs: A patient who is ≥21 days postpartum and whose menstrual cycle has not returned needs to abstain from sexual intercourse or use barrier methods (e.g., condoms) for the next 7 days. If the patient’s menstrual cycle has returned and it has been >1 day since menstrual bleeding started, the patient needs to abstain from sexual intercourse or use barrier methods (e.g., condoms) for the next 7 days.

##### Postpartum (Nonbreastfeeding)

- **Timing:** All POPs may be started at any time, including immediately postpartum (U.S. MEC 1) (*1*), if it is reasonably certain that the patient is not pregnant (Box 3).
- **Need for back-up contraception:** If the patient is <21 days postpartum, no additional contraceptive protection is needed.
  º Norethindrone or norgestrel POPs: A patient who is ≥21 days postpartum and whose menstrual cycle has not returned needs to abstain from sexual intercourse or use barrier methods (e.g., condoms) for the next 2 days. If the patient’s menstrual cycle has returned and it has been >5 days since menstrual bleeding started, the patient needs to abstain from sexual intercourse or use barrier methods (e.g., condoms) for the next 2 days.
  º DRSP POPs: A patient who is ≥21 days postpartum and whose menstrual cycle has not returned needs to abstain from sexual intercourse or use barrier methods (e.g., condoms) for the next 7 days. If the patient’s menstrual cycle has returned and it has been >1 day since menstrual bleeding started, the patient needs to abstain from sexual intercourse or use barrier methods (e.g., condoms) for the next 7 days.

##### Postabortion (Spontaneous or Induced)

- **Timing:** All POPs may be started at any time postabortion, including immediately after abortion completion, if it is reasonably certain that the patient is not pregnant (Box 3), or at the time of medication abortion initiation (U.S. MEC 1) (*1*).
- **Need for back-up contraception:** The patient needs to abstain from sexual intercourse or use barrier methods (e.g., condoms) for the next 2 days for norethindrone or norgestrel POPs or for the next 7 days for DRSP POPs, unless POPs are started at the time of an abortion.

##### Switching from Another Contraceptive Method

- **Timing:** All POPs may be started immediately if it is reasonably certain that the patient is not pregnant (Box 3). Waiting for the patient’s next menstrual cycle is unnecessary.
- **Need for back-up contraception:**
  º Norethindrone or norgestrel POPs: If it has been >5 days since menstrual bleeding started, the patient needs to abstain from sexual intercourse or use barrier methods (e.g., condoms) for the next 2 days.
  º DRSP POPs: If it has been >1 day since menstrual bleeding started, the patient needs to abstain from sexual intercourse or use barrier methods (e.g., condoms) for the next 7 days.
- **Switching from an IUD:** In addition to the need for back-up contraception when starting POPs, there might be additional concerns when switching from an IUD. If the patient has had sexual intercourse since the start of their current menstrual cycle and it has been >5 days since menstrual bleeding started, theoretically, residual sperm might be in the genital tract, which could lead to fertilization if ovulation occurs. A health care provider may consider any of the following options to address the potential for residual sperm:
  º Advise the patient to retain the IUD for at least 7 days after POPs are initiated and return for IUD removal.
  º Advise the patient to abstain from sexual intercourse or use barrier methods (e.g., condoms) for 7 days before removing the IUD and switching to the new method. The patient should also follow the back-up contraception recommendations for either norethindrone or norgestrel POPs or for DRSP POPs.
  º If the patient cannot return for IUD removal and has not abstained from sexual intercourse or used barrier methods (e.g., condoms) for 7 days, advise the patient to use ECPs at the time of IUD removal. All POPs may be started immediately after use of ECPs (with the exception of UPA). All POPs may be started no sooner than 5 days after use of UPA. The patient should also follow the back-up contraception recommendations for either norethindrone or norgestrel POPs or for DRSP POPs.

**Comments and Evidence Summary.** In situations in which the health care provider is uncertain whether the patient might be pregnant, the benefits of starting POPs likely exceed any risk. Therefore, starting POPs should be considered at any time, with a follow-up pregnancy test in 2–4 weeks. (As appropriate, see recommendations for Emergency Contraception.)

**Norethindrone or norgestrel POPs:** Unlike COCs, which inhibit ovulation as the primary mechanism of action, norethindrone or norgestrel POPs inhibit ovulation in about half of cycles, although the rates vary widely by person (*333*). Peak serum steroid levels are reached about 2 hours after administration, followed by rapid distribution and elimination, such that by 24 hours after administration, serum steroid levels are near baseline (*333*). Therefore, taking norethindrone or norgestrel POPs at approximately the same time each day is important. An estimated 48 hours of norethindrone or norgestrel POP use has been deemed necessary to achieve the contraceptive effects on cervical mucus (*333*). If a patient needs to use additional contraceptive protection when switching to norethindrone or norgestrel POPs from another contraceptive method, consider continuing their previous method for 2 days after starting norethindrone or norgestrel POPs. No direct evidence was found regarding the effects of starting norethindrone or norgestrel POPs at different times of the cycle.

**DRSP POPs:** DRSP POPs are more similar in mechanism of action to COCs, with inhibition of ovulation as the primary mechanism of action (*334*). Therefore, the recommendations for starting and using a back-up method are similar to COC recommendations. If a patient needs to use additional contraceptive protection when switching to DRSP POPs from another contraceptive method, consider continuing their previous method for 7 days after starting DRSP POPs. No direct evidence was found regarding the effects of starting DRSP POPs at different times of the cycle.

### Examinations and Tests Needed Before Initiation of POPs

Among healthy patients, no examinations or tests are needed before initiation of POPs, although a baseline weight and BMI measurement might be useful for addressing any concerns about changes in weight over time (Table 5). Patients with known medical problems or other special conditions might need additional examinations or tests before being determined to be appropriate candidates for a particular method of contraception. The U.S. MEC might be useful in such circumstances (*1*).

**TABLE 5:** Classification of examinations and tests needed before progestin-only pill initiation

Examination or test	Class*
**Examination**	
Blood pressure	C
Weight (BMI) (weight [kg]/height [m]^2^)	—^†^
Clinical breast examination	C
Bimanual examination and cervical inspection	C
**Laboratory test**	
Glucose	C
Lipids	C
Liver enzymes	C
Hemoglobin	C
Thrombophilia	C
Cervical cytology (Papanicolaou smear)	C
STI screening with laboratory tests	C
HIV screening with laboratory tests	C

**Abbreviations:** BMI = body mass index; STI = sexually transmitted infection; U.S. MEC = *U.S. Medical Eligibility Criteria for Contraceptive Use.*

* **Class A:** Essential and mandatory in all circumstances for safe and effective use of the contraceptive method. **Class B:** Contributes substantially to safe and effective use, but implementation may be considered within the public health context, service context, or both; the risk of not performing an examination or test should be balanced against the benefits of making the contraceptive method available. **Class C:** Does not contribute substantially to safe and effective use of the contraceptive method. (**Source:** World Health Organization. Selected practice recommendations for contraceptive use, 2nd ed. Geneva, Switzerland: WHO Press; 2004.)

^†^ Weight (BMI) measurement is not needed to determine medical eligibility for any methods of contraception because all methods can be used (U.S. MEC 1) or generally can be used (U.S. MEC 2) among patients with obesity (BMI ≥30 kg/m^2^). However, measuring weight and calculating BMI at baseline might be helpful for discussing concerns about any changes in weight and whether changes might be related to use of the contraceptive method.

**Comments and Evidence Summary. Weight (BMI):** Patients with obesity (BMI ≥30 kg/m^2^) can use POPs (U.S. MEC 1) (*1*); therefore, screening for obesity is not necessary for the safe initiation of POPs. However, measuring weight and calculating BMI at baseline might be helpful for discussing concerns about any changes in weight and whether changes might be related to use of the contraceptive method.

**Bimanual examination and cervical inspection:** Pelvic examination is not necessary before initiation of POPs because it does not facilitate detection of conditions for which POPs would be unsafe. Although patients with certain conditions or characteristics should not use (U.S. MEC 4) or generally should not use (U.S. MEC 3) POPs (*1*), none of these conditions are likely to be detected by pelvic examination (*172*). A systematic review identified two case-control studies that compared delayed versus immediate pelvic examination before initiation of hormonal contraceptives, specifically oral contraceptives or DMPA (*23*). No differences in risk factors for cervical neoplasia, incidence of STIs, incidence of abnormal Papanicolaou smears, or incidence of abnormal findings from wet mounts were observed (Level of evidence: II-2 fair, direct).

**Lipids:** Screening for dyslipidemias is not necessary for the safe initiation of POPs because of the low likelihood of clinically significant changes with use of hormonal contraceptives. A systematic review did not identify any evidence regarding outcomes among women who were screened versus not screened with lipid measurement before initiation of hormonal contraceptives (*24*). During 2015–2016 among women aged 20–39 years in the United States, 6.7% had high cholesterol, defined as total serum cholesterol >240 mg/dL (*111*). Studies have reported mixed results about the effects of hormonal methods on lipid levels among both healthy women and women with baseline lipid abnormalities, and the clinical significance of these changes is unclear (*112*–*115*).

**Liver enzymes:** Although patients with hepatocellular carcinoma generally should not use POPs (U.S. MEC 3) (*1*), patients with benign liver tumors, viral hepatitis, or cirrhosis can use (U.S. MEC 1) or generally can use (U.S. MEC 2) POPs; screening for liver disease before initiation of POPs is not necessary because of the low prevalence of these conditions and the high likelihood that patients with liver disease already would have had the condition diagnosed. A systematic review did not identify any evidence regarding outcomes among women who were screened versus not screened with liver enzyme tests before initiation of hormonal contraceptives (*24*). During 2012, among U.S. women, the percentage with liver disease (not further specified) was 1.3% (*116*). During 2013, the incidence of acute hepatitis A, B, or C was ≤1 per 100,000 U.S. population (*117*). During 2002–2011, the incidence of liver cancer among U.S. women was approximately 3.7 per 100,000 population (*118*).

**Clinical breast examination:** Although patients with current breast cancer should not use POPs (U.S. MEC 4) (*1*), screening asymptomatic patients with a clinical breast examination before initiating POPs is not necessary because of the low prevalence of breast cancer among women of reproductive age. A systematic review did not identify any evidence regarding outcomes among women who were screened versus not screened with a clinical breast examination before initiation of hormonal contraceptives (*23*). The incidence of breast cancer among women of reproductive age in the United States is low. During 2020, the incidence of breast cancer among women aged <50 years was approximately 45.9 per 100,000 women (*119*).

**Other screening:** Patients with hypertension, diabetes, iron-deficiency anemia, thrombophilia, cervical intraepithelial neoplasia, cervical cancer, STIs, or HIV infection can use (U.S. MEC 1) or generally can use (U.S. MEC 2) POPs (*1*). Therefore, screening for these conditions is not necessary for the safe initiation of POPs.

### Number of Pill Packs that Should Be Provided at Initial and Return Visits

- At the initial and return visit, provide or prescribe up to a 1-year supply of POPs (e.g., 13 28-day pill packs), depending on the patient’s preferences and anticipated use.
- A patient should be able to obtain POPs easily in the amount and at the time they need them.

**Comments and Evidence Summary.** The more pill packs provided up to 13 cycles, the higher the continuation rates. Restricting the number of pill packs distributed or prescribed can be a barrier for patients who want to continue POP use and might increase risk for pregnancy.

A systematic review of the evidence suggested that providing a greater number of pill packs was associated with increased continuation (*20*). Studies that compared provision of one versus 12 packs, one versus 12 or 13 packs, or three versus seven packs found increased continuation of pill use among women provided with more pill packs (*287*–*289*). However, one study found no difference in continuation when patients were provided one and then three packs versus four packs all at once (*290*). In addition to continuation, a greater number of pill packs provided was associated with fewer pregnancy tests, fewer pregnancies, and lower cost per client. However, a greater number of pill packs (13 packs versus three packs) also was associated with increased pill wastage in one study (*288*) (Level of evidence: I to II-2, fair, direct).

### Routine Follow-Up After POP Initiation

These recommendations address when routine follow-up is recommended for safe and effective continued use of contraception for healthy patients. The recommendations refer to general situations and might vary for different users and different situations. Specific populations who might benefit from more frequent follow-up visits include adolescents, those with certain medical conditions or characteristics, and those with multiple medical conditions.

- Advise the patient that they may contact their provider at any time to discuss side effects or other problems or if they want to change the method being used. No routine follow-up visit is required.
- At other routine visits, health care providers seeing POP users should do the following:
  º Assess the patient’s satisfaction with their contraceptive method and whether they have any concerns about method use.
  º Assess any changes in health status, including medications, that would change the appropriateness of POPs for safe and effective continued use on the basis of U.S. MEC (e.g., category 3 and 4 conditions and characteristics) (*1*).
  º Consider assessing weight changes and discussing concerns about any changes in weight and whether changes might be related to use of the contraceptive method.

**Comments and Evidence Summary.** No evidence was found regarding whether a routine follow-up visit after initiating POPs improves correct or continued use.

### Missed POPs

#### Norethindrone or Norgestrel POPs

**FIGURE 5:**
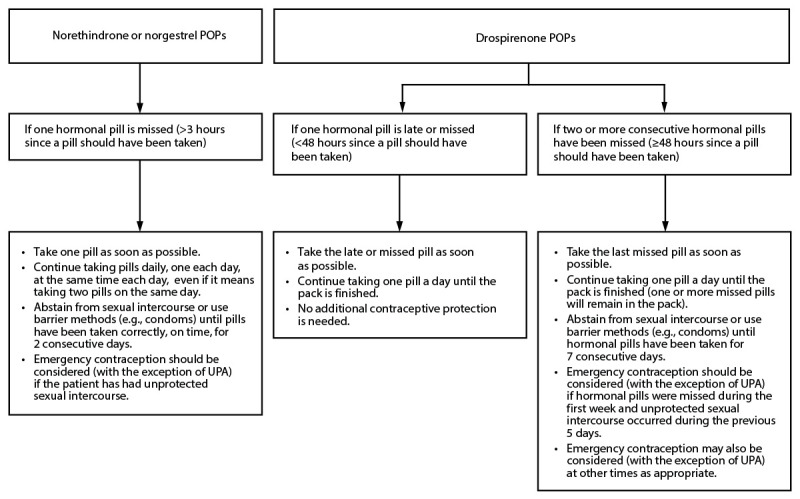
Recommended actions after late or missed progestin-only pills **Abbreviations:** POP = progestin-only pill; UPA = ulipristal acetate.

For norethindrone or norgestrel POPs, a dose is considered missed if it has been >3 hours since it should have been taken. Recommendations are provided for missed norethindrone or norgestrel POPs (Figure 5).

**Comments and Evidence Summary.** Inconsistent or incorrect use of oral contraceptive pills is a major reason for oral contraceptive failure. Unlike COCs, which inhibit ovulation as the primary mechanism of action, norethindrone or norgestrel POPs inhibit ovulation in about half of cycles, although this rate varies widely by person (*333*). Peak serum steroid levels are reached about 2 hours after administration, followed by rapid distribution and elimination, such that by 24 hours after administration, serum steroid levels are near baseline (*333*). Therefore, taking norethindrone or norgestrel POPs at approximately the same time each day is important. An estimated 48 hours of norethindrone or norgestrel POP use was deemed necessary to achieve the contraceptive effects on cervical mucus (*333*). For patients who frequently miss norethindrone or norgestrel POPs, explore patient goals, consider offering counseling on alternative contraceptive methods, and initiate another method if it is desired. No evidence was found regarding the effects of missed norethindrone or norgestrel POPs available in the United States on measures of contraceptive effectiveness including pregnancy, follicular development, hormone levels, or cervical mucus quality.

#### DRSP POPs

For the following recommendations, a dose is considered late when <24 hours have elapsed since the dose should have been taken. A dose is considered missed if ≥24 hours have elapsed since the dose should have been taken. For example, if a DRSP POP was supposed to have been taken on Monday at 9:00 a.m. and is taken at 11:00 a.m., the pill is late; however, by Tuesday morning at 11:00 a.m., Monday’s 9:00 a.m. pill has been missed and Tuesday’s 9:00 a.m. pill is late. For DRSP POPs, the recommendations only apply to late or missed hormonally active pills and not to placebo pills. Recommendations are provided for late or missed DRSP POPs (Figure 5).

**Comments and Evidence Summary.** Inconsistent or incorrect use of oral contraceptives is a major cause of oral contraceptive failure. Unlike norethindrone and norgestrel POPs, the primary mechanism of contraceptive effectiveness of DRSP POPs is ovulation inhibition. In a study of 27 patients receiving DRSP POPs in a regimen of 24 days of active pills/4 days of placebo pills, no subjects met normal ovulatory criteria over two treatment cycles (*334*). Earliest time to ovulation resumption was day 9 after two 24/4 cycles were completed (day 13 after the last hormonally active pill was taken); mean time to ovulation after two 24/4 cycles were completed was 13.6±3.8 days (*334*). In an RCT of 127 participants, participants purposefully missed pills (22–25 hour delay) on days 3, 6, 11, and 22 in either treatment cycle one or two of the 24/4 regimen (*335*). Escape ovulation occurred in only one person over the two treatment cycles (ovulation incidence 0.8%; 95% CI 0%–4.4%) (*335*). DRSP has a half-life of approximately 30 hours with near-complete elimination by 10 days (*336*). For patients who frequently miss DRSP POPs, explore patient goals, consider offering counseling on alternative contraceptive methods, and initiate another method if it is desired.

### Vomiting or Diarrhea (for any Reason or Duration) that Occurs Within 3 Hours After Taking a Pill

#### Norethindrone or Norgestrel POPs

- Take another pill as soon as possible (if possible, despite discomfort).
- Continue taking pills daily, one each day, at the same time each day.
- Abstain from sexual intercourse or use barrier methods (e.g., condoms) until 2 days after vomiting or diarrhea has resolved.
- Emergency contraception should be considered (with the exception of UPA) if the patient has had unprotected sexual intercourse.

#### DRSP POPs

- Take another pill as soon as possible (if possible, despite discomfort).
- Continue taking pills daily, one each day, at the same time each day.
- If vomiting or diarrhea continues for >24 hours, then abstain from sexual intercourse or use barrier methods (e.g., condoms) for 7 days after vomiting or diarrhea has resolved.
- Emergency contraception should be considered (with the exception of UPA) if the patient has had unprotected sexual intercourse.

**Comments and Evidence Summary.** Theoretically, the contraceptive effectiveness of all POPs might be decreased because of vomiting or severe diarrhea. Because of the lack of evidence to address this question, these recommendations are based on the recommendations for missed POPs. No evidence was found regarding the effects of vomiting or diarrhea on measures of contraceptive effectiveness, including pregnancy, follicular development, hormone levels, or cervical mucus quality.

## Standard Days Method

SDM is based on fertility awareness; users must avoid unprotected sexual intercourse on days 8–19 of the menstrual cycle (*337*). Approximately 13 out of 100 SDM users become pregnant in the first year with typical use (*28*). SDM is reversible and can be used by patients of all ages. SDM does not protect against STIs, including HIV infection, and patients using SDM should be counseled that consistent and correct use of external (male) latex condoms reduces the risk for STIs, including HIV infection (*31*). Use of internal (female) condoms can provide protection from STIs, including HIV infection, although data are limited (*31*). Patients also should be counseled that PrEP, when taken as prescribed, is highly effective for preventing HIV infection (*32*).

### Use of SDM with Various Durations of the Menstrual Cycle

#### Menstrual Cycles of 26–32 Days

- The patient may use the method.
- Provide a barrier method (e.g., condoms) for protection on days 8–19, if they want one.
- If the patient has unprotected sexual intercourse during days 8–19, consider the use of emergency contraception if appropriate.

#### Two or More Cycles of <26 or >32 Days Within Any 1 Year of SDM Use

- Advise the patient that the method might not be appropriate for them because of a higher risk for pregnancy. Help them consider another method.

**Comments and Evidence Summary.** The probability of pregnancy when using SDM is increased when the menstrual cycle is outside the range of 26–32 days, even if unprotected sexual intercourse is avoided on days 8–19. A study examining 7,600 menstrual cycles, including information on cycle length and signs of ovulation, concluded that the theoretical effectiveness of SDM is greatest for women with cycles of 26–32 days, that the method is still effective for women who occasionally have a cycle outside this range, and that the method is less effective for women who consistently have cycles outside this range. Information from daily hormonal measurements demonstrates that the timing of the 6-day fertile window varies greatly, even among women with regular cycles (*38*,*338*,*339*).

## Emergency Contraception

Emergency contraception consists of methods that persons can use after sexual intercourse to prevent pregnancy. Emergency contraception methods have varying ranges of effectiveness depending on the method and timing of administration. Four options are available in the United States: the Cu-IUD and three types of ECPs. Emergency contraception does not protect against STIs, including HIV infection, and patients using emergency contraception should be counseled that consistent and correct use of external (male) latex condoms reduces the risk for STIs, including HIV infection (*31*). Use of internal (female) condoms can provide protection from STIs, including HIV infection, although data are limited (*31*). Patients also should be counseled that PrEP, when taken as prescribed, is highly effective for preventing HIV infection (*32*).

### Types of Emergency Contraception

#### Intrauterine Device

- Cu-IUD

#### Emergency Contraceptive Pills

- UPA in a single dose (30 mg)
- LNG in a single dose (1.5 mg) or as a split dose (1 dose of 0.75 mg of LNG followed by a second dose of 0.75 mg of LNG 12 hours later)
- Combined estrogen and progestin in 2 doses (Yuzpe regimen: 1 dose of 100 *µ*g of EE plus 0.50 mg of LNG followed by a second dose of 100 *µ*g of EE plus 0.50 mg of LNG 12 hours later)

### Initiation of Emergency Contraception

#### Timing

##### Cu-IUD

- The Cu-IUD may be placed within 5 days of the first act of unprotected sexual intercourse as emergency contraception.
- In addition, when the day of ovulation can be estimated, the Cu-IUD may be placed >5 days after sexual intercourse, as long as placement does not occur >5 days after ovulation.

##### ECPs

- ECPs should be taken as soon as possible within 5 days of unprotected sexual intercourse.

**Comments and Evidence Summary.** Cu-IUDs are highly effective as emergency contraception (*340*) and can be continued as regular contraception. UPA and LNG ECPs have similar effectiveness when taken within 3 days after unprotected sexual intercourse; however, UPA has been observed to be more effective than the LNG formulation 3–5 days after unprotected sexual intercourse (*341*). The combined estrogen and progestin regimen is less effective than UPA or LNG and also is associated with more frequent occurrence of side effects (nausea and vomiting) (*342*). The LNG formulation might be less effective than UPA among women with obesity (*343*).

Two studies of UPA use found consistent decreases in pregnancy rates when administered within 120 hours of unprotected sexual intercourse (*341*,*344*). Five studies found that the LNG and combined regimens decreased risk for pregnancy through the fifth day after unprotected sexual intercourse; however, rates of pregnancy were slightly higher when ECPs were taken after 3 days (*345*–*349*). A meta-analysis of LNG ECPs found that pregnancy rates were low when administered within 4 days after unprotected sexual intercourse but increased at 4–5 days (*350*) (Level of evidence: I to II-2, good to poor, direct).

### Advance Provision of ECPs

- An advance supply of ECPs may be provided so that ECPs will be available when needed and can be taken as soon as possible after unprotected sexual intercourse.

**Comments and Evidence Summary.** A systematic review identified 17 studies that reported on safety or effectiveness of advance ECPs in adult or adolescent women (*351*). Any use of ECPs was two to seven times greater among women who received an advance supply of ECPs. However, a summary estimate (RR = 0.9; 95% CI = 0.7–1.2) of four RCTs did not indicate a significant reduction in pregnancies at 12 months with advance provision of ECPs. In the majority of studies among adults or adolescents, patterns of regular contraceptive use, pregnancy rates, and incidence of STIs did not vary between those who received advance ECPs and those who did not. Although available evidence supports the safety of advance provision of ECPs, effectiveness of advance provision of ECPs in reducing pregnancy rates at the population level has not been demonstrated (Level of evidence: I to II-3, good to poor, direct).

### Use of Regular Contraception After ECPs

#### Ulipristal Acetate

- Advise the patient to start or resume hormonal contraception no sooner than 5 days after use of UPA and provide or prescribe the regular contraceptive method as needed. For methods requiring a visit to a health care provider, such as provider-administered DMPA, implants, and IUDs, starting the method at the time of UPA use may be considered; the risk that the regular contraceptive method might decrease the effectiveness of UPA must be weighed against the risk of not starting a regular hormonal contraceptive method.
- The patient needs to abstain from sexual intercourse or use barrier methods (e.g., condoms) for the next 7 days after starting or resuming regular contraception or until their next menses, whichever comes first.
- Any nonhormonal contraceptive method may be started immediately after the use of UPA.
- Advise the patient to have a pregnancy test if they do not have a withdrawal bleed within 3 weeks.

#### Levonorgestrel and Combined Estrogen and Progestin ECPs

- Any regular contraceptive method may be started or resumed immediately after the use of LNG or combined estrogen and progestin ECPs.
- The patient needs to abstain from sexual intercourse or use barrier methods (e.g., condoms) for 7 days.
- Advise the patient to have a pregnancy test if they do not have a withdrawal bleed within 3 weeks.

**Comments and Evidence Summary.** Because of the antiprogestin properties of UPA, concern exists that starting or resuming progestin-containing regular contraception around the same time as UPA administration might decrease the effectiveness of UPA or the regular contraceptive method. Therefore, the initiation or resumption of regular hormonal contraception after UPA use involves consideration of the risk for pregnancy if UPA fails and the risk for pregnancy if regular contraception use is delayed until the subsequent menstrual cycle. A health care provider can provide or prescribe pills, the patch, or the ring for a patient to start no sooner than 5 days after use of UPA. For methods requiring a visit to a health care provider, such as provider-administered DMPA, implants, and IUDs, starting the method at the time of UPA use may be considered; the risk that the regular contraceptive method might decrease the effectiveness of UPA must be weighed against the risk of not starting a regular hormonal contraceptive method.

No concern exists that administering LNG or combined estrogen and progestin ECPs concurrently with systemic hormonal contraception decreases the effectiveness of either emergency or regular contraceptive methods because these formulations do not have anti-progestin properties like UPA. If starting or resuming regular contraception after the next menstrual bleeding after ECP use, the cycle in which ECPs are used might be shortened, prolonged, or involve irregular bleeding.

A systematic review identified four studies that assessed contraceptive effectiveness (as measured by ovarian activity) of UPA or regular hormonal contraception, when the two drugs were taken at approximately the same time (*352*–*355*) (Supplementary Appendix, https://stacks.cdc.gov/view/cdc/156517). Two studies found no differences in ovarian activity when starting oral contraceptives (one study used COCs and one study used desogestrel POPs) after UPA administration compared with starting oral contraceptives after placebo, suggesting that UPA did not affect the ability of the oral contraceptive to inhibit ovulation (ovulations: 33% of UPA+COC group versus 32% of placebo+COC group; 45% of UPA+POP group versus 38% of placebo+POP group) (*353**,**354*). However, two studies observed higher proportions of ovulation when starting oral contraceptives within 5 days of UPA administration compared with delayed or no use of hormonal contraception, suggesting that oral contraceptive use within 5 days of UPA administration decreased the ability of UPA to delay ovulation (ovulations: 27% of COC+UPA group versus 3% of UPA only group; 45% of POP+UPA group versus 3% of placebo+UPA group) (*353**,**355*). One study examined the risk for ovulation after UPA was taken after missing three COC pills on days 5–7 of the cycle followed by immediate versus delayed resumption of COCs. Whereas no ovulations were observed within the first 5 days after UPA administration, there was a greater risk of ovulation >5 days after UPA administration among those who delayed COC resumption compared with those who resumed immediately (ovulations: four events in delayed group versus zero in immediate group [odds ratio = 7.78; 95% CI = 1.38–43.95]) (*352*). The evidence is limited to specific contraceptive formulations and study populations (e.g., limited age and BMI distributions and normal menstrual cycles) (Certainty of evidence: very low to moderate).

### Prevention and Management of Nausea and Vomiting with ECP Use

#### Nausea and Vomiting

- LNG and UPA ECPs cause less nausea and vomiting than combined estrogen and progestin ECPs.
- Routine use of antiemetics before taking ECPs is not recommended. Pretreatment with antiemetics may be considered depending on availability and clinical judgment.

#### Vomiting Within 3 Hours of Taking ECPs

- Another dose of ECP should be taken as soon as possible. Use of an antiemetic should be considered.

**Comments and Evidence Summary.** Many patients do not experience nausea or vomiting when taking ECPs, and predicting which patients will experience nausea or vomiting is difficult. Although routine use of antiemetics before taking ECPs is not recommended, antiemetics are effective in certain patients and can be offered when appropriate. Health care providers who are deciding whether to offer antiemetics to patients taking ECPs should consider the following: 1) patients taking combined estrogen and progestin ECPs are more likely to experience nausea and vomiting than those who take LNG or UPA ECPs, 2) evidence indicates that antiemetics reduce the occurrence of nausea and vomiting in patients taking combined estrogen and progestin ECPs, and 3) patients who take antiemetics might experience other side effects from the antiemetics.

A systematic review examined incidence of nausea and vomiting with different ECP regimens and effectiveness of antinausea drugs in reducing nausea and vomiting with ECP use (*356*). The LNG regimen was associated with significantly less nausea than a nonstandard dose of UPA (50 mg) and the standard combined estrogen and progestin regimen (*357*–*359*). Use of the split-dose LNG demonstrated no differences in nausea and vomiting compared with the single-dose LNG (*345*,*347*,*349*,*360*) (Level of evidence: I, good-fair, indirect). Two trials of antinausea drugs (meclizine and metoclopramide), taken before combined estrogen and progestin ECPs, reduced the severity of nausea (*361*,*362*). Significantly less vomiting occurred with meclizine but not metoclopramide (Level of evidence: I, good-fair, direct). No direct evidence was found regarding the effects of vomiting after taking ECPs.

## Permanent Contraception

Tubal surgery (including laparoscopic and abdominal approaches) and vasectomy are methods of permanent contraception that are available in the United States. Approximately 0.5 out of 100 tubal surgery users will become pregnant in the first year of typical use; the typical failure rate for vasectomy is 0.15 per 100 users in the first year of typical use (*28*). Because these methods are intended to be irreversible, patients should be appropriately counseled about the permanency of these methods and the availability of highly effective, long-acting reversible methods of contraception. Permanent contraception does not protect against STIs, including HIV infection, and patients using permanent contraception should be counseled that consistent and correct use of external (male) latex condoms reduces the risk for STIs, including HIV infection (*31*). Use of internal (female) condoms can provide protection from STIs, including HIV infection, although data are limited (*31*). Patients also should be counseled that PrEP, when taken as prescribed, is highly effective for preventing HIV infection (*32*).

### When Tubal Surgery Is Reliable for Contraception

- A patient may rely on permanent contraception immediately after laparoscopic and abdominal approaches. No additional contraceptive protection is needed.

**Comments and Evidence Summary.** Pregnancy risk with at least 10 years of follow-up has been studied among women who received laparoscopic and abdominal sterilizations (*363*,*364*). Although these methods are highly effective, pregnancies can occur many years after the procedure, and the risk for pregnancy is higher among younger women (*364*,*365*).

### When Vasectomy Is Reliable for Contraception and Other Postprocedure Recommendations

- Semen analysis should be performed 8–16 weeks after a vasectomy to ensure the procedure was successful.
- The patient should be advised that they should abstain from sexual intercourse or use barrier methods (e.g., condoms) until they have confirmation of vasectomy success by postvasectomy semen analysis.
- The patient should refrain from ejaculation for approximately 1 week after the vasectomy to allow for healing of surgical sites and, after certain methods of vasectomy, occlusion of the vas.

**Comments and Evidence Summary.** The Vasectomy Guideline Panel of the American Urological Association performed a systematic review of key issues concerning the practice of vasectomy (*366*). All English-language publications on vasectomy published during 1949–2011 were reviewed. For more information, see the American Urological Association’s *Vasectomy: AUA Guideline* (https://www.auanet.org/guidelines-and-quality/guidelines/vasectomy-guideline).

Motile sperm disappear within a few weeks after vasectomy (*367*–*370*). The time to azoospermia varies widely in different studies; however, by 12 weeks after the vasectomy, 80% of men have azoospermia, and almost all others have rare nonmotile sperm (defined as ≤100,000 nonmotile sperm per mL) (*366*). The number of ejaculations after vasectomy is not a reliable indicator of when azoospermia or rare nonmotile sperm will be achieved (*366*). When azoospermia or rare nonmotile sperm has been achieved, patients can rely on the vasectomy for contraception, although not with 100% certainty. The risk for pregnancy after a man has achieved postvasectomy azoospermia is approximately one in 2,000 (*371*–*375*).

A median of 78% (range = 33%–100% across studies) of men return for a single postvasectomy semen analysis (*366*). In the largest cohorts that appear typical of North American vasectomy practice, approximately two thirds of men (55%–71%) return for at least one postvasectomy semen analysis (*371*,*376*–*380*). Assigning men an appointment after their vasectomy might improve compliance with follow-up (*381*).

## When Contraceptive Protection Is No Longer Needed

- Contraceptive protection is still needed for patients aged >44 years who want to avoid becoming pregnant.

**Comments and Evidence Summary.** The age at which a person is no longer at risk for becoming pregnant is not known. Although uncommon, spontaneous pregnancies occur among persons aged >44 years. Both the American College of Obstetricians and Gynecologists and the North American Menopause Society recommend that women continue contraceptive use until menopause or age 50–55 years (*382*,*383*). The median age of menopause is approximately 51 years in North America (*382*) but can vary from 40 to 60 years (*384*). The median age of definitive loss of natural fertility is 41 years but can range up to 51 years (*385*,*386*). No reliable laboratory tests are available to confirm definitive loss of fertility in a woman; the assessment of follicle-stimulating hormone levels to determine when a woman is no longer fertile might not be accurate (*382*).

Health care providers should consider the risks for becoming pregnant in a patient of advanced reproductive age, as well as any risks of continuing contraception until menopause. Pregnancies among women of advanced reproductive age are at higher risk for maternal complications (e.g., hemorrhage, venous thromboembolism, and death) and fetal complications (e.g., spontaneous abortion, stillbirth, and congenital anomalies) (*387*–*389*). Risks associated with continuing contraception, in particular risks for acute cardiovascular events (venous thromboembolism, myocardial infarction, or stroke) or breast cancer, also are important to consider. U.S. MEC states that on the basis of age alone, patients of any age can use (U.S. MEC 1) or generally can use (U.S. MEC 2) IUDs and hormonal contraception (*1*). However, patients of advanced reproductive age might have chronic conditions or other risk factors that might render use of hormonal contraceptive methods unsafe; U.S. MEC might be helpful in guiding the safe use of contraceptives in these patients (*1*).

In two studies, the incidence of venous thromboembolism was higher among oral contraceptive users aged 45–49 years compared with younger oral contraceptive users (*390*–*392*); however, an interaction between hormonal contraception and increased age compared with baseline risk was not demonstrated (*390*,*391*) or was not examined (*392*). The relative risk for myocardial infarction was higher among all oral contraceptive users than among nonusers, although a trend of increased relative risk with increasing age was not demonstrated (*393*,*394*). No studies were found regarding the risk for stroke in COC users aged 45–49 years (Level of evidence: II-2, good to poor, direct).

A pooled analysis by the Collaborative Group on Hormonal Factors and Breast Cancer in 1996 (*395*) found small increased relative risks for breast cancer among women aged ≥45 years whose last use of CHCs was <5 years previously and for those whose last use of CHCs was 5–9 years previously. Seven more recent studies suggested small but nonsignificant increased relative risks for breast carcinoma in situ or breast cancer among women who had used oral contraceptives or DMPA when they were aged ≥40 years compared with those who had never used either method (*396*–*402*) (Level of evidence: II-2, fair, direct).

## Conclusion

U.S. SPR can support health care providers in removing unnecessary medical barriers, expanding equitable access to the full range of contraceptive methods, and providing person-centered counseling and contraceptive services in a noncoercive manner that supports a person’s values, goals, and reproductive autonomy. Most patients may start most contraceptive methods at any time, and few examinations or tests, if any, are needed before starting a contraceptive method. Routine follow-up for most patients includes assessment of their satisfaction with the contraceptive method, concerns about method use, and changes in health status or medications that could affect medical eligibility for continued use of the method. Because changes in bleeding patterns are one of the major reasons for discontinuation of contraception, recommendations are provided for the management of bleeding irregularities with various contraceptive methods. ECPs and emergency use of the Cu-IUD are important options, and recommendations for using these methods, as well as starting regular contraception after use of emergency contraception, are provided. Permanent contraception is highly effective for persons who have completed childbearing or do not wish to have children; for persons undergoing vasectomy, additional contraceptive protection is needed until the success of the procedure can be confirmed.

CDC is committed to working with partners at the Federal, national, and local levels to disseminate, implement, and evaluate U.S. SPR recommendations so that the information reaches health care providers. Strategies for dissemination and implementation include collaborating with other Federal agencies and professional and service organizations to widely distribute the recommendations through presentations, electronic distribution, newsletters, and other publications; development of provider tools and job aids to assist providers in implementing the new recommendations; and training activities for students, as well as for continuing education. Finally, CDC will continually monitor new scientific evidence and update these recommendations as warranted by new evidence. Updates to the recommendations, as well as provider tools and other resources, are available on the CDC website (https://www.cdc.gov/contraception/hcp/contraceptive-guidance).

## Figures and Tables

**FIGURE E1:**
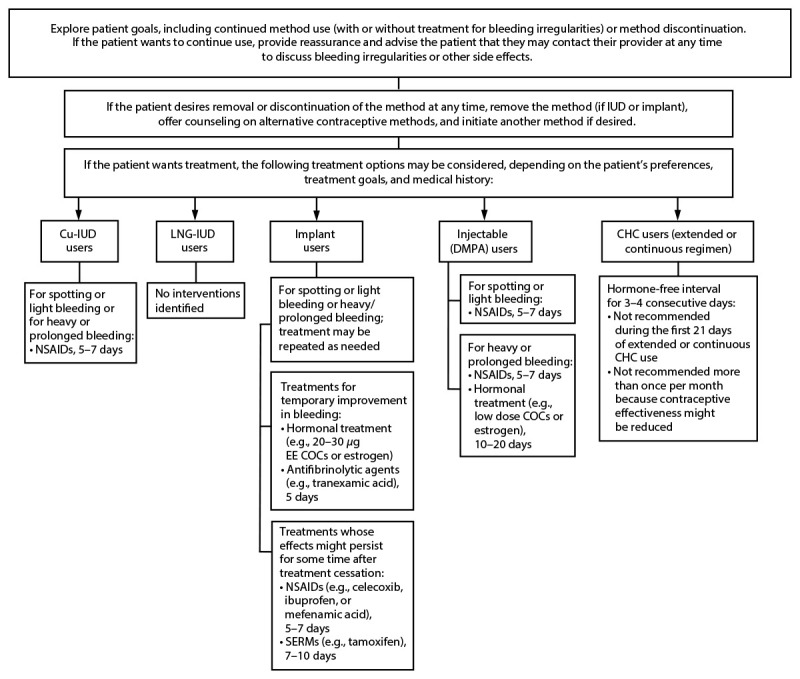
Management of bleeding irregularities while using contraception* **Abbreviations:** CHC = combined hormonal contraceptive; COC = combined oral contraceptive; Cu-IUD = copper intrauterine device; DMPA = depot medroxyprogesterone acetate; EE = ethinyl estradiol; LNG-IUD = levonorgestrel intrauterine device; NSAID = nonsteroidal anti-inflammatory drug; SERM = selective estrogen receptor modulator. * If clinically indicated, consider an underlying health condition, such as interactions with other medications, sexually transmitted infections, pregnancy, thyroid disorders, or new pathologic uterine conditions (e.g., polyps or fibroids). If an underlying health condition is found, treat the condition or refer for care.

**FIGURE F1:**
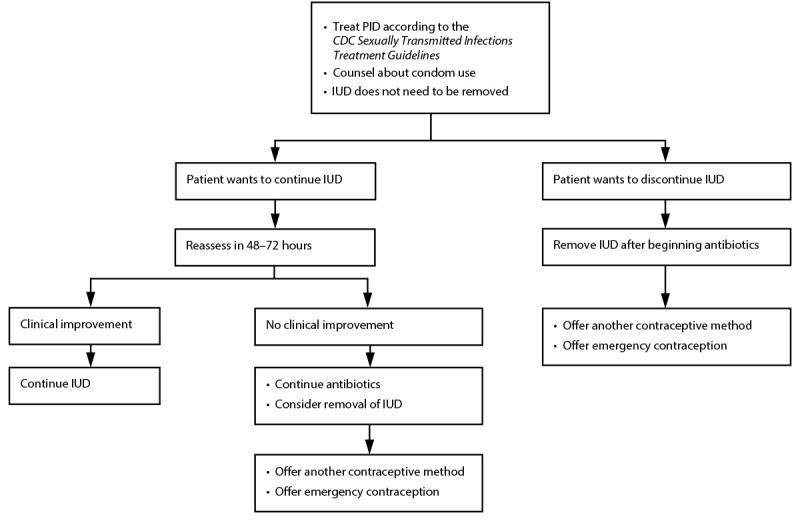
Management of intrauterine devices when users of copper intrauterine devices or levonorgestrel intrauterine devices are found to have pelvic inflammatory disease* **Abbreviations:** IUD = intrauterine device; PID = pelvic inflammatory disease. * Refer to *CDC Sexually Transmitted Infections Treatment Guidelines* (https://www.cdc.gov/std/treatment-guidelines/default.htm) for information on PID diagnostic considerations and treatment regimens.

## Footnotes

* Indicates a substantive modification from the 2016 U.S. SPR.

## Appendix A Summary of Classifications for *U.S. Medical Eligibility Criteria for Contraceptive Use, 2024*

Health care providers can use the summary table as a quick reference guide to the classifications for hormonal contraceptive methods and intrauterine contraception to compare classifications across these methods (Box A1) (Table A1). For complete guidance, see *U.S. Medical Eligibility Criteria for Contraceptive Use, 2024* (U.S. MEC) (*1*) for clarifications to the numeric categories, as well as for summaries of the evidence and additional comments. Hormonal contraceptives and intrauterine devices do not protect against sexually transmitted infections (STIs), including HIV infection, and persons using these methods should be counseled that consistent and correct use of external (male) latex condoms reduces the risk for STIs, including HIV infection (*2*). Use of internal (female) condoms can provide protection from transmission of STIs, although data are limited (*2*). Patients also should be counseled that pre-exposure prophylaxis, when taken as prescribed, is highly effective for preventing HIV infection (*3*).

BOX A1 Categories for classifying hormonal contraceptives and intrauterine devices U.S. MEC 1 = A condition for which there is no restriction for the use of the contraceptive method U.S. MEC 2 = A condition for which the advantages of using the method generally outweigh the theoretical or proven risks U.S. MEC 3 = A condition for which the theoretical or proven risks usually outweigh the advantages of using the method U.S. MEC 4 = A condition that represents an unacceptable health risk if the contraceptive method is used **Abbreviation:** U.S. MEC = *U.S. Medical Eligibility Criteria for Contraceptive Use.*

**TABLE A1:** Summary of classifications for hormonal contraceptive methods and intrauterine devices

Condition	Cu-IUD		LNG-IUD		Implant		DMPA		POP		CHC	
**Personal Characteristics and Reproductive History**												
**Pregnancy**	4*		4*		NA*		NA*		NA*		NA*	
**Age**	Menarche to <20 years: 2		Menarche to <20 years: 2		Menarche to <18 years: 1		Menarche to <18 years: 2		Menarche to <18 years: 1		Menarche to <40 years: 1	
	≥20 years: 1		≥20 years: 1		18–45 years: 1		18–45 years: 1		18–45 years: 1		≥40 years: 2	
					>45 years: 1		>45 years: 2		>45 years: 1			
**Parity**												
a. Nulliparous	2		2		1		1		1		1	
b. Parous	1		1		1		1		1		1	
**Breastfeeding**												
a. <21 days postpartum	—		—		2*		2*		2*		4*	
b. 21 to <30 days postpartum												
i. With other risk factors for VTE (e.g., age ≥35 years, previous VTE, thrombophilia, immobility, transfusion at delivery, peripartum cardiomyopathy, BMI ≥30 kg/m^2^, postpartum hemorrhage, postcesarean delivery, preeclampsia, or smoking)	—		—		2*		2*		2*		3*	
ii. Without other risk factors for VTE	—		—		2*		2*		2*		3*	
c. 30–42 days postpartum												
i. With other risk factors for VTE (e.g., age ≥35 years, previous VTE, thrombophilia, immobility, transfusion at delivery, peripartum cardiomyopathy, BMI ≥30 kg/m^2^, postpartum hemorrhage, postcesarean delivery, preeclampsia, or smoking)	—		—		1*		2*		1*		3*	
ii. Without other risk factors for VTE	—		—		1*		1*		1*		2*	
d. >42 days postpartum	—		—		1*		1*		1*		2*	
**Postpartum** (nonbreastfeeding)												
a. <21 days postpartum	—		—		1		2		1		4	
b. 21–42 days postpartum												
i. With other risk factors for VTE (e.g., age ≥35 years, previous VTE, thrombophilia, immobility, transfusion at delivery, peripartum cardiomyopathy, BMI ≥30 kg/m^2^, postpartum hemorrhage, postcesarean delivery, preeclampsia, or smoking)	—		—		1		2		1		3*	
ii. Without other risk factors for VTE	—		—		1		1		1		2	
c. >42 days postpartum	—		—		1		1		1		1	
**Postpartum** (including cesarean delivery, breastfeeding, or nonbreastfeeding)												
a. <10 minutes after delivery of the placenta	2*		2*		—		—		—		—	
b. 10 minutes after delivery of the placenta to <4 weeks	2*		2*		—		—		—		—	
c. ≥4 weeks	1*		1*		—		—		—		—	
d. Postpartum sepsis	4		4		—		—		—		—	
**Postabortion (spontaneous or induced)**												
a. First trimester abortion												
i. Procedural (surgical)	1*		1*		1*		1*		1*		1*	
ii. Medication	1*		1*		1*		1/2*		1*		1*	
iii. Spontaneous abortion with no intervention	1*		1*		1*		1*		1*		1*	
b. Second trimester abortion												
i. Procedural (surgical)	2*		2*		1*		1*		1*		1*	
ii. Medication	2*		2*		1*		1*		1*		1*	
iii. Spontaneous abortion with no intervention	2*		2*		1*		1*		1*		1*	
c. Immediate postseptic abortion	4		4		1*		1*		1*		1*	
**Past ectopic pregnancy**	1		1		1		1		2		1	
**History of pelvic surgery** (see recommendations for Postpartum [including cesarean delivery])	1		1		1		1		1		1	
**Smoking**												
a. Age <35 years	1		1		1		1		1		2	
b. Age ≥35 years												
i. <15 cigarettes per day	1		1		1		1		1		3	
ii. ≥15 cigarettes per day	1		1		1		1		1		4	
**Obesity**												
a. BMI ≥30 kg/m^2^	1		1		1		1		1		2*	
b. Menarche to <18 years and BMI ≥30 kg/m^2^	1		1		1		2		1		2*	
**History of bariatric surgery** This condition is associated with increased risk for adverse health events as a result of pregnancy.												
a. Restrictive procedures: decrease storage capacity of the stomach (vertical banded gastroplasty, laparoscopic adjustable gastric band, or laparoscopic sleeve gastrectomy)	1		1		1		1		1		1	
b. Malabsorptive procedures: decrease absorption of nutrients and calories by shortening the functional length of the small intestine (Roux-en-Y gastric bypass or biliopancreatic diversion)	1		1		1		1		3		COCs: 3 Patch and ring: 1	
**Surgery**												
a. Minor surgery without immobilization	1		1		1		1		1		1	
b. Major surgery												
i. Without prolonged immobilization	1		1		1		1		1		2	
ii. With prolonged immobilization	1		1		1		2		1		4	
**Cardiovascular Disease**												
**Multiple risk factors for atherosclerotic cardiovascular disease** (e.g., older age, smoking, diabetes, hypertension, low HDL, high LDL, or high triglyceride levels)	1		2		2*		3*		2*		3/4*	
**Hypertension** Systolic blood pressure ≥160 mm Hg or diastolic blood pressure ≥100 mm Hg are associated with increased risk for adverse health events as a result of pregnancy.												
a. Adequately controlled hypertension	1*		1*		1*		2*		1*		3*	
b. Elevated blood pressure levels (properly taken measurements)												
i. Systolic 140–159 mm Hg or diastolic 90–99 mm Hg	1*		1*		1*		2*		1*		3*	
ii. Systolic ≥160 mm Hg or diastolic ≥100 mm Hg	1*		2*		2*		3*		2*		4*	
c. Vascular disease	1*		2*		2*		3*		2*		4*	
**History of high blood pressure during pregnancy** (when current blood pressure is measurable and normal)	1		1		1		1		1		2	
**Deep venous thrombosis/Pulmonary embolism** This condition is associated with increased risk for adverse health events as a result of pregnancy.												
a. Current or history of DVT/PE, receiving anticoagulant therapy (therapeutic dose) (e.g., acute DVT/PE or long-term therapeutic dose)	2*		2*		2*		2*		2*		3*	
b. History of DVT/PE, receiving anticoagulant therapy (prophylactic dose)												
i. Higher risk for recurrent DVT/PE (one or more risk factors)	2*		2*		2*		3*		2*		4*	
• Thrombophilia (e.g., factor V Leiden mutation; prothrombin gene mutation; protein S, protein C, and antithrombin deficiencies; or antiphospholipid syndrome) • Active cancer (metastatic, receiving therapy, or within 6 months after clinical remission), excluding nonmelanoma skin cancer • History of recurrent DVT/PE												
ii. Lower risk for recurrent DVT/PE (no risk factors)	2*		2*		2*		2*		2*		3*	
c. History of DVT/PE, not receiving anticoagulant therapy												
i. Higher risk for recurrent DVT/PE (one or more risk factors	1		2		2		3		2		4	
• History of estrogen-associated DVT/PE • Pregnancy-associated DVT/PE • Idiopathic DVT/PE • Thrombophilia (e.g., factor V Leiden mutation; prothrombin gene mutation; protein S, protein C, and antithrombin deficiencies; or antiphospholipid syndrome) • Active cancer (metastatic, receiving therapy, or within 6 months after clinical remission), excluding nonmelanoma skin cancer • History of recurrent DVT/PE												
ii. Lower risk for recurrent DVT/PE (no risk factors)	1		2		2		2		2		3	
d. Family history (first-degree relatives)	1		1		1		1		1		2	
**Thrombophilia** (e.g., factor V Leiden mutation; prothrombin gene mutation; protein S, protein C, and antithrombin deficiencies; or antiphospholipid syndrome) This condition is associated with increased risk for adverse health events as a result of pregnancy.	1*		2*		2*		3*		2*		4*	
**Superficial venous disorders**												
a. Varicose veins	1		1		1		1		1		1	
b. Superficial venous thrombosis (acute or history)	1		1		1		2		1		3*	
**Current and history of ischemic heart disease** This condition is associated with increased risk for adverse health events as a result of pregnancy.	1		Initiation	Continuation	Initiation	Continuation	3		Initiation	Continuation	4	
			2	3	2	3			2	3		
**Stroke** (history of cerebrovascular accident) This condition is associated with increased risk for adverse health events as a result of pregnancy.	1		2		Initiation	Continuation	3		Initiation	Continuation	4	
					2	3			2	3		
**Valvular heart disease** Complicated valvular heart disease is associated with increased risk for adverse health events as a result of pregnancy.												
a. Uncomplicated	1		1		1		1		1		2	
b. Complicated (pulmonary hypertension, risk for atrial fibrillation, or history of subacute bacterial endocarditis)	1		1		1		2		1		4	
**Peripartum cardiomyopathy** This condition is associated with increased risk for adverse health events as a result of pregnancy.												
a. Normal or mildly impaired cardiac function (New York Heart Association Functional Class I or II: no limitation of activities or slight, mild limitation of activity) (*3*)												
i. <6 months	2		2		1		2		1		4	
ii. ≥6 months	2		2		1		2		1		3	
b. Moderately or severely impaired cardiac function (New York Heart Association Functional Class III or IV: marked limitation of activity or should be at complete rest) (*3*)	2		2		2		3		2		4	
**Renal Disease**												
**Chronic kidney disease** This condition is associated with increased risk for adverse health events as a result of pregnancy.	Initiation	Continuation	Initiation	Continuation	—							
a. Current nephrotic syndrome	1	1	2	2	2		3		2* DRSP POP with known hyperkalemia: 4*		4	
b. Hemodialysis	1	1	2	2	2		3		2* DRSP POP with known hyperkalemia: 4*		4	
c. Peritoneal dialysis	2	1	2	2	2		3		2* DRSP POP with known hyperkalemia: 4*		4	
**Rheumatic Diseases**												
**Systemic lupus erythematosus** This condition is associated with increased risk for adverse health events as a result of pregnancy.	Initiation	Continuation	—				Initiation	Continuation	—			
a. Positive (or unknown) antiphospholipid antibodies	1*	1*	2*		2*		3*	3*	2*		4*	
b. Severe thrombocytopenia	3*	2*	2*		2*		3*	2*	2*		2*	
c. Immunosuppressive therapy	2*	1*	2*		2*		2*	2*	2*		2*	
d. None of the above	1*	1*	2*		2*		2*	2*	2*		2*	
**Rheumatoid arthritis**	Initiation	Continuation	Initiation	Continuation	—							
a. Not receiving immunosuppressive therapy	1	1	1	1	1		2		1		2	
b. Receiving immunosuppressive therapy	2	1	2	1	1		2/3*		1		2	
**Neurologic Conditions**												
**Headaches**												
a. Nonmigraine (mild or severe)	1		1		1		1		1		1*	
b. Migraine												
i. Without aura (includes menstrual migraine)	1		1		1		1		1		2*	
ii. With aura	1		1		1		1		1		4*	
**Epilepsy** This condition is associated with increased risk for adverse health events as a result of pregnancy.	1		1		1*		1*		1*		1*	
**Multiple sclerosis**												
a. Without prolonged immobility	1		1		1		2		1		1	
b. With prolonged immobility	1		1		1		2		1		3	
**Depressive Disorders**												
**Depressive disorders**	1*		1*		1*		1*		1*		1*	
**Reproductive Tract Infections and Disorders**												
**Vaginal bleeding patterns**			Initiation	Continuation	—							
a. Irregular pattern without heavy bleeding	1		1	1	2		2		2		1	
b. Heavy or prolonged bleeding (includes regular and irregular patterns)	2*		1*	2*	2*		2*		2*		1*	
**Unexplained vaginal bleeding** (suspicious for serious condition) before evaluation	Initiation	Continuation	Initiation	Continuation	—							
	4*	2*	4*	2*	3*		3*		2*		2*	
**Endometriosis**	2		1		1		1		1		1	
**Benign ovarian tumors** (including cysts)	1		1		1		1		1		1	
**Severe dysmenorrhea**	2		1		1		1		1		1	
**Gestational trophoblastic disease** This condition is associated with increased risk for adverse health events as a result of pregnancy.												
a. Suspected gestational trophoblastic disease (immediate postevacuation)												
i. Uterine size first trimester	1*		1*		1*		1*		1*		1*	
ii. Uterine size second trimester	2*		2*		1*		1*		1*		1*	
b. Confirmed gestational trophoblastic disease (after initial evacuation and during monitoring)	Initiation	Continuation	Initiation	Continuation	—							
i. Undetectable or nonpregnant β-hCG levels	1*	1*	1*	1*	1*		1*		1*		1*	
ii. Decreasing β-hCG levels	2*	1*	2*	1*	1*		1*		1*		1*	
iii. Persistently elevated β-hCG levels or malignant disease, with no evidence or suspicion of intrauterine disease	2*	1*	2*	1*	1*		1*		1*		1*	
iv. Persistently elevated β-hCG levels or malignant disease, with evidence or suspicion of intrauterine disease	4*	2*	4*	2*	1*		1*		1*		1*	
**Cervical ectropion**	1		1		1		1		1		1	
**Cervical intraepithelial neoplasia**	1		2		2		2		1		2	
**Cervical cancer** (awaiting treatment)	Initiation	Continuation	Initiation	Continuation	—							
	4	2	4	2	2		2		1		2	
**Breast disease** Breast cancer is associated with increased risk for adverse health events as a result of pregnancy.												
a. Undiagnosed mass	1		2*		2*		2*		2*		2*	
b. Benign breast disease	1		1		1		1		1		1	
c. Family history of cancer	1		1		1		1		1		1	
d. Breast cancer												
i. Current	1		4		4		4		4		4	
ii. Past and no evidence of current disease for 5 years	1		3		3		3		3		3	
**Endometrial hyperplasia**	1		1		1		1		1		1	
**Endometrial cancer** This condition is associated with increased risk for adverse health events as a result of pregnancy.	Initiation	Continuation	Initiation	Continuation	—							
	4	2	4	2	1		1		1		1	
**Ovarian cancer** This condition is associated with increased risk for adverse health events as a result of pregnancy.	1		1		1		1		1		1	
**Uterine fibroids**	2		2		1		1		1		1	
**Anatomical abnormalities**												
a. Distorted uterine cavity (any congenital or acquired uterine abnormality distorting the uterine cavity in a manner that is incompatible with IUD placement)	4		4		—							
b. Other abnormalities (including cervical stenosis or cervical lacerations) not distorting the uterine cavity or interfering with IUD placement	2		2									
**Pelvic inflammatory disease**	Initiation	Continuation	Initiation	Continuation	—							
a. Current PID	4	2*	4	2*	1		1		1		1	
b. Past PID												
i. With subsequent pregnancy	1	1	1	1	1		1		1		1	
ii. Without subsequent pregnancy	2	2	2	2	1		1		1		1	
**Sexually transmitted infections**	Initiation	Continuation	Initiation	Continuation	—							
a. Current purulent cervicitis or chlamydial infection or gonococcal infection	4	2*	4	2*	1		1		1		1	
b. Vaginitis (including *Trichomonas vaginalis* and bacterial vaginosis)	2	2	2	2	1		1		1		1	
c. Other factors related to STIs	2*	2	2*	2	1		1		1		1	
**HIV**												
**High risk for HIV infection**	Initiation	Continuation	Initiation	Continuation	—							
	1*	1*	1*	1*	1		1		1		1	
**HIV infection** For persons with HIV infection who are not clinically well or not receiving ARV therapy, this condition is associated with increased risk for adverse health events as a result of pregnancy.	—	—	—	—	1*		1*		1*		1*	
a. Clinically well receiving ARV therapy	1	1	1	1	—		—		—		—	
b. Not clinically well or not receiving ARV therapy	2	1	2	1	—		—		—		—	
**Other Infections**												
**Schistosomiasis** Schistosomiasis with fibrosis of the liver is associated with increased risk for adverse health events as a result of pregnancy.												
a. Uncomplicated	1		1		1		1		1		1	
b. Fibrosis of the liver (if severe, see recommendations for Cirrhosis)	1		1		1		1		1		1	
**Tuberculosis** This condition is associated with increased risk for adverse health events as a result of pregnancy.	Initiation	Continuation	Initiation	Continuation	—							
a. Nonpelvic	1	1	1	1	1*		1*		1*		1*	
b. Pelvic	4	3	4	3	1*		1*		1*		1*	
**Malaria**	1		1		1		1		1		1	
**Endocrine Conditions**												
**Diabetes** Insulin-dependent diabetes; diabetes with nephropathy, retinopathy, or neuropathy; diabetes with other vascular disease; or diabetes of >20 years’ duration are associated with increased risk for adverse health events as a result of pregnancy.												
a. History of gestational disease	1		1		1		1		1		1	
b. Nonvascular disease												
i. Non-insulin dependent	1		2		2		2		2		2	
ii. Insulin dependent	1		2		2		2		2		2	
c. Nephropathy, retinopathy, or neuropathy	1		2		2		3		2		3/4*	
d. Other vascular disease or diabetes of >20 years’ duration	1		2		2		3		2		3/4*	
**Thyroid disorders**												
a. Simple goiter	1		1		1		1		1		1	
b. Hyperthyroid	1		1		1		1		1		1	
c. Hypothyroid	1		1		1		1		1		1	
**Gastrointestinal Conditions**												
**Inflammatory bowel disease** (ulcerative colitis or Crohn’s disease)	1		1		1		2		2		2/3*	
**Gallbladder disease**												
a. Asymptomatic	1		2		2		2		2		2	
b. Symptomatic												
i. Current	1		2		2		2		2		3	
ii. Treated by cholecystectomy	1		2		2		2		2		2	
iii. Medically treated	1		2		2		2		2		3	
**History of cholestasis**												
a. Pregnancy related	1		1		1		1		1		2	
b. Past COC related	1		2		2		2		2		3	
**Viral hepatitis**											Initiation	Continuation
a. Acute or flare	1		1		1		1		1		3/4*	2
b. Chronic	1		1		1		1		1		1	1
**Cirrhosis** Decompensated cirrhosis is associated with increased risk for adverse health events as a result of pregnancy.												
a. Compensated (normal liver function)	1		1		1		1		1		1	
b. Decompensated (impaired liver function)	1		2		2		3		2		4	
**Liver tumors** Hepatocellular adenoma and malignant liver tumors are associated with increased risk for adverse health events as a result of pregnancy.												
a. Benign												
i. Focal nodular hyperplasia	1		2		2		2		2		2	
ii. Hepatocellular adenoma	1		2		2		3		2		4	
b. Malignant (hepatocellular carcinoma)	1		3		3		3		3		4	
**Respiratory Conditions**												
**Cystic fibrosis** This condition is associated with increased risk for adverse health events as a result of pregnancy.	1*		1*		1*		2*		1*		1*	
**Hematologic Conditions**												
**Thalassemia**	2		1		1		1		1		1	
**Sickle cell disease** This condition is associated with increased risk for adverse health events as a result of pregnancy.	2		1		1		2/3*		1		4	
**Iron-deficiency anemia**	2		1		1		1		1		1	
**Solid Organ Transplantation**												
**Solid organ transplantation** This condition is associated with increased risk for adverse health events as a result of pregnancy.	Initiation	Continuation	Initiation	Continuation	**—**							
a. No graft failure	1	1	1	1	2		2/3*		2		2*	
b. Graft failure	2	1	2	1	2		2/3*		2		4	
**Drug Interactions**												
**Antiretrovirals used for prevention (PrEP) or treatment of HIV infection**												
See the following guidelines for the most up-to-date recommendations on drug-drug interactions between hormonal contraception and antiretrovirals: 1) Recommendations for the Use of Antiretroviral Drugs During Pregnancy and Interventions to Reduce Perinatal HIV Transmission in the United (https://clinicalinfo.hiv.gov/en/guidelines/perinatal/prepregnancy-counseling-childbearing-age-overview?view=full#table-3) (*5*) and 2) Guidelines for the Use of Antiretroviral Agents in Adults and Adolescents with HIV (https://clinicalinfo.hiv.gov/en/guidelines/hiv-clinical-guidelines-adult-and-adolescent-arv/drug-interactions-overview?view=full) (*6*).												
a. Nucleoside reverse transcriptase inhibitors (NRTIs)	Initiation	Continuation	Initiation	Continuation	—							
i. Abacavir (ABC)	1/2*	1*	1/2*	1*	1		1		1		1	
ii. Tenofovir (TDF)	1/2*	1*	1/2*	1*	1		1		1		1	
iii. Zidovudine (AZT)	1/2*	1*	1/2*	1*	1		1		1		1	
iv. Lamivudine (3TC)	1/2*	1*	1/2*	1*	1		1		1		1	
v. Didanosine (DDI)	1/2*	1*	1/2*	1*	1		1		1		1	
vi. Emtricitabine (FTC)	1/2*	1*	1/2*	1*	1		1		1		1	
vii. Stavudine (D4T)	1/2*	1*	1/2*	1*	1		1		1		1	
b. Nonnucleoside reverse transcriptase inhibitors (NNRTIs)												
i. Efavirenz (EFV)	1/2*	1*	1/2*	1*	2*		1*		2*		2*	
ii. Etravirine (ETR)	1/2*	1*	1/2*	1*	1		1		1		1	
iii. Nevirapine (NVP)	1/2*	1*	1/2*	1*	1		1		1		1	
iv. Rilpivirine (RPV)	1/2*	1*	1/2*	1*	1		1		1		1	
c. Ritonavir-boosted protease inhibitors												
i. Ritonavir-boosted atazanavir (ATV/r)	1/2*	1*	1/2*	1*	2*		1*		2*		2*	
ii. Ritonavir-boosted darunavir (DRV/r)	1/2*	1*	1/2*	1*	2*		1*		2*		2*	
iii. Ritonavir-boosted fosamprenavir (FPV/r)	1/2*	1*	1/2*	1*	2*		1*		2*		2*	
iv. Ritonavir-boosted lopinavir (LPV/r)	1/2*	1*	1/2*	1*	1		1		1		1	
v. Ritonavir-boosted saquinavir (SQV/r)	1/2*	1*	1/2*	1*	2*		1*		2*		2*	
vi. Ritonavir-boosted tipranavir (TPV/r)	1/2*	1*	1/2*	1*	2*		1*		2*		2*	
d. Protease inhibitors without ritonavir												
i. Atazanavir (ATV)	1/2*	1*	1/2*	1*	1		1		1		2*	
ii. Fosamprenavir (FPV)	1/2*	1*	1/2*	1*	2*		2*		2*		3*	
iii. Indinavir (IDV)	1/2*	1*	1/2*	1*	1		1		1		1	
iv. Nelfinavir (NFV)	1/2*	1*	1/2*	1*	2*		1*		2*		2*	
e. CCR5 co-receptor antagonists												
i. Maraviroc (MVC)	1/2*	1*	1/2*	1*	1		1		1		1	
f. HIV integrase strand transfer inhibitors												
i. Raltegravir (RAL)	1/2*	1*	1/2*	1*	1		1		1		1	
ii. Dolutegravir (DTG)	1/2*	1*	1/2*	1*	1		1		1		1	
iii. Elvitegravir (EVG)	1/2*	1*	1/2*	1*	1		1		1		1	
g. Fusion inhibitors												
i. Enfuvirtide	1/2*	1*	1/2*	1*	1		1		1		1	
**Anticonvulsant therapy**												
a. Certain anticonvulsants (phenytoin, carbamazepine, barbiturates, primidone, topiramate, and oxcarbazepine)	1		1		2*		1*		3*		3*	
b. Lamotrigine	1		1		1		1		1		3*	
**Antimicrobial therapy**												
a. Broad-spectrum antibiotics	1		1		1		1		1		1	
b. Antifungals	1		1		1		1		1		1	
c. Antiparasitics	1		1		1		1		1		1	
d. Rifampin or rifabutin therapy	1		1		2*		1*		3*		3*	
**Psychotropic medications**												
a. Selective serotonin reuptake inhibitors (SSRIs)	1		1		1		1		1		1	
**St. John’s wort**	1		1		2		1		2		2	

**Abbreviations:** ARV = antiretroviral; BMI = body mass index; CHC = combined hormonal contraceptive; COC = combined oral contraceptive; Cu-IUD = copper intrauterine device; DMPA = depot medroxyprogesterone acetate; DRSP = drospirenone; DVT = deep venous thrombosis; hCG = human chorionic gonadotropin; HDL = high-density lipoprotein; IUD = intrauterine device; LDL = low-density lipoprotein; LNG-IUD = levonorgestrel intrauterine device; NA = not applicable; PE = pulmonary embolism; PID = pelvic inflammatory disease; POP = progestin-only pill; PrEP = pre-exposure prophylaxis; STI = sexually transmitted infection; VTE = venous thromboembolism.

* Consult the respective appendix for each contraceptive method in *U.S. Medical Eligibility Criteria for Contraceptive Use, 2024* (*1*) for clarifications to the numeric categories.

## Appendix B When To Start Using Specific Contraceptive Methods

This appendix summarizes recommendations for when to start using specific contraceptive methods (Table B1).

**TABLE B1:** When to start using specific contraceptive methods

Contraceptive method	When to start (if the provider is reasonably certain that the patient is not pregnant)*	Additional contraception (i.e., back-up) needed	Examination or test needed before initiation^†^
Cu-IUD	Anytime	Not needed	Bimanual examination and cervical inspection^§^
LNG-IUD	Anytime	If >7 days after menses started, abstain from sexual intercourse or use barrier methods (e.g., condoms) for 7 days	Bimanual examination and cervical inspection^§^
Implant	Anytime^¶^	If >5 days after menses started, abstain from sexual intercourse or use barrier methods (e.g., condoms) for 7 days	None
DMPA	Anytime^¶^	If >7 days after menses started, abstain from sexual intercourse or use barrier methods (e.g., condoms) for 7 days	None
CHC	Anytime^¶^	If >5 days after menses started, abstain from sexual intercourse or use barrier methods (e.g., condoms) for 7 days	Blood pressure measurement
Norethindrone or norgestrel POP	Anytime^¶^	If >5 days after menses started, abstain from sexual intercourse or use barrier methods (e.g., condoms) for 2 days	None
Drospirenone POP	Anytime^¶^	If >1 day after menses started, abstain from sexual intercourse or use barrier methods (e.g., condoms) for 7 days	None

**Abbreviations:** BMI = body mass index; CHC = combined hormonal contraceptive; Cu-IUD = copper intrauterine device; DMPA = depot medroxyprogesterone acetate; IUD = intrauterine device; LNG-IUD = levonorgestrel intrauterine device; POP = progestin-only pill; STI = sexually transmitted infection; U.S. MEC = *U.S. Medical Eligibility Criteria for Contraceptive Use*.

* As appropriate, see recommendations for Emergency Contraception.

^†^ Weight (BMI) measurement is not needed to determine medical eligibility for any methods of contraception because all methods can be used (U.S. MEC 1) or generally can be used (U.S. MEC 2) among patients with obesity (BMI ≥30 kg/m^2^). However, measuring weight and calculating BMI (weight [kg]/height [m]^2^) at baseline might be helpful for discussing concerns about any changes in weight and whether changes might be related to use of the contraceptive method.

^§^ Most patients do not require additional STI screening at the time of IUD placement. If a patient with risk factors for STIs has not been screened for gonorrhea and chlamydia according to CDC’s *Sexually Transmitted Infections Treatment Guidelines* (https://www.cdc.gov/std/treatment-guidelines/default.htm), screening may be performed at the time of IUD placement, and placement should not be delayed. Patients with current purulent cervicitis or chlamydial infection or gonococcal infection should not undergo IUD placement (U.S. MEC 4).

^¶^ In situations in which the health care provider is uncertain whether the patient might be pregnant, the benefits of starting the implant, DMPA, CHC, and POP likely exceed any risk; therefore, starting the implant, DMPA, CHC, and POP should be considered at any time, with a follow-up pregnancy test in 2–4 weeks.

## Appendix C Examinations and Tests Needed Before Initiation of Contraceptive Methods

The examinations and tests noted apply to patients who are presumed to be healthy (Table C1). Those with known medical problems or other special conditions might need additional examinations and tests before being determined to be appropriate candidates for a particular method of contraception. *U.S. Medical Eligibility Criteria for Contraceptive Use, 2024* (U.S. MEC) might be useful in such circumstances (*1*). The following classification was considered useful in differentiating the applicability of the various examinations and tests (*2*):

**Class A:** Essential and mandatory in all circumstances for safe and effective use of the contraceptive method.
**Class B:** Contributes substantially to safe and effective use, but implementation may be considered within the public health context, service context, or both; risk of not performing an examination or test should be balanced against the benefits of making the contraceptive method available.
**Class C:** Does not contribute substantially to safe and effective use of the contraceptive method.

These classifications focus on the relation of the examinations or tests to safe initiation of a contraceptive method. They are not intended to address the appropriateness of these examinations or tests in other circumstances. For example, certain examinations or tests that are not deemed necessary for safe and effective contraceptive use might be appropriate for good preventive health care or for diagnosing or assessing suspected medical conditions. Any additional screening needed for preventive health care can be performed at the time of contraception initiation, and initiation should not be delayed for test results.

No examinations or tests are needed before initiating condoms, spermicides, or vaginal pH modulators. A bimanual examination is necessary for diaphragm fitting. A bimanual examination and cervical inspection are needed for cervical cap fitting.

**TABLE C1:** Examinations and tests needed before initiation of contraceptive methods

Examination or test	Contraceptive method and class							
	Cu-IUD and LNG-IUD	Implant	DMPA	CHC	POP	Condom	Spermicide and vaginal pH modulator	Diaphragm/Cap (with spermicide)
**Examination**								
Blood pressure	C	C	C	A*	C	C	C	C
Weight (BMI) (weight [kg]/height [m]^2^)	—^†^	—^†^	—^†^	—^†^	—^†^	C	C	C
Clinical breast examination	C	C	C	C	C	C	C	C
Bimanual examination and cervical inspection	A	C	C	C	C	C	C	A^§^
**Laboratory test**								
Glucose	C	C	C	C	C	C	C	C
Lipids	C	C	C	C	C	C	C	C
Liver enzymes	C	C	C	C	C	C	C	C
Hemoglobin	C	C	C	C	C	C	C	C
Thrombophilia	C	C	C	C	C	C	C	C
Cervical cytology (Papanicolaou test)	C	C	C	C	C	C	C	C
STI screening with laboratory tests	—^¶^	C	C	C	C	C	C	C
HIV screening with laboratory tests	C	C	C	C	C	C	C	C

**Abbreviations:** BMI = body mass index; CHC = combined hormonal contraceptive; Cu-IUD = copper intrauterine device; DMPA = depot medroxyprogesterone acetate; IUD = intrauterine device; LNG-IUD = levonorgestrel intrauterine device; POP = progestin-only pill; STI = sexually transmitted infection; U.S. MEC = *U.S. Medical Eligibility Criteria for Contraceptive Use*.

* In instances in which blood pressure cannot be measured by a provider, blood pressure measured in other settings can be reported by the patient to their provider.

^†^ Weight (BMI) measurement is not needed to determine medical eligibility for any methods of contraception because all methods can be used (U.S. MEC 1) or generally can be used (U.S. MEC 2) among patients with obesity (BMI ≥30 kg/m^2^). However, measuring weight and calculating BMI at baseline might be helpful for discussing concerns about any changes in weight and whether changes might be related to use of the contraceptive method.

^§^ A bimanual examination (not cervical inspection) is needed for diaphragm fitting.

^¶^ Most patients do not require additional STI screening at the time of IUD placement. If a patient with risk factors for STIs has not been screened for gonorrhea and chlamydia according to CDC’s *Sexually Transmitted Infections Treatment Guidelines* (https://www.cdc.gov/std/treatment-guidelines/default.htm), screening may be performed at the time of IUD placement, and placement should not be delayed. Patients with current purulent cervicitis or chlamydial infection or gonococcal infection should not undergo IUD placement (U.S. MEC 4).

## Appendix D Routine Follow-Up After Contraceptive Initiation

This appendix addresses when routine follow-up is recommended for safe and effective continued use of contraception for healthy patients (Table D1). The recommendations refer to general situations and might vary for different users and different situations. Specific populations who might benefit from more frequent follow-up visits include adolescents, those with certain medical conditions or characteristics, and those with multiple medical conditions.

**TABLE D1:** Routine follow-up actions after contraceptive initiation

Action	Contraceptive method				
	Cu-IUD or LNG-IUD	Implant	DMPA	CHC	POP
**General follow-up**					
Advise the patient that they may contact their provider at any time to discuss side effects or other problems or if they want to change the method. Advise patients using IUDs, implants, or DMPA when the IUD or implant needs to be removed or when a reinjection is needed. No routine follow-up visit is required.	X*	X*	X*	X*	X*
**Other routine visits**					
Assess the patient’s satisfaction with their current method and whether they have any concerns about method use.	X*	X*	X*	X*	X*
Assess any changes in health status, including medications, that would change the method’s appropriateness for safe and effective continued use on the basis of U.S. MEC (i.e., category 3 and 4 conditions and characteristics) (Box 2).	X*	X*	X*	X*	X*
Consider performing an examination to check for the presence of IUD strings.	X*	—^†^	—^†^	—^†^	—^†^
Consider assessing weight changes and discussing concerns about any changes in weight and whether changes might be related to use of the contraceptive method.	X*	X*	X*	X*	X*
Measure blood pressure.	—^†^	—^†^	—^†^	X*	—^†^

**Abbreviations:** CHC = combined hormonal contraceptive; Cu-IUD = copper intrauterine device; DMPA = depot medroxyprogesterone acetate; IUD = intrauterine device; LNG-IUD = levonorgestrel intrauterine device; POP = progestin-only pill; U.S. MEC = *U.S. Medical Eligibility Criteria for Contraceptive Use*.

* The action is applicable to the contraceptive method.

^†^ The action is not applicable to the contraceptive method.

## Appendix E Management of Bleeding Irregularities While Using Contraception

This appendix summarizes recommendations for management of bleeding irregularities while using contraception (Figure E1).

## Appendix F Management of Intrauterine Devices When Users Are Found To Have Pelvic Inflammatory Disease

This appendix summarizes recommendations for management of intrauterine devices when users are found to have pelvic inflammatory disease (Figure F1).
